# Flow-mediated endothelial remodeling and inflammation drive developmental vascular susceptibility in *ldlr* loss of function

**DOI:** 10.1038/s41467-026-72756-3

**Published:** 2026-05-21

**Authors:** Aryan Kaveh, Antonio G. Salazar-Martin, Wei Dai, Aaron P. Kithcart, Emily Pan, Ashmita KC, Tyler R. Reinoso, Franki Vetrano-Olsen, Kusumika Saha, Manu Beerens, Peter Libby, Elazer R. Edelman, Calum A. MacRae

**Affiliations:** 1 Division of Cardiovascular Medicine, Department of Medicine, Brigham and Women’s Hospital, Harvard Medical School, Boston, MA USA; 2 Stem Cells and Metabolism Research Program, Faculty of Medicine, University of Helsinki, Helsinki, Finland; 3 Institute for Medical Engineering and Science, Massachusetts Institute of Technology (MIT), Cambridge, MA USA; 4 Broad Institute of MIT and Harvard, Cambridge, MA USA

**Keywords:** Disease model, Angiogenesis, Mechanisms of disease

## Abstract

Atherosclerosis, the leading cause of cardiovascular disease, is associated with aberrant lipid metabolism, endothelial dysfunction, and chronic inflammation, yet its early manifestations and mechanisms remain incompletely understood. As low-density lipoprotein receptor loss of function is the most common monogenic cause of atherosclerosis, we employed low-density lipoprotein receptor knockout (*ldlr*-/-) zebrafish to investigate the developmental origins of atherosclerotic cardiovascular disease. Single-cell RNA-sequencing under differential flow conditions in embryonic *ldlr*-/- zebrafish identified a population of disproportionately stressed endothelial cells marked by overexpression of heat shock protein 70 (*hsp70*). *Hsp70* is induced in stressed endothelial cells in a flow-dependent manner in zebrafish and a subset of human endothelial cells, and its activation is associated with disrupted remodeling angiogenesis in vivo. Genetic and pharmacological studies demonstrated that *hsp70* upregulation inhibits vascular apoptosis and ciliogenesis, leading to altered angiogenic remodeling. Concurrently, pro-inflammatory processes, including enhanced myelopoiesis and thrombogenicity, are amplified at early stages in *ldlr*-/- zebrafish, which also exhibit impaired regenerative angiogenesis and heightened neutrophil recruitment post-vascular injury. Our findings reveal how abnormalities in flow-mediated endothelial remodeling and inflammation converge during embryogenesis to drive vascular susceptibility to hemodynamic and other stressors in *ldlr* loss of function.

## Abstract (web summary)

Here they show that blood flow and early endothelial stress responses interact to drive abnormal angiogenic remodeling and inflammation, revealing how vascular disease susceptibility can arise during development.

## Introduction

Atherosclerotic cardiovascular disease (ASCVD) is driven by low-density lipoprotein (LDL) cholesterol accumulation within the subendothelial compartment of arteries^1^. The progression of atherosclerosis is regulated by complex biological processes, resulting in significant disease heterogeneity with various clinical subtypes and risk factors^2,3^. Monogenic and polygenic forms of atherosclerosis are characterized by aberrant lipid trafficking and metabolism, endothelial dysfunction, and chronic inflammation, all of which contribute to plaque formation^3,4^. During plaque growth and the progression of atherosclerosis, dysfunctional endothelial cells recruit immune cells that become lipid-laden and secrete inflammatory mediators, amplifying plaque expansion and the risk of thrombosis^5^. Recent work has also implicated dysregulated hematopoietic clones among the drivers of chronic inflammation and ASCVD events^6^.

Despite these important insights into the later stages of ASCVD, cellular mechanisms underlying the early phases of atherosclerosis remain incompletely understood. Biomechanical forces exerted by blood flow, or shear stress, maintain vascular health and modulate endothelial signaling and inflammatory responses to initial vascular injury, thereby influencing atherosclerosis progression^7,8^. Regions exposed to low shear stress or disturbed flow, such as vascular bifurcations, are more prone to atherosclerosis, potentially due to enhanced endothelial turnover, differential lipid uptake, or inflammatory activation^9–11^. In contrast, high or laminar shear stress generally protects against atherosclerosis by promoting endothelial quiescence and homeostasis^9,12,13^. Understanding the upstream mechanisms of atherosclerosis, prior to plaque formation, could inform early preventive strategies for susceptible individuals.

Mutations in the LDL receptor (LDLR), which mediates LDL endocytosis, cause familial hypercholesterolemia (FH), the most common monogenic form of atherosclerosis^14,15^. Homozygous FH results in severe hypercholesterolemia from early childhood and confers a markedly increased risk of premature coronary artery disease^16,17^. Although *Ldlr* knockout animals are well established, the early developmental consequences of LDLR deficiency remain poorly characterized in vivo. Owing to their experimental tractability, developing zebrafish are a powerful model to investigate disease onset and evolution, as many cellular and molecular mechanisms are conserved. Notably, this includes the trunk vascular system, which undergoes regulated structural remodeling and hemodynamically-guided patterning^18–20^. To investigate the biological features of atherosclerosis from early development onward, we therefore used *ldlr* knockout zebrafish^21^. We hypothesized that primary endothelial cell abnormalities arising from LDLR loss of function underpin the earliest stages of atherosclerosis by initiating vascular dysfunction and inflammation.

Here, using longitudinal phenotyping we show that embryonic *ldlr* knockout zebrafish exhibit altered flow-dependent vascular remodeling during primary angiogenesis. This abnormal remodeling is associated with a subpopulation of mechanoresponsive stressed endothelial cells in vivo, a finding corroborated in vitro in flow-dependent human endothelial cell responses. Early endothelial abnormalities in *ldlr* knockout zebrafish are accompanied by enhanced myelopoiesis, increased thrombogenicity and impaired regenerative angiogenesis. Our findings suggest that vascular susceptibility in *ldlr* loss of function originates during embryogenesis, with core ASCVD features, such as endothelial dysfunction and inflammatory activation impacting both physiologic angiogenic remodeling and the response to injury.

## Results

### *Ldlr* loss disrupts angiogenic remodeling in developing zebrafish

**Fig. 1:**
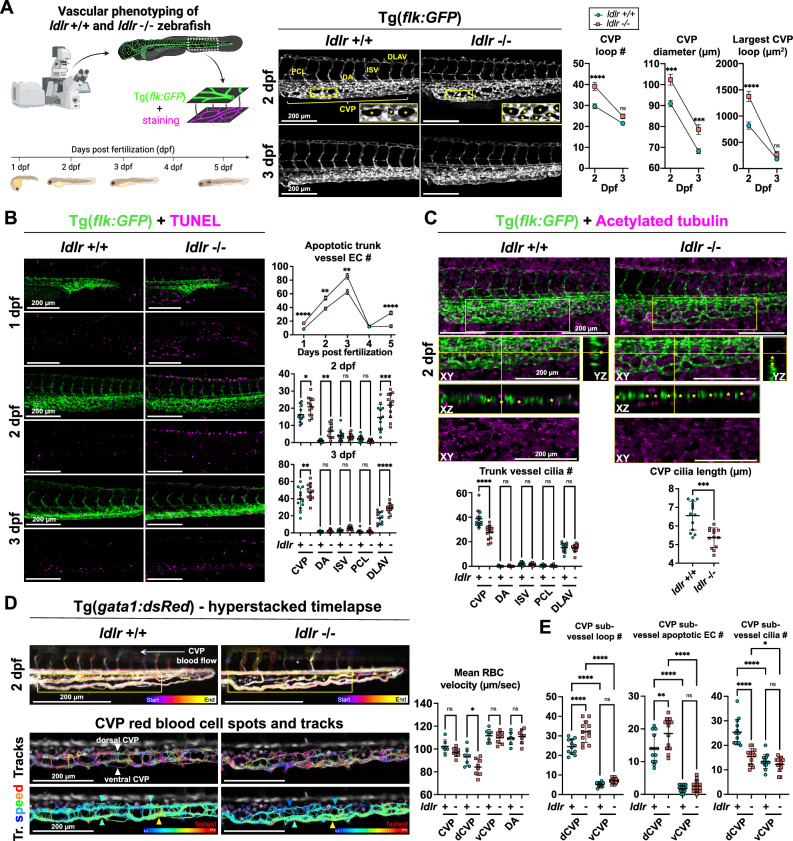
Embryonic *ldlr*-/- zebrafish display disrupted remodeling angiogenesis, enhanced endothelial apoptosis and reduced ciliogenesis. **A** Schematic of developmental vascular phenotyping in *ldlr* + /+ and *ldlr*-/- zebrafish from 1-5 days post fertilization (dpf). Created in BioRender. Salazar, A. (2026) https://BioRender.com/7fk7msr (left). Confocal microscopy of Tg(*flk:GFP*) trunk vasculature at 2 dpf and 3 dpf. Trunk vessels from dorsal to ventral locations: DLAV (dorsal longitudinal anastomotic vessel), ISV (intersegmental vessel), PCL (parachordal lymphatic), DA (dorsal aorta) and CVP (caudal venous plexus). CVP indicated as a bifurcated vascular network and insets show CVP loops (asterisks); transient hollow structures remodeled at 3 dpf (middle). CVP metrics in *ldlr* + /+ (*n* = 12) and *ldlr*-/- zebrafish (*n* = 12): loop number at 2 dpf (*p* < 0.0001) and 3 dpf (*p* = 0.0793), diameter (μm) at 2 dpf (*p* = 0.0003) and 3 dpf (*p* = 0.0006) and largest loop area (μm^2^) at 2 dpf (*p* < 0.0001) and 3 dpf (*p* = 0.4036); 3 independent experiments. Data are mean ± s.e.m. Two-way ANOVA and Holm-Sidak’s multiple comparison used (right). **B** Confocal microscopy of TUNEL-stained trunk vasculature in *ldlr* + /+ and *ldlr*-/- zebrafish at 1-3 dpf (left). Number of apoptotic endothelial cells in the trunk vasculature of *ldlr* + /+ (*n* = 12-13) and *ldlr*-/- (*n* = 12-14) zebrafish at 1 dpf (*p* < 0.0001), 2 dpf (*p* = 0.0020), 3 dpf (*p* = 0.0020), 4 dpf (*p* = 0.7023) and 5 dpf (*p* < 0.0001); 3 independent experiments. Data are mean ± s.e.m. Two-way ANOVA and Holm-Sidak’s multiple comparison used (top, right). Number of apoptotic endothelial cells in trunk vessels between *ldlr* + /+ (*n* = 12) and *ldlr*-/- (*n* = 12) zebrafish at 2 dpf (middle, right) a*n*d 3 dpf (bottom, right). CVP 2 dpf (*p* = 0.0366) and 3 dpf (*p* = 0.0039), DA 2 dpf (*p* = 0.0085) and 3 dpf (*p* = 0.9419), ISV 2 d*p*f (*p* = 0.7391) and 3 d*p*f (*p* = 0.6444), PCL 2 d*p*f (*p* = 0.7391) and 3 d*p*f (*p* = 0.9728), DLAV 2 d*p*f (*p* = 0.0006) and 3 d*p*f (*p* < 0.0001); 3 independent experiments. Data are mean ± s.d. One-way ANOVA and Holm-Sidak’s multiple comparison used. **C** Confocal microscopy of acetylated tubulin-stained trunk vasculature in *ldlr* + /+ and *ldlr*-/- zebrafish at 2 dpf. Insets show maximum intensity projections (XY) and orthogonal z-slices (XZ and YZ planes) of CVP loops (asterisks) and associated cilia (middle). Trunk vessel cilia number (bottom left) and average CVP cilia length (bottom right, μm) in *ldlr* + /+ (*n* = 13) and *ldlr*-/- (*n* = 13) zebrafish. Cilia number: CVP (*p* < 0.0001), DA (*p* > 0.9999), ISV (*p* = 0.9971), PCL (*p* = 0.9971), DLAV (*p* = 0.0006). CVP length (*p* = 0.0001); 3 independent experiments. Data are mean ± s.d. One-way ANOVA and Holm-Sidak’s multiple comparison (bottom, left) and unpaired two-tailed* t* test used (bottom, right). **D** Red blood cell movement analysis in Tg(*gata1:dsRed*) trunk vasculature of *ldlr* + /+ and *ldlr*-/- zebrafish at 2 dpf. Hyperstacked time-lapse of red blood cell motility in the trunk vasculature, summarizing movement routes and activity relative to time (color-coded). Insets indicate CVP region used for tracking (top, left). Tracking of CVP red blood cells (spots) and analysis of movement routes (tracks). Red blood cell movement tracks through the dorsal CVP (dCVP, downward arrowheads) and ventral CVP (vCVP, upward arrowheads) are overlaid, and track speeds are color-coded (bottom, left). Mean red blood cell velocity (μm/sec) in the CVP, dCVP, vCVP and DA of *ldlr* + /+ (*n* = 7) and *ldlr*-/- (*n* = 8) zebrafish. CVP (*p* = 0.2545), dCVP (*p* = 0.0242), vCVP (*p* = 0.8216), DA (*p* = 0.8216); 2 independent experiments. Data are mean ± s.d. One-way ANOVA and Holm-Sidak’s multiple comparison used (right). **E** Quantification of dCVP and vCVP number (left), apoptotic EC number (middle), and cilia number (right) in *ldlr* + /+ (*n* = 12-13) and *ldlr*-/- zebrafish (*n* = 12-13) at 2 dpf. dCVP *ldlr* + /+ vs dCVP *ldlr*-/- (loop number: *p* < 0.0001, apoptosis: *p* = 0.0012, cilia number: *p* < 0.0001), vCVP *ldlr* + /+ vs vCVP *ldlr*-/- (loop number: *p* = 0.1918, apoptosis: *p* = 0.5353, cilia number: *p* = 0.3782), dCVP *ldlr* + /+ vs vCVP *ldlr* + /+ (loop number: *p* < 0.0001, apoptosis: *p* < 0.0001, cilia number: *p* < 0.0001), dCVP *ldlr*-/- vs vCVP *ldlr*-/- (loop number: *p* < 0.0001, apoptosis: *p* < 0.0001, cilia number: *p* = 0.0392); 3 independent experiments. Data are mean ± s.d. Two-way ANOVA and Holm-Sidak’s multiple comparison used. Source data are provided as a Source Data file.

To characterize vascular development, we first performed time-series confocal imaging from 1 day post fertilization (dpf) in wild-type (*ldlr* + /+) and homozygous *ldlr* loss of function (*ldlra*^*sd52/sd52*^^21^, hereafter referred to as *ldlr*-/-) zebrafish expressing Tg(*flk:GFP*). Serial 24 h vascular imaging was conducted up to 5 dpf, when hyperlipidemia and systemic LDL elevation are apparent in *ldlr*-/- zebrafish^21^ (Supplementary Fig. 1A, B). Vascular imaging at 2 dpf detected abnormal vessel patterning in *ldlr*-/- embryos, specifically within the caudal venous plexus (CVP). Unlike their *ldlr* + /+ counterparts, the CVP of *ldlr*-/- embryos displayed abnormal remodeling angiogenesis, characterized by an increased number of CVP loops, larger loops, and a wider CVP diameter (Fig. 1A). While the bifurcated CVP remodeled to a tube-like structure at 3 dpf in *ldlr* + /+ controls, *ldlr*-/- zebrafish retained a larger caudal vein diameter (Fig. 1A). No distinct morphological differences were observed between genotypes in the trunk vasculature at 1 dpf, 4 dpf, or 5 dpf (Supplementary Fig. 1C) or in the cerebral vessels (Supplementary Fig. 2A).

We next used whole-mount immunofluorescence staining to further assess vascular phenotypes in vivo. At 2 dpf, *ldlr-/-* embryos exhibited an excess of apolipoprotein B (apoB) particles, an early indicator of atherosclerosis^22^, which returned to *ldlr* + /+ levels by 3 dpf (Supplementary Fig. 3). TUNEL staining showed increased trunk vessel apoptosis in *ldlr*-/- zebrafish at 1 dpf, 2 dpf, 3 dpf and 5 dpf (Fig. 1B and Supplementary Fig. 2B, C). Endothelial cell (EC) death was specifically increased in the CVP and dorsal aorta (DA) at 1 dpf; the CVP, DA and dorsal longitudinal anastomotic vessel (DLAV) at 2 dpf; and the CVP and DLAV at 3 dpf (Supplementary Fig. 2C and Fig. 1B). Apoptotic EC activity remained unchanged in the cerebral vasculature of *ldlr-/*- embryos and larvae (Supplementary Fig. 2A), demonstrating endothelial abnormalities were predominantly localized to the trunk vasculature.

PCNA staining identified a greater number of proliferating CVP ECs in *ldlr*-/- zebrafish at 2 dpf, but not at 1 dpf or 3 dpf, suggesting a transient normalization of CVP EC turnover (proliferation-to-apoptosis ratio) in homozygotes at 2 dpf (Supplementary Fig. 4). Given ECs exhibit mechanosensory cilia that regulate angiogenesis and cell turnover, we next assessed ciliogenesis using acetylated tubulin staining^23^. Vascular endothelial cilia were observed predominantly within the developing CVP, adjacent to vascular loops (Fig. 1 and Supplementary Fig. 5). Markedly fewer and shorter CVP cilia were detected in *ldlr*-/- embryos at 2 dpf specifically, coinciding with abnormal remodeling angiogenesis (Fig. 1C, Supplementary Fig. 5).

To assess other mechanosensory phenotypes in *ldlr*-/- zebrafish, we examined the ciliated posterior lateral line neuromasts using DASPEI (mitochondrial hair cell dye) and BODIPY FL C5 ceramide (sphingolipid golgi dye). Intravital imaging at 2 dpf demonstrated reduced neuromast hair cell staining in *ldlr*-/- embryos (Supplementary Fig. 6), suggestive of mechanosensory abnormalities outside of the vasculature.

Since blood flow is a key regulator of vascular remodeling, we tracked red blood cell (RBC) movement by live imaging Tg(*gata1:dsRed*) *ldlr* + /+ and *ldlr*-/- zebrafish to define trunk vessel flow patterns. As expected, RBC velocity was higher in the DA compared to the CVP, and increased across development (Supplementary Fig. 2D and Supplementary Movie 1, 2). At 2 dpf, overall CVP RBC velocity was comparable between *ldlr* + /+ (Supplementary Movie 1) and *ldlr*-/- zebrafish (Supplementary Movie 3**)**, however, distinct spatial differences emerged within CVP sub-regions (Fig. 1D). In both genotypes, flow through the ventral CVP (vCVP) was fast and uniform, whereas the dorsal CVP (dCVP) of *ldlr*-/- zebrafish exhibited reduced velocity and a more complex flow profile (Fig. 1D). Other notable phenotypes were quantified in these sub-regions and spatially related with altered CVP anatomy and blood flow patterns. Consistent with the localized flow disturbances, the dCVP of *ldlr*-/- zebrafish displayed an increased number of remodeling loops, elevated EC apoptosis, and a significant reduction in ciliated ECs, identifying this specific region as the major site of defective remodeling angiogenesis (Fig. 1E).

Altogether, developmental phenotyping of *ldlr*-/- zebrafish revealed dysregulated remodeling angiogenesis associated with disturbed blood flow, increased endothelial apoptosis and reduced ciliogenesis.

### scRNA-seq identifies *hsp70*+ stressed ECs in *ldlr*-/- zebrafish

**Fig. 2:**
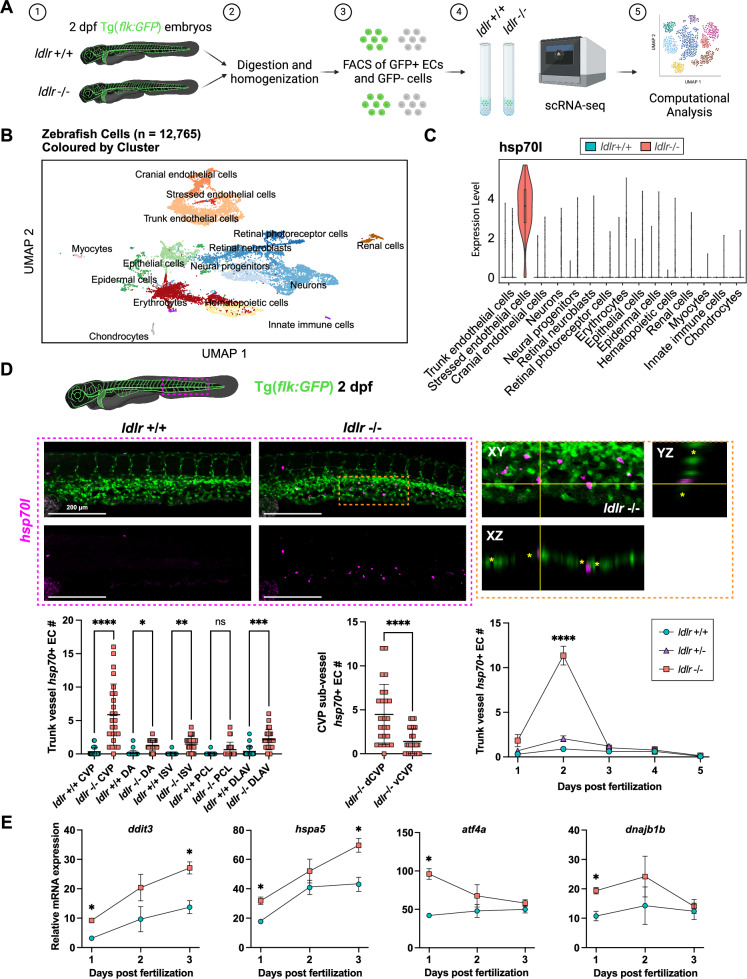
scRNA-sequencing of embryonic *ldlr*-/- zebrafish identifies a subpopulation of stressed endothelial cells marked by *hsp70* upregulation. **A** Schematic of scRNA-seq workflow in Tg(*flk:GFP*) *ldlr* + /+ and *ldlr*-/- zebrafish at 2 dpf. Created in BioRender. Salazar, A. (2026) https://BioRender.com/y95wgtg. **B** Cluster uniform manifold approximation and projection (UMAP) of 12,765 cells colored by annotated cell populations. Cells were obtained from pooled embryos (*n* = 200 zebrafish per genotype) processed in a single batch. **C** Violin plot of normalized *hsp70l* (cytoplasmic chaperone/unfolded protein response) expression across cell populations in *ldlr* + /+ and *ldlr*-/- zebrafish. Each violin represents single-cell expression values, the central line indicates the median, and the shape represents the kernel distribution. **D** Confocal microscopy of *hsp70l* fluorescence in situ RNA hybridization-stained trunk vasculature in *ldlr* + /+ and *ldlr*-/- zebrafish at 2 dpf (top, left). Inset shows maximum intensity projection (XY) and orthogonal z-slices (XZ and YZ planes) of *hsp70 *+ endothelial cells adjacent to caudal venous plexus (CVP) loops (asterisks) in *ldlr*-/- zebrafish (top, right). Number of trunk vessel *hsp70+* endothelial cells between *ldlr* + /+ (*n* = 27) and *ldlr*-/- (*n* = 25) zebrafish. DA (dorsal aorta), ISV (intersegmental vessel), PCL (parachordal lymphatic), DLAV (dorsal longitudinal anastomotic vessel) at 2 dpf. CVP (*p* < 0.0001), DA (*p* = 0.0261), ISV (*p* = 0.0048), PCL (*p* = 0.1833), DLAV (*p* = 0.0006); 4 independent experiments. Data are mean ± s.d. One-way ANOVA and Holm-Sidak’s multiple comparison used (bottom, left). CVP sub-vessel (dCVP and vCVP) *hsp70 *+ endothelial cell number in *ldlr* + /+ (*n* = 25) and *ldlr*-/- (*n* = 25), *p* = 0.0001; 4 independent experiments. Data are mean ± s.d. Paired two-tailed *t* test used (bottom, middle). Number of trunk vessel *hsp70 *+ endothelial cells in *ldlr* + /+ (*n* = 13-29), *ldlr* + /- (*n* = 13-27) and *ldlr*-/- (*n* = 12-29) zebrafish at 1 dpf (*p* = 0.1774), 2 dpf (*p* < 0.0001), 3 dpf (*p* = 0.5423), 4 dpf (*p* = 0.8054), 5 dpf (*p* = 0.8054); 4 independent experiments. Data are mean ± s.e.m. Two-way ANOVA and Holm-Sidak’s multiple comparison used (bottom, right). **E** RT-qPCR analysis of cell stress response genes: *ddit3* (endoplasmic reticulum stress), *hspa5* (endoplasmic reticulum chaperone), *atf4a* (endoplasmic reticulum stress) and *dnajb1b* (cytoplasmic chaperone/unfolded protein response) in *ldlr* + / + and *ldlr*-/- zebrafish 1-3 dpf. 1 dpf: *ddit3* (*p* = 0.0151), *hspa5* (*p* = 0.0457), *atf4a* (*p* = 0.0326), *dnajb1b* (*p* = 0.0456), 2 dpf: *ddit3* (*p* = 0.1594), *hspa5* (*p* = 0.3134), *atf4a* (*p* = 0.4853), *dnajb1b* (*p* = 0.5836) and 3 dpf: *ddit3* (*p* = 0.0234), *hspa5* (*p* = 0.0457), *atf4a* (*p* = 0.4853), *dnajb1b* (*p* = 0.6709). Expression was normalized to *gapdh*. Each replicate consists of *n* = 14–20 pooled zebrafish per group; 3 independent experiments. Data are represented as mean ± s.e.m. Two-way ANOVA and Holm-Sidak’s multiple comparison used. Source data are provided as a Source Data file.

To better understand the endothelial cell biology underlying the vascular defects identified in *ldlr*-/- zebrafish, we performed single-cell RNA sequencing (scRNA-seq) of 2 dpf *ldlr* + /+ and *ldlr*-/- embryos. We isolated *flk*:GFP + ECs and included *flk*:GFP- cells (non-endothelial population) to enrich for ECs while capturing a representative range of other cell types (Fig. 2A and Supplementary Fig. 7). Transcriptional analysis distinguished 15 cell clusters representing 10 distinct cell types (Fig. 2B, Supplementary Fig. 8A and Supplementary Data 1). One additional trunk EC cluster that was markedly enriched for stress response genes was identified in *ldlr*-/- embryos (Fig. 2B and Supplementary Fig. 8A, B). This stressed EC subpopulation was characterized by substantial upregulation of four *hsp70* genes (*hsp70l, hsp70.1, hsp70.2*, and *hsp70.3*), which exhibited low expression in all other cell clusters (Fig. 2C and Supplementary Fig. 8A, B).

To validate the expression and define the localization of this stressed *hsp70* + EC subset, we performed whole-mount fluorescence RNA in situ hybridization (FISH). FISH at 2 dpf revealed that *hsp70 *+ cells were most prominent within the CVP but also evident, to a lesser extent, in the DA, ISVs, DLAV and cerebral vessels of *ldlr*-/- zebrafish, while effectively absent in *ldlr* + / + embryos (Fig. 2D and Supplementary Fig. 8C). Time-series analysis demonstrated that endothelial *hsp70* upregulation in *ldlr*-/- embryos temporally coincided with abnormal CVP remodeling angiogenesis at 2 dpf (Fig. 2D and Supplementary Fig. 8D). Immunofluorescence staining supported these findings, showing enhanced vascular Hsp70 within a subset of ECs in *ldlr-/-* zebrafish at 2 dpf (Supplementary Fig. 8C). In line with the spatially distinct flow patterns observed, *hsp70* + ECs were predominantly located within the dCVP, correlating with increased dCVP loops and a wider CVP diameter (Fig. 2D and Supplementary Fig. 9), suggesting these acutely stressed ECs may alter CVP remodeling.

Comparative gene set enrichment analysis (GSEA) of the *ldlr*-/- specific *hsp70* + EC cluster indicated increased transcription of endoplasmic reticulum (ER) stress, incorrect protein folding, and lipid trafficking genes, while pathways related to translation, mitochondrial function and cytoskeletal organization were downregulated (Supplementary Fig. 10A). Significantly enriched cellular stress response genes in *hsp70* + ECs included *dnajb1b*, *ddit3*, *hspa5* and *atf4a* (Supplementary Fig. 10B). Further quantification of the most upregulated stress genes showed *ddit3* and *hspa5* were increased in *ldlr* homozygotes at 1 and 3 dpf, alongside *atf4a* and *dnajb1b* at 1 dpf (Fig. 2E). These findings indicate that *ldlr*-/- zebrafish exhibit a cellular stress response during embryonic and early larval development, with transient *hsp70* upregulation in a subset of stressed trunk endothelial cells.

### Flow drives endothelial *hsp70* and aberrant angiogenic remodeling in *ldlr*-/- zebrafish

**Fig. 3:**
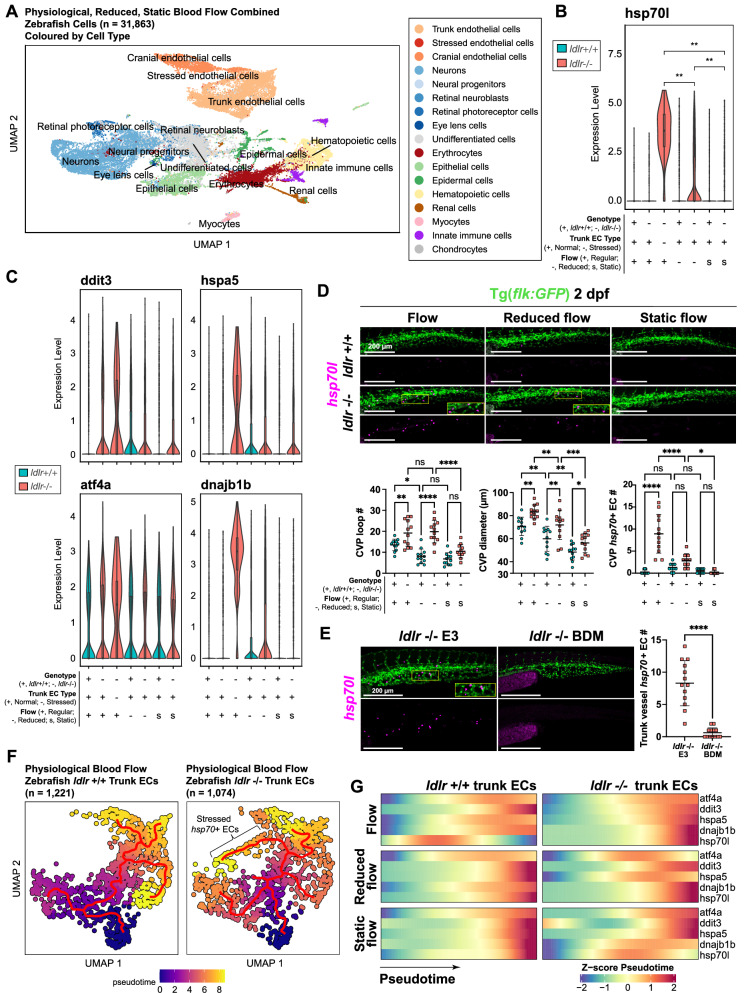
Endothelial *hsp70* upregulation and altered remodeling angiogenesis are mediated by blood flow in *ldlr*-/- embryos. **A** Combined cluster UMAP of 31,863 single cells showing annotated cell populations from embryonic *ldlr* + / + and *ldlr*-/- zebrafish under physiological, reduced (0.4 ng *tnnt2a* morpholino) and static (2 ng *tnnt2a* morpholino) blood flow conditions. Cells were obtained from pooled embryos (*n* = 200 zebrafish per group) processed in a single batch. **B** Violin plot of normalized *hsp70l* expression in trunk and stressed endothelial cells across genotypes and flow conditions. Each violin represents single-cell expression values, the central line indicates the median, and the shape represents the kernel distribution. Two-tailed Wilcoxon rank sum test and Bonferroni’s multiple comparison test used. ** *p* < 0.01. **C** Violin plots of normalized cell stress response expression (*ddit3*, *hspa5*, *atf4a* and *dnajb1b*) in trunk and stressed endothelial cells across genotypes and flow conditions. **D** Confocal microscopy of *hsp70l* fluorescence in situ RNA hybridization (FISH) stained trunk vasculature in *ldlr* + /+ and *ldlr*-/- zebrafish under physiological, reduced and static blood flow at 2 dpf. The inset shows CVP *hsp70 *+ endothelial cells adjacent to CVP loops (top). Caudal venous plexus (CVP) metrics: loop number, diameter (μm) and *hsp70*+ endothelial cell number in *ldlr* + /+ (*n* = 10–12) a*n*d *ldlr*-/- zebrafish (*n* = 10–12) across flow conditions. Flow *ldlr* + / + vs flow *ldlr*-/- (loop number: *p* = 0.0086, diameter: *p* = 0.0030, *hsp70 *+ number: *p* < 0.0001). Reduced flow *ldlr* + /+ vs reduced flow *ldlr*-/- (loop number: *p* < 0.0001, diameter: *p* = 0.0039, *hsp70 *+ number: *p* = 0.2918). Static flow *ldlr* + /+ vs static flow *ldlr*-/- (loop number: *p* = 0.1083, diameter: *p* = 0.0226, *hsp70 *+ number: *p* = 0.7410). Flow *ldlr* + / + vs reduced flow *ldlr* + / + (loop number: *p* = 0.0105, diameter: *p* = 0.0048, *hsp70*+ number: *p* = 0.4621). Reduced flow *ldlr* + /+ vs static flow *ldlr* + /+ (loop number: *p* = 0.7196, diameter: *p* = 0.0046, *hsp70*+ number: *p* = 0.6153). Flow *ldlr*-/- vs reduced flow *ldlr*-/- (loop number: *p* = 0.7196, diameter: *p* = 0.0002, *hsp70*+ number: *p* < 0.0001). Reduced flow *ldlr*-/- vs static flow *ldlr*-/- (loop number: *p* < 0.0001, diameter: *p* = 0.0046, *hsp70 *+ number: *p* = 0.0218). 3 independent experiments. Data are mean ± s.d. Two-way ANOVA and Holm-Sidak’s multiple comparison used (bottom). **E** Confocal microscopy of *hsp70l* FISH-stained trunk vasculature in *ldlr*-/- zebrafish exposed to E3 (control) medium or 10 mM BDM (1-2 dpf). Number of trunk vessel *hsp70 *+ endothelial cells in *ldlr*-/- zebrafish exposed to E3 control medium (*n* = 13) or 10 mM BDM (*n* = 13); *p* < 0.0001; 3 independe*n*t experiments. Data are mean ± s.d. Paired two-tailed *t* test was used (right). **F** Pseudotime trajectory UMAP of trunk endothelial cells in *ldlr* + /+ (left) and *ldlr*-/- (right) zebrafish under physiological blood flow. Trajectory and lineage path of stressed *hsp70 *+ endothelial cells is indicated in *ldlr*-/- zebrafish (absent in *ldlr* + /+ zebrafish) with flow. **G** Pseudotime z-score heatmaps of cell stress response genes (*atf4a*, *ddit3*, *hspa5*, *dnajb1b* and *hsp70l*) in *ldlr* + / + (left) and *ldlr*-/- (right) trunk endothelial cells under physiological (top), reduced (middle) and static (bottom) blood flow. Source data are provided as a Source Data file.

To investigate the role of blood flow in *ldlr*-/- vascular development, we modified zebrafish hemodynamics and examined the consequences on EC populations and angiogenesis phenotypes. Blood flow was reduced by dose-dependently blocking cardiac troponin T2 (*tnnt2a*) translation to impair heart contractility, generating embryos with static and reduced trunk vessel flow (Supplementary Fig. 11A, B, Supplementary Movies 4, 5, 6), followed by scRNA-seq as performed with physiological flow (Fig. 2). Transcriptional clustering identified all major cell types under static and reduced blood flow conditions in both *ldlr* + /+ and *ldlr*-/- embryos at 2 dpf. However, fewer neural cell sub-clusters were detected, and additional undifferentiated and epithelial cell clusters were present under static or reduced flow (Fig. 3A, Supplementary Figs. 12A, 13A). Unlike under physiological flow conditions where an additional stressed *hsp70*+ trunk EC subset was identified in *ldlr*-/- embryos (Fig. 2, Supplementary Fig. 10A), this population was absent with reduced or static flow conditions (Supplementary Figs. 12A, C, 13A, C). In *ldlr*-/- embryos, flow-inhibited groups showed lower trunk EC *hsp70l* expression compared to the stressed EC subset, while *hsp70l* levels remained relatively low across flow conditions in trunk ECs from *ldlr* + / + embryos, (Fig. 3B and Supplementary Fig. 14). Other identified cell stress genes (Fig. 2E) were variably expressed in trunk ECs across physiological, reduced, and static flow conditions in both *ldlr* + */+* and *ldlr-/-* embryos (Fig. 3C and Supplementary Figs. 12B, 13B), suggesting that ER stress pathways are upregulated independently of flow.

FISH staining under flow-inhibited conditions confirmed that CVP *hsp70* + ECs were significantly diminished with reduced and static flow in *ldlr* + */+* and *ldlr-/-* embryos (Fig. 3D). Notably, *ldlr*-/- embryos exposed to reduced flow displayed more CVP *hsp70* + ECs compared to *ldlr*-/- embryos under static flow, a pattern also reflected by scRNA-seq (Supplementary Fig. 14). To corroborate the role of blood flow in enhancing endothelial *hsp70*, we used the myosin ATPase inhibitor, 2,3-butanedione-monoxime (BDM) in *ldlr*-/- embryos to inhibit hemodynamics from 1 dpf (Supplementary Fig. 11D, Supplementary Movies 7, 8). Similar to *tnnt2a* inhibition, BDM treatment significantly reduced *hsp70* + EC quantity (Fig. 3E), supporting the flow-dependence of *hsp70* induction in *ldlr*-/- ECs.

We also performed vascular phenotyping of *ldlr* + /+ and *ldlr*-/- embryos with reduced or static blood flow to relate the scRNA-seq findings to specific angiogenic phenotypes. Both *ldlr* + /+ and *ldlr*-/- embryos under static flow failed to undergo vascular remodeling and displayed reduced CVP diameters with few CVP loops compared to zebrafish with flow (Fig. 3D and Supplementary Fig. 11C), consistent with previous findings^24^. Under reduced flow conditions, partial CVP remodeling occurred in both genotypes, but *ldlr*-/- embryos showed an increased number of CVP loops, which were larger, and a greater CVP diameter compared to their *ldlr* + /+ counterparts (Fig. 3D and Supplementary Fig. 11C). Together, these data suggest that blood flow contributes to reduced CVP remodeling angiogenesis in a dose-dependent manner in vivo in *ldlr*-/- animals.

To further elucidate the influence of blood flow on endothelial *hsp70* activity, cell stress signaling and developmental processes, we performed pseudotime trajectory analysis for all trunk EC populations (Fig. 3F and Supplementary Fig. 15). Endothelial cell trajectory analysis under physiological flow confirmed the *hsp70*+ subpopulation is derived from the main trunk EC population (Fig. 3F). Moreover, we found higher expression of ER stress response genes *atf4a*, *ddit3*, and *hspa5* prior to *hsp70l* and *dnajb1b* enrichment in *ldlr*-/- embryos under flow (Fig. 3G and Supplementary Fig. 15A–E), reinforcing coordinated upstream ER stress signaling. Pseudotime-specific coupling of cytoplasmic molecular chaperones *hsp70l* and *dnajb1b* was also detected in *ldlr*-/- embryos with reduced flow, while ER stress genes were variably expressed across pseudotime in zebrafish under reduced and static flow (Fig. 3G and Supplementary Fig. 15A–E). Additional trajectory pathway analysis indicated that nuclear division and cytoskeletal dysregulation coincided with blood flow-induced *hsp70* expansion in *ldlr*-/- ECs (Supplementary Fig. 15F, G), aligning with the altered vascular phenotyping (Fig. 1).

### Flow drives *HSP70* in human aortic ECs but other stress responses vary in flow-dependence

**Fig. 4:**
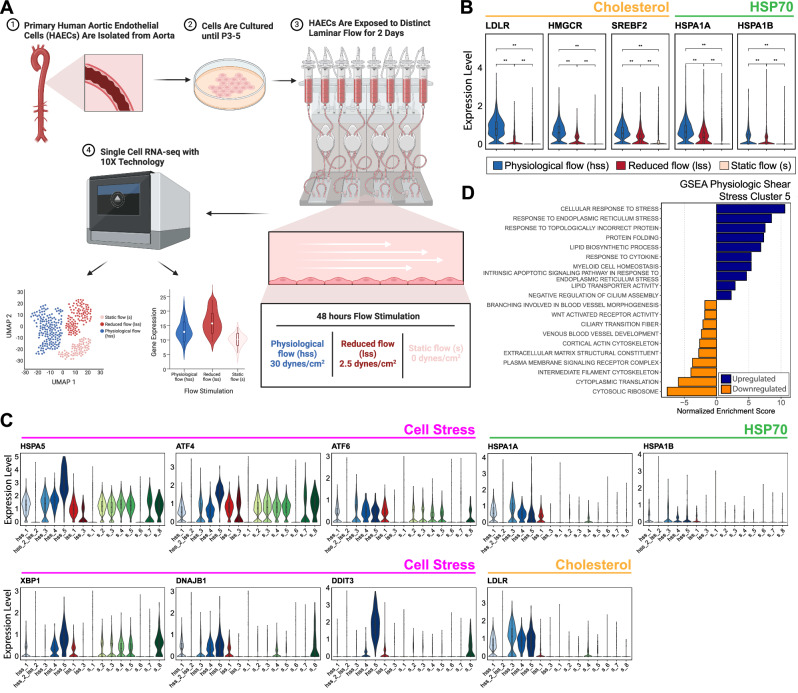
scRNA-sequencing of flow-modulated human aortic endothelial cells identifies preferential upregulation of *LDLR* and *HSP70* under physiological flow, with heterogenous activation of other cell stress pathways. **A** Schematic of human aortic endothelial cell (HAEC) culture and scRNA-seq workflow following exposure to physiological flow (30 dynes/cm^2^; high shear stress, hss), reduced flow (2.5 dynes/cm^2^; low shear stress, lss) or static (0 dynes/cm^2^; s) flow conditions. Created in BioRender. Salazar, A. (2026) https://BioRender.com/8cz4xxd. A total of 10,083 HAECs were processed in a single batch. **B** Violin plots of normalized *LDLR*, *HMGCR*, *SREBF2*, *HSPA1A* and *HSPA1B* expression across flow conditions. Each violin represents single-cell expression values, the central line indicates the median, and the shape represents the kernel distribution. Two-tailed Wilcoxon rank sum test and Bonferroni’s multiple comparison test used. ** *p* < 0.01. **C** Violin plots of normalized *LDLR*, *HSPA1A*, *HSPA1B*, *HSPA5*, *ATF4*, *ATF6*, *XBP1*, *DNAJB1* and *DDIT3* expression across HAEC clusters under physiological (hss), reduced (lss) and static (s) flow conditions. **D** Gene set enrichment analysis (GSEA) of HAEC physiological (hss) cluster 5 showing significantly upregulated and downregulated pathways.

We further investigated EC transcriptional responses to varying flow conditions by exposing human aortic ECs (HAECs) to physiological (high shear stress, hss), reduced (low shear stress, lss) or static (s) flow, and analyzing their expression profiles by scRNA-seq (Fig. 4A). While overall expression of *LDLR*, cholesterol synthesis genes (*HMGCR* and *SREBF2*) and human *HSP70* orthologs (*HSPA1A* and *HSPA1B)* showed significant upregulation under physiological flow compared with low shear stress and static conditions (Fig. 4B), there was evidence of heterogeneity across HAEC clusters. Grouped HAEC clustering identified eight static flow populations (s_1-8), four physiological flow populations (hss_1_3-5), two reduced flow populations (lss_1_3), and one joint physiological and reduced flow population (hss_2+lss_2) (Supplementary Fig. 16). GSEA demonstrated distinct transcriptional profiles across the clusters, with cytoskeletal, organelle and cell stress terms variably enriched (Supplementary Fig. 17A).

*LDLR* and *HSP70* were coordinately upregulated in all physiological flow clusters (hss_1_3-5) and within the transition zone of clusters hss_5 and lss_1 (Fig. 4C and Supplementary Fig. 17B). Other cell stress genes showed greater heterogeneity and were enriched across physiological, reduced and static flow clusters (Fig. 4C), a similar trend to transcriptional responses observed in flow-modulated zebrafish ECs (Fig. 3C). Among all HAEC subpopulations, physiological flow cluster hss_5 exhibited marked upregulation of cell stress response genes, including *DDIT3*, *ATF4, HSPA5* and *DNAJB1* (Fig. 4C, D), orthologs of genes enhanced in the *hsp70* + EC population of *ldlr-/-* zebrafish (Supplementary Fig. 10B). Cluster hss_5 also upregulated lipid trafficking, incorrect protein folding and pro-inflammatory pathways while downregulating cytoskeletal and translation processes (Fig. 4D), recapitulating the general profile of *hsp70*+ zebrafish ECs (Supplementary Fig. 10A). Furthermore, ciliogenesis-related genes were enriched in reduced flow clusters (Supplementary Fig. 17C) and cilia-associated pathways were downregulated in cluster hss_5 (Fig. 4D).

In comparison to the zebrafish vascular endothelium, *HSP70* upregulation in HAECs occurred across all flow-exposed endothelial subsets and did not clearly resolve into a distinct subpopulation, as observed in *Idlr*-/- embryos. This difference likely reflects physiological and experimental distinctions between the models, including venous plexus flow in vivo versus aortic endothelial flow in vitro, as well as deeper cellular coverage for HAECs. Nevertheless, flow-dependent co-upregulation of the cytoplasmic molecular chaperones, *HSP70* and *DNAJB1*, was observed in HAECs (Fig. 4C), mirroring the coupling found in *ldlr*-/- zebrafish trunk ECs (Fig. 3C and Supplementary Fig. 10B), suggesting partially analogous endothelial responses to flow across the models.

Our flow-mediated HAEC expression profiling therefore confirms a discrete subset of ECs exposed to physiological flow that upregulate cell stress pathways, *HSP70* and lipid trafficking genes, while downregulating ciliogenesis genes. Integration of our HAEC dataset with publicly available flow-modulated scRNA-seq datasets reiterated this shear stress-dependent heterogeneity among human EC clusters (Supplementary Fig. 18). Collectively, these findings establish heterogeneity in flow-modulated transcription of cell stress responses across HAECs, while *HSP70* is preferentially upregulated by physiological flow.

### Hsp70 upregulation inhibits vascular apoptosis, remodeling angiogenesis and ciliogenesis in zebrafish

**Fig. 5:**
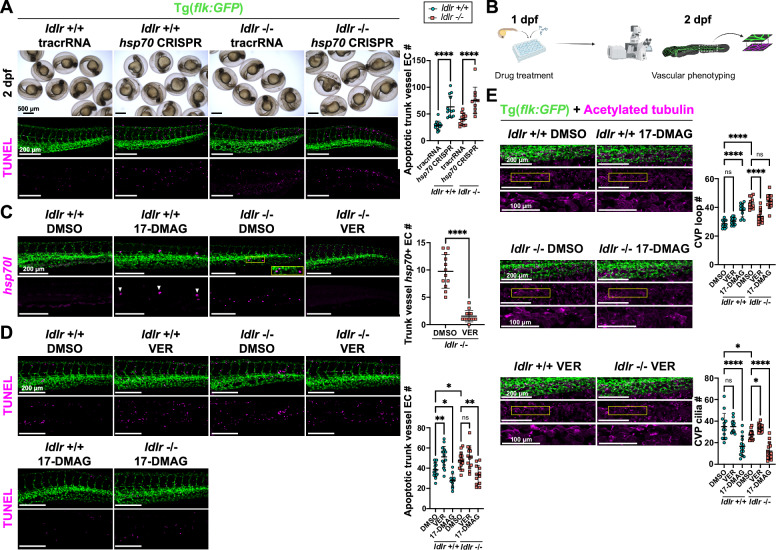
Genetic and pharmacological modulation indicates *hsp70* upregulation inhibits vascular apoptosis, remodeling angiogenesis and ciliogenesis during embryonic zebrafish development. **A** Brightfield microscopy of *ldlr* + /+ and *ldlr*-/- zebrafish following acute *hsp70* CRISPR knockout or control tracrRNA injection at 2 dpf (top, left). Confocal microscopy of TUNEL-stained trunk vasculature is shown (bottom, left). Number of apoptotic endothelial cells in trunk vasculature in *ldlr* + / + tracRNA (*n* = 13) vs *ldlr* + / + *hsp70* gRNA (*n* = 13); *p* < 0.0001; and *ldlr*-/- tracRNA (*n* = 13) vs *ldlr*-/- *hsp70* gRNA (*n* = 10); *p* < 0.0001; 3 independent experiments. Data are mean ± s.d. One-way ANOVA and Holm-Sidak’s multiple comparison used (right). **B** Schematic of zebrafish drug treatment (1-2 dpf) followed by vascular staining and phenotyping. Created in BioRender. Kaveh, A. (2026) https://BioRender.com/pwxcj5h. **C** Confocal microscopy of *hsp70l* fluorescence in situ RNA hybridization-stained Tg(*flk:GFP*) *ldlr* + / + zebrafish treated with 50 μM 17-DMAG and *ldlr*-/- zebrafish treated with 50 μM VER-155008 at 2 dpf. Arrowheads indicate neuromast *hsp70* expression. Inset shows caudal venous plexus (CVP) *hsp70 *+ endothelial cells adjacent to CVP loops (left). Total number of trunk vessel *hsp70 *+ endothelial cells in *ldlr*-/- zebrafish treated with DMSO (*n* = 12) or 50 μM VER-155008 treated (*n* = 13); *p* < 0.0001; 3 i*n*dependent experiments. Data are mean ± s.d. Paired two-tailed *t* test was used (right). **D** Confocal microscopy of TUNEL-stained trunk vasculature in *ldlr* + / + and *ldlr*-/- zebrafish treated with 50 μM VER-155008, and 50 μM 17-DMAG or DMSO (left). Total number of trunk vessel apoptotic endothelial cells in zebrafish treated with 50 μM VER-155008 (*n* = 13-16), 50 μM 17-DMAG (*n* = 12) or DMSO (*n* = 15). *Ldlr* + /+ DMSO vs *ldlr*-/- DMSO (*p* = 0.0469)*; ldlr* + / + DMSO vs *ldlr* + / + VER (*p* = 0.0018); *ldlr* + /+ DMSO vs *ldlr* + / + 17-DMAG (*p* = 0.0136); *ldlr*-/- DMSO vs *ldlr*-/- VER (*p* = 0.1680); *ldlr*-/- DMSO vs *ldlr*-/- 17-DMAG (*p* = 0.0029); 3 independent experiments. Data are mean ± s.d. Two-way ANOVA and Holm-Sidak’s multiple comparison used (right). **E** Confocal microscopy of acetylated tubulin-stained trunk vasculature in *ldlr* + / + and *ldlr*-/- zebrafish treated with 50 μM 17-DMAG, 50 μM VER-155008 or DMSO at 2 dpf. Insets show CVP cilia (left). CVP loop number in zebrafish treated with 50 μM VER-155008 (*n* = 12-13), 50 μM 17-DMAG (*n* = 12-13) or DMSO (*n* = 11-12) (top, right). CVP cilia number in zebrafish treated with 50 μM VER-155008 (*n* = 14), 50 μM 17-DMAG (*n* = 14) or DMSO (*n* = 13-14) (bottom, right). *Ldlr* + /+ DMSO vs *ldlr*-/- DMSO (loop number: *p* < 0.0001 and cilia number: *p* = 0.0385)*; ldlr* + /+ DMSO vs *ldlr* + /+ VER (loo*p* number: *p* = 0.4090 and cilia number: *p* = 0.9501); *ldlr* + /+ DMSO vs *ldlr* + /+ 17-DMAG (loop number: *p* < 0.0001 and cilia number: *p* < 0.0001); *ldlr*-/- DMSO vs *ldlr*-/- VER (loop number: *p* < 0.0001 and cilia number: *p* = 0.0475); *ldlr*-/- DMSO vs *ldlr*-/- 17-DMAG (loop number: *p* = 0.6249 and cilia number: *p* < 0.0001); 3 independent experiments. Data are mean ± s.d. Two-way ANOVA and Holm-Sidak’s multiple comparison used. Source data are provided as a Source Data file.

To investigate the role of *hsp70* in embryonic *ldlr*-/- zebrafish, we first tested its loss of function during development using acute CRISPR/Cas9 mutagenesis. Amplicon sequencing confirmed efficient *hsp70* mutagenesis in F0 embryos, and *hsp70* crispants displayed significant developmental abnormalities, including delayed embryogenesis, body axis curvature, edema, and increased mortality, accompanied by increased vascular apoptosis in both *ldlr* + /+ and *ldlr*-/- zebrafish (Fig. 5A and Supplementary Fig. 19A).

To circumvent these broad early developmental defects, we temporally inhibited Hsp70 during a later embryologic window using the pharmacological inhibitor VER-155008 (VER)^25^ from 1 dpf to 2 dpf (Fig. 5B). VER treatment significantly reduced the number of *hsp70* + ECs in the trunk vessels of *ldlr-/-* embryos, without affecting overall development (Fig. 5C and Supplementary Fig. 19B). In addition, VER increased endothelial apoptosis in *ldlr* + /+ embryos; however, apoptosis was not further elevated in *ldlr-/-* embryos, which already exhibit markedly increased baseline apoptosis (Fig. 5D). This observed ceiling effect may explain why VER-mediated apoptosis is detected in *ldlr* + / + but not *ldlr*-/- trunk vessels.

To test the consequences of Hsp70 upregulation, we used the pharmacological activator, 17-DMAG^26^. 17-DMAG treatment robustly increased *hsp70* expression in lateral line neuromast cells (Fig. 5C), suggesting Hsp70 may preferentially mark stressed cells in mechanosensory tissues. In contrast to VER, 17-DMAG significantly reduced trunk vessel apoptosis in both *ldlr* + /+ and *ldlr-/-* embryos (Fig. 5D), supporting a broadly anti-apoptotic role for Hsp70.

We then examined how 17-DMAG and VER treatments affect other characterized vascular abnormalities. 17-DMAG reduced apoB accumulation in the CVP of *ldlr-/-* embryos, whereas VER showed no clear change (Supplementary Fig. 20A), suggesting that Hsp70 upregulation may promote vascular apoB clearance in homozygotes. In addition to reducing vascular apoptosis and excess apoB levels, 17-DMAG markedly disrupted CVP remodeling in *ldlr* + / + embryos, increasing the number of CVP loops, which remained significantly elevated in *ldlr*-/- embryos (Fig. 5E).

Since *ldlr-/-* zebrafish display impaired CVP ciliogenesis and defective neuromast development (Fig. 1), we next assessed how Hsp70 modulation influences these mechanosensory phenotypes in both genotypes. In *ldlr* + / + embryos, 17-DMAG significantly reduced CVP ciliation and neuromast cell numbers (Fig. 5E and Supplementary Fig. 21), whereas VER had no effect on CVP patterning (Fig. 5E). In *ldlr-/-* embryos, 17-DMAG further exacerbated pre-existing CVP ciliogenesis defects (Fig. 5E). In contrast, VER treatment rescued multiple phenotypes in *ldlr-/-* embryos, increasing CVP cilia number and neuromast cells while reducing CVP diameter and CVP loop formation (Fig. 5E and Supplementary Fig. 20B, 21). These findings suggest that Hsp70 modulation differentially affects vascular apoptosis and remodeling/ciliogenesis, with Hsp70 upregulation disrupting CVP ciliogenesis and remodeling angiogenesis.

Given vascular apoptosis and ER stress are elevated from 1 dpf in *ldlr*-/- embryos (Figs. 1B, 2E) and p38 Mapk signaling facilitates early cell stress responses^27^, we inhibited p38 Mapk to examine its effects on endothelial *hsp70* expression and downstream mutant phenotypes. Inhibitor of p38 Mapk, SB-203580^28^, reduced trunk vessel *hsp70* + ECs, accelerated CVP remodeling angiogenesis and recovered neuromast development in *ldlr*-/- embryos (Supplementary Fig. 22A, B). Together, these results indicate that cell stress-induced *hsp70* upregulation suppresses vascular apoptosis while concurrently disrupting endothelial ciliogenesis and impairing remodeling angiogenesis, highlighting a multifaceted and context-dependent developmental role.

### *Ldlr* loss impairs regenerative angiogenesis and enhances inflammation in zebrafish

**Fig. 6:**
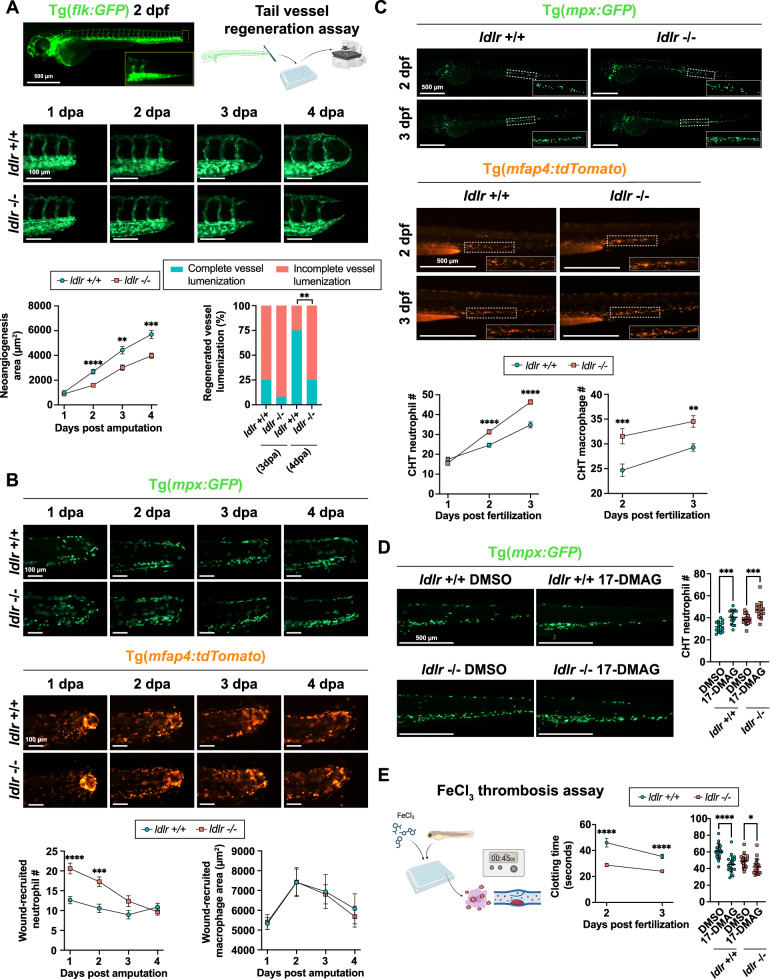
*Ldlr*-/- zebrafish display reduced regenerative angiogenesis, enhanced innate immune cell expansion and increased thrombogenicity during development. **A** Epifluorescence microscopy of Tg(*flk:GFP*) zebrafish at 2 dpf following tail vessel amputation. Inset shows the two most posterior intersegmental vessels resected (top, left). Schematic of tail vessel regeneration assay. Created in BioRender. Kaveh, A. (2026) https://BioRender.com/gh8cub2 (top, right). Tail vessel repair/regeneration in *ldlr* + /+ and *ldlr*-/- zebrafish from 1–4 days post amputation (dpa, middle). Quantification of neoangiogenesis area (μm^2^) in *ldlr* + /+ (*n* = 23-24) and *ldlr*-/- (*n* = 22-24) zebrafish at 1 dpa (*p* = 0.4308), 2 dpa (*p* < 0.0001), 3 dpa (*p* = 0.0011), 4 dpa (*p* = 0.0002); 4 independent experiments. Data are mean ± s.e.m. Two-way ANOVA and Holm-Sidak’s multiple comparison test used (bottom, left). Regenerated vessel lumenization (% with patent blood flow) in *ldlr* + /+ and *ldlr*-/- zebrafish at 3 dpa (*p* = 0.2448) and 4 dpa (*p* = 0.0012). Fisher’s exact (two-sided) test was (bottom, right). **B** Epifluorescence microscopy of neutrophil (Tg(*mpx:GFP*), top) and macrophage (Tg(*mfap4:tdTomato*), middle) wound recruitment during tail vessel repair/regeneration in *ldlr* + /+ and *ldlr*-/- zebrafish from 1–4 dpa. Wound-recruited neutrophil numbers (bottom, left) and macrophage area (μm^2^, bottom right) in *ldlr* + /+ (*n* = 18–20) a*n*d *ldlr*-/- (*n* = 17–20) zebrafish at 1 dpa (neutrophil: *p* < 0.0001 and macrophage: *p* = 0.9998), 2 dpa (neutrophil: *p* = 0.0006 and macrophage: *p* > 0.9999), 3 dpa (neutrophil: *p* = 0.1111 and macrophage: *p* = 0.9998), 4 dpa (neutrophil: *p* = 0.3521 and macrophage: *p* = 0.9998); 3 independent experiments. Data are mean ± s.e.m. Two-way ANOVA and Holm-Sidak’s multiple comparison test used. (C) Epifluorescence microscopy of neutrophils (top) and macrophages (middle) in the caudal hematopoietic tissue (CHT, inset indicates the CHT) of zebrafish at 2 dpf and 3 dpf. CHT neutrophil numbers in *ldlr* + /+ (*n* = 21–29) and *ldlr*-/- (*n* = 21–30) zebrafish at 1 dpf (*p* = 0.2090), 2 dpf (*p* < 0.0001) and 3 dpf (*p* < 0.0001); 3 independent experiments (bottom, left). CHT macrophage numbers in *ldlr* + /+ (*n* = 13–15) and *ldlr*-/- (*n* = 13–15) zebrafish at 2 dpf (*p* = 0.0005) and 3 dpf (*p* = 0.0020); 2 independent experiments (bottom, right). Data are mean ± s.e.m. Two-way ANOVA and Holm-Sidak’s multiple comparison test used. (D) Epifluorescence microscopy of CHT neutrophils in *ldlr* + / + and *ldlr*-/- zebrafish following treatment with 50 μM 17-DMAG or DMSO (left). CHT neutrophil numbers in zebrafish treated with 50 μM 17-DMAG (*n* = 15) or DMSO (*n* = 15). *Ldlr* + /+ DMSO vs *ldlr* + / + 17-DMAG (*p* = 0.0005) a*n*d *ldlr*-/- DMSO vs *ldlr*-/- 17-DMAG (*p* = 0.0005); 3 independent experiments. Data are mean ± s.d. One-way ANOVA and Holm-Sidak’s multiple comparison used(right). (E) Schematic of FeCl_3_ thrombosis assay workflow. Created in BioRender. Kaveh, A. (2026) https://BioRender.com/zgdmudi (left). Time to thrombosis (clotting time, seconds) following FeCl_3_ exposure in *ldlr* + /+ (*n* = 22-28) and *ldlr*-/- (*n* = 22–29) zebrafish at 2 dpf (*p* < 0.0001) and 3 dpf (*p* < 0.0001); 3 independent experiments. Data are mean ± s.e.m. One-way ANOVA and Holm-Sidak’s multiple comparison test used (middle). Thrombosis time in *ldlr* + / + and *ldlr*-/- zebrafish treated with 50 μM 17-DMAG (*n* = 18–20) or DMSO (*n* = 19–20). *Ldlr* + /+ DMSO vs *ldlr* + / + 17-DMAG (*p* < 0.0001) a*n*d *ldlr*-/- DMSO vs *ldlr*-/− 17-DMAG (*p* = 0.0246); 3 independent ex*p*eriments. Data are mean ± s.d. One-way ANOVA and Holm-Sidak’s multiple comparison used (right). Source data are provided as a Source Data file.

We next assessed angiogenesis following injury in developing *ldlr*-/- zebrafish using a multi-vessel tail fin amputation paradigm. Serial live imaging of the tail fin vasculature up to 4 days post amputation (dpa) revealed reduced sprouting regenerative angiogenesis in *ldlr*-/- larvae compared to *ldlr* + /+ controls (Fig. 6A). At 2 dpa, 3 dpa and 4 dpa, *ldlr* + / + larvae exhibited greater neoangiogenic sprouting, with dorsal and ventral vascular sprouts migrating medially and connecting by 4 dpa (Fig. 6A). While the majority of *ldlr* + / + zebrafish (75%) showed regenerated and lumenized vessels with patent blood flow at 4 dpa, this was only achieved in 25% of *ldlr*-/- zebrafish (Fig. 6A, Supplementary Movies 9, 10).

We hypothesized that differences in inflammatory responses underlie the impaired vascular regeneration in *ldlr*-/- larvae. Therefore, we performed live imaging of neutrophil and macrophage recruitment following zebrafish wounding^29,30^. Neutrophil recruitment at the injury site was significantly augmented at 1 dpa and 2 dpa in *ldlr*-/- zebrafish, coinciding with impaired regenerative angiogenesis, and returned to *ldlr* + /+ levels by 4 dpa (Fig. 6B). Macrophage wound recruitment, however, was comparable between *ldlr* + /+ and *ldlr*-/- larvae across all timepoints (Fig. 6B).

To better understand the sustained neutrophilic inflammation following injury, we characterized immune cell and hematopoietic stem cell (HSC) development in *ldlr*-/- zebrafish by examining the caudal hematopoietic tissue (CHT). Live imaging showed increased neutrophil and macrophage numbers in the CHT of *ldlr*-/- zebrafish at 2 dpf and 3 dpf (Fig. 6C), which was corroborated with histological staining (Supplementary Fig. 23). HSC numbers were unchanged in the CHT of *ldlr*-/- embryos (Supplementary Fig. 24A), suggesting myeloid cell differentiation/expansion is preferentially enhanced during embryonic *ldlr*-/- development. Pharmacological Hsp70 inhibition with VER did not affect CHT myeloid cell numbers in *ldlr*-/- zebrafish (Supplementary Fig. 24B, C), while Hsp70 upregulation using 17-DMAG enhanced CHT neutrophils in both *ldlr* + /+ and *ldlr*-/- embryos, without affecting CHT macrophage or total HSC numbers (Fig. 6D and Supplementary Fig. 24A, C).

**Fig. 7:**
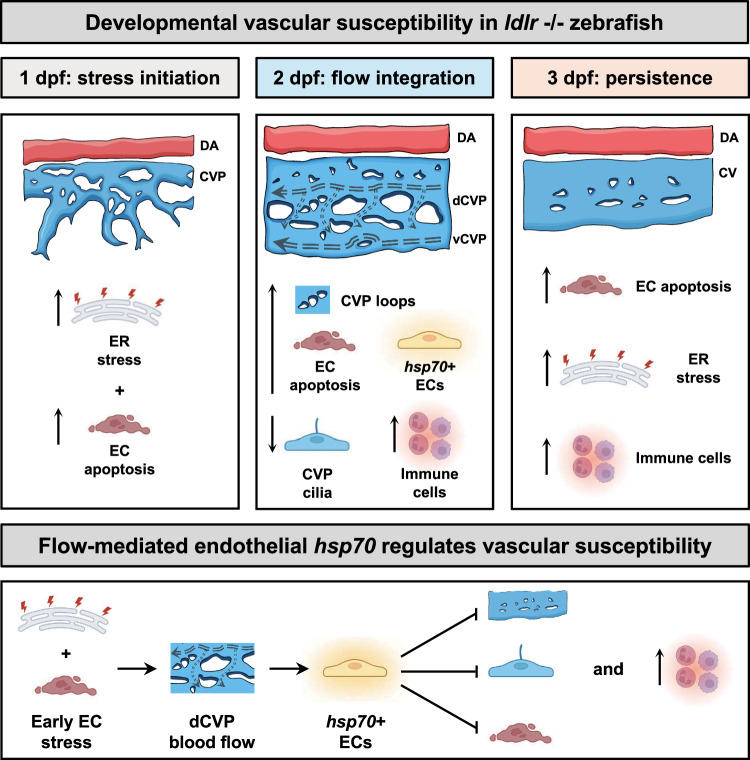
Schematic model of developmental vascular susceptibility in *ldlr*-/- zebrafish. At 1 dpf (stress initiation), *ldlr*-/- embryos exhibit elevated endoplasmic reticulum (ER) stress signaling accompanied by increased endothelial apoptosis in trunk vessels (top, left). At 2 dpf (flow integration), abnormal dorsal caudal venous plexus (CVP) angiogenic remodeling is observed, spatially associated with the emergence of flow-dependent *hsp70*+ endothelial cells (ECs). This stage is accompanied by sustained vascular apoptosis, increased CVP loop formation, reduced CVP ciliogenesis and enhanced expansion of innate immune cells within the caudal hematopoietic tissue. Dashed arrows indicate CVP blood flow direction and relative magnitude (top, middle). At 3 dpf (persistence), CVP remodeling remains partially disrupted, while vascular apoptosis, ER stress signaling and immune cell expansion persist (top, right). This model proposes that early endothelial stress converges with physiological blood flow through a structurally altered CVP to drive *hsp70* upregulation and modulate vascular susceptibility. The emergence of *hsp70* + ECs promotes endothelial survival while inhibiting endothelial ciliogenesis and angiogenic remodeling (bottom). Partly created in BioRender. Kaveh, A. (2026) https://BioRender.com/xft5lm8. DA, dorsal aorta; dCVP, dorsal caudal venous plexus; vCVP, ventral caudal venous plexus; CV, caudal vein.

As dysfunctional endothelial cells and neutrophils regulate blood clotting via pro-inflammatory and coagulation factors, we measured time to thrombosis following FeCl_3_ exposure, a clotting agent that causes endothelial oxidation through free radical generation^31^. Evaluation of reactive oxygen species indicated elevated baseline oxidative stress in *ldlr*-/- zebrafish, which was exacerbated at the tail fin following injury (Supplementary Fig. 25A, B). *Ldlr*-/- zebrafish exhibited a pro-thrombotic phenotype, with significantly reduced clotting times compared to *ldlr* + /+ zebrafish at 2 dpf and 3 dpf (Fig. 6E). While VER did not alter thrombosis times (Supplementary Fig. 25C), 17-DMAG treatment further shortened time to clotting in both *ldlr* + /+ and *ldlr*-/- zebrafish (Fig. 6E). Our findings therefore demonstrate that neutrophilia and thrombogenicity are augmented in *ldlr-/-* zebrafish, alongside abnormalities in flow-mediated angiogenic remodeling (Fig. 7).

## Discussion

While atherosclerosis has been extensively studied, the developmental origins of the disease remain unclear. Using *ldlr* knockout zebrafish, we reveal primordial abnormalities that give rise to and shape early endothelial cell dysfunction. By combining flow modulation, single-cell transcriptomics and in vivo imaging, we identified a hemodynamically-induced stressed endothelial cell subset in *ldlr* mutant animals. Physiological vascular stress arising during development is associated with multiple cellular abnormalities, including striking upregulation of endothelial *hsp70* in the stressed EC subset, slowing of angiogenic remodeling and defective responses to vascular injury. In parallel, we observe a dysregulated inflammatory cell compartment with consequent functional abnormalities of injury resolution and thrombogenicity. Together, these insights suggest that convergent impairments of endothelial and inflammatory responses to flow and injury render the vascular system in *ldlr*-/- animals vulnerable from embryonic development onwards (Fig. 7).

Loss of LDLR function produces a consistent phenotype in vertebrates, characterized by elevated LDL and vascular lipid deposition. Larval *ldlr* knockout zebrafish recapitulate these hallmarks^21^, although species differ in lipoprotein handling and lesion complexity^32–34^; Ldlr-deficient mice require additional genetic manipulation and dietary challenge to achieve human-like LDL profiles and typically develop fewer complex lesions, whereas rabbits and pigs form plaques that more closely resemble human coronary disease^33,35^. In larval *ldlr* knockout zebrafish, lipid accumulation occurs in the trunk vessels such as the dorsal aorta and caudal vein, which is exacerbated by high cholesterol feeding^21^. The developmental biology of atherosclerosis in FH models, however, remains poorly understood, though disease phenotypes arise in children with homozygous FH^36^. Although most non-genetic forms of atherosclerosis manifest in mid- to late life, longitudinal studies show that early life risk factors predict subclinical atherosclerosis and adult ASCVD, with markedly accelerated trajectories in FH^16,37^. Thus, mapping vascular phenotypes in a developmental model of FH may reveal how early endothelial perturbations influence subclinical atherosclerosis progression and provide insights for primary prevention in predisposed individuals^17^. While larger mammalian models and clinical studies remain indispensable for assessing atherosclerotic lesions, *ldlr* knockout zebrafish enable in vivo dissection of developmental, cellular and molecular mechanisms that establish vascular susceptibility to disease.

Beginning at one day post-fertilization, *ldlr* knockout zebrafish exhibited elevated ER stress marker expression and enhanced vascular apoptosis, features of ASCVD^38,39^. Vascular phenotyping revealed that remodeling of the venous plexus was aberrant in *ldlr* homozygote embryos, accompanied by a reduced number and length of endothelial cilia. While primary endothelial cilia are involved in vascular sprouting and subsequent remodeling through pruning^40,41^, *ldlr* knockout zebrafish displayed dysregulated ciliogenesis during the later remodeling angiogenesis phase, concurrent with defects in neuromast hair cell development. Consistent with these observations, ciliopathy models show pronounced angiogenic and vascular integrity defects^40–43^, underscoring the importance of cilia in maintaining developmental signaling and vascular health. Compared with other trunk vessels, the zebrafish CVP is densely ciliated and experiences lower blood flow velocity and wall shear stress^20,44^, conditions that may heighten endothelial susceptibility to cellular stress. Flow analyses found complex abnormalities of dorsal venous plexus flow in *ldlr* knockout embryos, the main capillary showing altered patterning angiogenesis. These abnormalities in developmental angiogenic remodeling may parallel clinical observations linking disturbed hemodynamics to atherosclerosis-susceptible vascular sites^45^. Together, our findings also raise the possibility of occult microvascular or venous defects in primary forms of atherosclerosis, though evidence of such is currently lacking in humans.

A developmentally distinct subset of stressed endothelial cells, marked by substantial *hsp70* overexpression, localized to geometrically complex vascular regions in *ldlr* mutants that experience disturbed flow and aberrant angiogenic remodeling. Although Hsp70 has not been implicated in LDLR-deficient models, its acute induction converged with progressive ER stress activation (via *ddit3* and *hspa5*) and endothelial apoptosis, reflecting a transient attempt to resolve cellular stress. Previous studies have shown that low or disturbed shear stress flow upregulates endothelial ER stress and UPR genes, including *HSPA5*^46,47^. In our flow-modified human endothelial cell data, *HSPA5* expression was heterogeneous, with enrichment in static, low and high-shear-exposed HAECs. While ER stress pathway activation was detected across all flow conditions, the flow modulation experiments suggest hemodynamic upregulation of *hsp70* in subsets of both zebrafish and human endothelial cells. Despite species, vessel type and developmental differences, endothelial *HSP70* upregulation was consistently coupled with *DNAJB1* expression, another cytoplasmic molecular chaperone. Dnajb1 (a Hsp40 family member) is a co-chaperone of Hsp70 that enhances its ability to buffer cellular stress^48,49^, suggesting that their adaptive synergy is conserved in the flow-responsive endothelium.

Hsp70 is recognized for its key roles in maintaining cellular proteostasis^50^. However, to our knowledge, it has not been previously reported as inducible by physiological flow in vivo. Our *ldlr* knockout zebrafish model suggests that endothelial *hsp70* is activated by early cellular stress, via p38 Mapk signaling, and its upregulation is mediated by graded physiological flow. While *hsp70* upregulation protected against endothelial apoptosis, it impaired CVP ciliogenesis and remodeling angiogenesis (Fig. 7). These findings indicate that flow-induced molecular chaperone activity mediates a local trade-off between endothelial survival and structural remodeling. By buffering intracellular stress, *hsp70* + ECs may inadvertently delay dorsal venous plexus pruning by suppressing kinocilia formation and cilia-dependent signaling^41^. Although the mechanosensory basis of *hsp70* flow inducibility remains unclear in vivo, we posit that altered membrane composition due to endocytic dysfunction could sensitize the endoplasmic reticulum to flow-induced stress, thereby engaging heat shock transcription factor machinery^51^. Further exploration of the interactions among canonical mechanosensors such as cilia, caveolae, and junctional complexes^52^ – together with Ldlr and Hsp70, may shed light on how endothelial cells integrate mechanical and stress signals under physiological and pathological flow.

In other contexts, Hsp70 has been implicated in regulating subcellular sphingolipid metabolism in models of Niemann-Pick disease^53,54^. Hsp70 has also been reported to facilitate cholesterol efflux from lipid-laden cells^55^ and to be secreted extracellularly following high-fat dieting^56^, suggesting a role in lipid trafficking under conditions of intracellular lipid imbalance. In our zebrafish dataset, lipid pathway genes were minimally expressed at the single-cell level; however, apoB, the principal apolipoprotein of LDL, was transiently elevated in the venous plexus of *ldlr* mutants. This increase normalized by 3 dpf, as normalization of endothelial *hsp70* expression with angiogenic remodeling is being completed. Recent live imaging studies showed that apoB-containing lipoproteins have a shorter half-life at 2 dpf compared to 5 dpf, and that *ldlr* homozygotes exhibit elevated systemic apoB levels from 4 dpf onward^32,34^. Furthermore, Hsp70 can promote apoB degradation^57^. Combined, these observations suggest that the transient developmental vascular phenotypes in *ldlr*-deficient endothelial cells may partly reflect Hsp70 functions in handling lipid trafficking and metabolism^53–55^ - processes involved in cellular stress adaptation^58^. Further investigation of lipid and membrane trafficking in this context could reveal additional mechanisms by which stress-induced molecular chaperones influence vascular homeostasis and disease susceptibility.

The dorsal caudal venous plexus, where *ldlr* mutant developmental phenotypes were most pronounced, also serves as the primary hematopoietic site during zebrafish embryogenesis, providing a vascular niche for hematopoietic stem and immune cells^59,60^. We observed a selective expansion of innate immune cells, but not hematopoietic stem cells, overlapping with vascular abnormalities in *ldlr* knockout zebrafish (Fig. 7). In examining features of ASCVD during early development, we found that loss of *ldlr* function impaired vascular regeneration, heightened neutrophilic inflammation, elevated reactive oxygen species, and accelerated thrombosis. Notably, neutrophilia and thrombogenicity were exacerbated by Hsp70 activation and pro-inflammatory gene signatures were enriched in *hsp70*-overexpressing endothelial cells, suggesting possible endothelial-neutrophil crosstalk. While intracellular Hsp70 is typically considered vascular-protective, extracellular Hsp70 can activate innate immune responses^61,62^. Thus, endothelial overexpression of Hsp70 may promote secretion of the protein to modify the local inflammatory milieu^63^, though effects on atherosclerosis vary by cell type and disease context^61^. Together, these findings suggest a feedforward loop wherein flow-responsive endothelial abnormalities and inflammatory signaling amplify developmental vascular susceptibility (Fig. 7). The results also emphasize the potential importance of early modulation of inflammatory pathways or prevention of vascular injury in LDLR-deficient individuals.

In summary, this study establishes a developmental model of atherosclerosis by integrating embryonic and larval phenotyping of *ldlr* knockout zebrafish with blood flow modulation. We identified a transitional mechanoresponsive endothelial subpopulation that regulates key atherosclerosis traits, detailing a critical early temporal window during which endothelial dysfunction and inflammation arise and converge. These findings provide a framework for investigating co-regulatory cellular mechanisms driving vascular susceptibility and may guide preventive strategies for treating individuals prone to accelerated atherosclerosis and ASCVD.

## Methods

### Zebrafish maintenance and use

All experiments involving zebrafish were conducted in compliance with animal protocols approved by the Institutional Animal Care and Use Committee of Brigham and Women’s Hospital at Harvard Medical School. Zebrafish were maintained in a dedicated aquatics facility at 28 °C with a circulating and filtered system, as per standard operating procedures. Experiments were performed on embryos or larvae aged between 1 dpf and 6 dpf, which were incubated at 28 °C in standard embryo (E3) medium. The sex of zebrafish is not specified at embryonic or larval stages and therefore was not considered in experiments.

Homozygous *ldlra*^*sd52/sd52*^ zebrafish, which are viable to adulthood^21^, were crossed with the following transgenic reporter lines to generate heterozygote mutants: Tg(*flk:eGFP*)^*s843*^^64^, Tg(*gata1a:dsRed*)^*sd2*^^65^, Tg(*mpx:GFP*)^*i114*^^66^, Tg(*mfap4:tdTomato)*^*xt12*^^67^ and Tg(*cd41:eGFP*)^*la2*^^68^. Initial imaging experiments involved incrossing *ldlr* heterozygous mutants, with embryos/larvae subsequently genotyped in a blinded manner to identify wild-type (*ldlr* + /+), heterozygous (*ldlr* + /-) or homozygous (*ldlr*-/-) individuals. Following experiments were performed using embryos and larvae from incrosses of confirmed *ldlr* + /+ and *ldlr*-/- siblings. For genotyping, genomic DNA was extracted using 50 μM NaOH (95 °C for 15 minutes), followed by PCR amplification with Quick-Load Taq 2x master mix (New England Biolabs). Sanger sequencing was performed using the following primers:

*ldlra* Fw primer: TATGAACAGCCTTGCCAACA

*ldlra* Rv primer: GCTGAAAATCGGGAGAAGAG

Transgenic adult zebrafish were maintained on a *casper* or *nacre* background^69^ to enhance imaging clarity or treated with 0.003% phenylthiourea (Sigma) in E3 medium from 8 hours post fertilization to inhibit pigment formation.

### Imaging embryonic and larval zebrafish

Prior to live imaging, zebrafish embryos and larvae were anesthetized with 40 μg/mL tricaine methanesulphonate (FisherScientific) in E3 medium until immobile and imaged at room temperature using confocal or epifluorescence microscopy. Confocal images were acquired using an upright Olympus FV1200 microscope equipped with an Argon 458/477/488/514 nm laser and a Red HeNe 633 nm laser. For confocal imaging, zebrafish were mounted in 35 mm glass-bottom dishes (MatTek) using 0.75% ultrapure low-melting-point agarose (Invitrogen) dissolved in E3 medium. Confocal images were acquired using a 10x objective with 20-25x magnification.

Brightfield and epifluorescence images were acquired using an EVOS Auto2 system (ThermoFisher) with GFP, RFP and Texas Red filters. For EVOS live imaging, embryos and larvae were laterally mounted on Superfrost microscope glass slides (ThermoFisher) and imaged using 4x, 10x, or 20x objectives.

### Whole-mount zebrafish immunofluorescence staining

Zebrafish embryos and larvae were fixed overnight at 4 °C in the dark in 4% paraformaldehyde (PFA) in phosphate-buffered saline (PBS) [PFA-PBS]. Following fixation, samples were rinsed four times with PBS containing 0.1% Tween20 (PBST). Zebrafish were then washed twice in blocking buffer (5 mg/ml bovine serum albumin [BSA] and 5% goat serum in PBST) for 20 minutes per wash. Primary antibodies were diluted 1:100 in blocking buffer and incubated with zebrafish samples overnight at 4 °C in the dark. The following primary antibodies were used: anti-apolipoprotein B (rabbit polyclonal, Abcam, ab20737), anti-PCNA (mouse monoclonal, Cell Signaling Technology, #2586S; clone PC10), anti-acetylated tubulin (mouse monoclonal, Sigma, #T7451; clone 6-11B-1), and anti-Hsp70l (rabbit polyclonal, Creative Diagnostics, #CABT-B661). Following primary antibody incubation, samples were washed three times in blocking buffer for 20 min each. Secondary antibodies were diluted 1:200 in blocking buffer and incubated for 1.5 h at room temperature in the dark. The following secondary antibodies were used: goat anti-mouse IgG (highly cross-adsorbed, Alexa Fluor 546, ThermoFisher, #A-11030), goat anti-rabbit IgG (highly cross-adsorbed, Alexa Fluor 568, ThermoFisher, #A-11011), and goat anti-rabbit IgG (highly cross-adsorbed, Alexa Fluor Plus 647, ThermoFisher, #A-32733). Following secondary antibody incubation, zebrafish were washed three times in blocking buffer for 20 min each and then transferred to PBST. Samples were stored in the dark at 4 °C prior to confocal microscopy.

### Whole-mount zebrafish TUNEL staining

Zebrafish embryos and larvae were fixed overnight at 4 °C in the dark in 4% PFA-PBS and subsequently rinsed three times with PBST. Samples were gradually dehydrated through a series of methanol solutions (25%, 50% and 75% in PBST) for 5 minutes each and stored in 100% methanol at − 20 °C. On the day of staining, zebrafish were rehydrated through a reverse methanol gradient (75%, 50% and 25% methanol in PBST) for 5 min each, followed by four rinses with PBST. For embryos older than 1 dpf, Proteinase K solution (30 μg/ml) in PBST was used for tissue digestion, with incubation times increasing from 5 min at 2 dpf to 15 min at 5 dpf, depending on developmental stage. After digestion, zebrafish were rinsed twice with PBST, fixed in 4% PFA-PBS for 20 min at room temperature, and washed briefly with PBST four times. TUNEL staining was performed using the ApopTag Red In Situ Apoptosis Detection Kit (Sigma). Briefly, zebrafish were incubated with equilibration buffer for 45 min at room temperature, followed by reaction buffer containing TdT enzyme for 1 h at 37 °C. Subsequently, samples were incubated with Stopwash buffer for 1 h at room temperature, rinsed three times with PBST and incubated overnight at 4 °C in the dark with Anti-Dig Rhodamine and blocking solution. Finally, zebrafish were washed three times with PBST before proceeding with confocal microscopy.

### LDL-cholesterol assay

To measure peripheral LDL-cholesterol levels, whole zebrafish at 2 dpf (*n* = 60–90) and 5 dpf (*n* = 20–50) were pooled per experimental group. At 2 dpf embryos were dechorionated and deyolked using calcium-free Ringer’s solution and trituration. Zebrafish were homogenized in PBS and centrifuged to remove remaining yolk remnants. The LDL/VLDL-cholesterol assay was performed using zebrafish homogenates, according to the manufacturer’s instructions (ab65390). LDL/VLDL-cholesterol levels were normalized to protein concentrations using a standardized BCA assay.

### Oil-Red-O staining

Zebrafish were fixed overnight at 4 °C in 4% PFA-PBS and subsequently rinsed four times with PBST. Fixed larvae were pre-bathed in 60% isopropanol (prepared in distilled water) for 1 h at room temperature. 0.5% ORO stock solution was prepared in 100% isopropanol, and a working solution of 0.25% ORO in 60% isopropanol was used for staining. Larvae were incubated in the working solution for 2 h at room temperature. After staining, 2 dpf and 5 dpf zebrafish were rinsed three times in 60% isopropanol, followed by four washes with PBST prior to imaging.

### DASPEI staining

Embryonic zebrafish were intravitally stained with 2-[4-(Dimethylamino)styryl]-1-ethylpyridinium iodide (DASPEI, Sigma) to label neuromast structures. Dechorionated embryos were incubated in 0.005% DASPEI in E3 medium for 20 min at 28 °C in the dark. After staining, embryos were rinsed three times with fresh E3 medium before imaging.

### BODIPY FL C5-ceramide staining

BODIPY FL C5-ceramide (ThermoFisher) was prepared as a stock solution in DMSO and diluted to a working concentration of 10 μM in E3 medium. Live embryos were incubated in the staining solution for 1 h at 28 °C in the dark. After staining, zebrafish were rinsed three times with E3 medium prior to imaging.

### Whole-mount zebrafish fluorescence RNA in situ hybridization (FISH)

Zebrafish embryos and larvae were fixed overnight at 4 °C in the dark with 4% PFA-PBS and subsequently rinsed three times with PBST. Samples were gradually dehydrated through a series of methanol solutions (25, 50 and 75% in PBST) for 5 minutes each and stored in 100% methanol at − 20 °C. On the day of staining, zebrafish were rehydrated through a reverse methanol gradient (75%, 50% and 25% methanol in PBST) for 5 min each, followed by four rinses with PBST. For embryos older than 1 dpf, Proteinase K solution (30 μg/ml) in PBST was used for tissue digestion, with incubation times increasing from 5 minutes at 2 dpf to 15 min at 5 dpf, depending on developmental stage. After digestion, zebrafish were rinsed twice with PBST, fixed in 4% PFA-PBS for 20 min at room temperature, and washed briefly with PBST four times. To detect *hsp70* expression, the HCR RNA-FISH protocol and reagents were used, as per the manufacturer’s instructions (Molecular Instruments). Briefly, zebrafish were pre-hybridized in probe hybridization buffer for 30 min at 37 °C, then incubated overnight at 37 °C with a custom-made *hsp70l* mRNA probe, which also binds *hsp70.2*, *hsp70.1* and *hsp70.3* with > 80% coverage. Following hybridization, samples were washed four times for 15 min each with probe wash buffer at 37 °C, followed by two brief rinses with 5x sodium chloride sodium citrate (SSC) containing 0.1% Tween20 (SSCT). Zebrafish were pre-amplified with amplification buffer for 30 min at room temperature and incubated overnight at room temperature in the dark with fluorescently conjugated hairpin solutions to amplify *hsp70l* signal. Excess hairpins were removed by washing four times with SSCT, followed by three additional washes with PBST prior to confocal microscopy.

### Reverse transcription quantitative polymerase chain reaction (RT-qPCR)

Total RNA was extracted from pooled samples of *n* = 14–20 zebrafish collected at 1 dpf, 2 dpf or 3 dpf using Trizol (ThermoFisher). Complementary DNA was synthesized from total RNA using iScript SuperMix (Bio-Rad). Real-time quantitative PCR (RT-qPCR) reactions were performed in triplicate in 96-well plates using iTaq Universal SYBR Green Supermix (Bio-Rad) and a CFX96 Touch Real-Time PCR Detection System (Bio-Rad). The thermal cycling protocol included an initial denaturation step followed by 39 cycles of denaturation at 95 °C for 10 s, annealing/extension at 60 °C for 1 min, followed by melting curve temperature ramping. Relative gene expression levels were calculated using the delta CT (cycle number) method after normalizing each sample to *gapdh*. Primer sequences used for RT-qPCR are listed:

*ddit3* Fw: ACACCATGGTAAACGGAGGC

*ddit3* Rv: GCAGGATGATGTGTCGGGAA

*hspa5* Fw: CTCTGACCCGAAGAAGCCAG

*hspa5* Rv: GAACTCCTTCACCAGCTGCT

*atf4a* Fw: CCGTCTCCTCCTGAAGAAGC

*atf4a* Rv: ATGTGAGACGGAGAGAGGCT

*dnajb1b* Fw: GAAGGACTGAAGGGAGGTGC

*dnajb1b* Rv: GCCCTCCGAAGAACTCTGAG

*gapdh* Fw: GATACACGGAGCACCAGGTT

*gapdh* Rv: CGTTGAGAGCAATACCAGCA

### Morpholino injection

To inhibit cardiac contractility and blood flow in zebrafish embryos, a *tnnt2a* antisense morpholino oligonucleotide (GeneTools LLC) was utilized. The morpholino was resuspended in RNAse-free water to a stock concentration of 1 mM and diluted to working concentrations prior to injections. For complete arrest of heart contraction and blood flow, 2 ng of *tnnt2a* morpholino was injected into one-cell stage embryos. To achieve reduced heart contraction and blood flow, a 0.4 ng dose (1:5 stock dilution) of *tnnt2a* morpholino was injected into one-cell stage embryos. Standard control morpholino was injected as a negative control, resulting in embryos with normal heart contractility and blood flow.

tnnt2a ATG (translation blocking) morpholino sequence:

5’-CATGTTTGCTCTGATCTGACACGCA-3’^70^

Standard control morpholino sequence:

5’-CCTCTTACCTCAGTTACAATTTATA-3’

### Zebrafish embryo dissociation and endothelial cell isolation

Two-day post fertilization Tg(*flk:GFP*) embryos of *ldlr* + /+ and *ldlr*-/- genotypes, representing physiological, reduced or static blood flow groups, were anesthetized using 40 μg/mL tricaine in E3 medium until immobile. Approximately 200 embryos per group were pooled and enzymatically dissociated using preheated 0.25% Trypsin-EDTA (Gibco) at 28 °C for 20 min, with mechanical homogenization performed every 5 min to ensure complete dissociation. Following dissociation, embryos were washed with PBS supplemented with 1% Fetal Bovine Serum (FBS). Cell pellets were collected by centrifugation and resuspended in PBS containing 1% FBS in preparation for fluorescence-activated cell sorting (FACS). A forward and side scatter (FSC/SSC) size selection gate was constructed and validated through back-gating of cells with high GFP expression. Vascular endothelial (GFP + ) and non-endothelial (GFP-) single-cell fractions were equally sorted according to the presence of GFP signal using a BD FACSAria Fusion instrument and BD FACSDiva (version 9.0.1) software. Sorted cells were subsequently prepared for single-cell RNA sequencing.

### Human aortic endothelial cell (HAEC) culture and flow exposure

Primary HAECs were harvested from the ascending and descending aorta of a single donor in a single batch (Promocell, #C-12271) and cultured in endothelial cell growth medium (Promocell, #C-22210) supplemented with 7% FBS, 100 U/mL penicillin, and 100 µg/mL streptomycin. Cells were maintained until passages P3-P5 before exposure to different flow conditions.

HAECs were subjected to physiologic or high shear stress (hss, 30 dynes/cm^2^), reduced or low shear stress (lss, 2.5 dynes/cm^2^) or static flow (s, 0 dynes/cm^2^) for 48 hours in a flow-controlled bioreactor. HAECs were seeded at a density of 80000 cells per cm^2^ in IbidiTreat µ-Slide I Luer^0.8^ flow chambers for 24 hours under static conditions to allow cell attachment^71^. Cell attachment was verified microscopically prior to initiating flow. The flow loops included a 10 mL glass reservoir, ISMATEC PharMed BPT pump tubing with an inner diameter of 2.79 mm (Cole Parmer, #95809-48), Silastic laboratory tubing, and the Ibidi flow chamber. All components, except the flow chamber, were autoclaved before assembly. Each flow loop utilized 15 mL of media, and laminar flow was delivered using an 8-roller, 12-channel peristaltic pump (ISMATEC MCP, #78002-00) programmed to apply a specified level of shear stress on HAECs primarily adhered to the center of IbidiTreat µ-Slide I Luer^0.8^, as per manufacturer’s instructions.

After 48 h of flow stimulation, HAECs were washed with PBS, detached using 0.05% Trypsin-EDTA for 5 min, and collected in a microcentrifuge tube with complete medium. Cells were centrifuged, resuspended in PBS and prepared as a single-cell suspension ( ~ 100,000 cells per flow chamber) for single-cell RNA sequencing.

### Single-cell RNA sequencing (scRNA-seq) of embryonic zebrafish cells and HAECs

ScRNA-seq was performed on embryonic zebrafish cells and HAECs using the Chromium single cell 3’ v3 protocol (10x Genomics) according to the manufacturer’s instructions. cDNA libraries were prepared for sequencing to capture transcriptomic profiles at single-cell resolution. Processed data files, including the mapped barcode, feature and matrix files, were generated using CellRanger software (version 6.0.1, 10x Genomics). These output files served as the basis for downstream analysis.

### Acute CRISPR/Cas9 genome editing in zebrafish

The CRISPR guide RNA (gRNA) sequence GAGATCATCGCCAACGACCAGGG (Integrated DNA Technology) was designed to target exon 1 of *hsp70l*, *hsp70.1* and *hsp70.3*. The *hsp70* gRNA was annealed with *trans*-activating crRNA (tracrRNA) and incubated with Alt-R S.p. HiFi Cas9 Nuclease V3 (Integrated DNA Technology) to form ribonucleoprotein complexes, following the manufacturer’s instructions. A 1-nL volume of the combined ribonucleoprotein complex was microinjected into one-cell stage zebrafish embryos. Phenotypic assessments were performed at 1 dpf, 2 dpf and 3 dpf. Control embryos were microinjected with tracrRNA alone. To confirm targeted editing of exon 1, genomic DNA was extracted, and Sanger sequencing was performed using the following primers:

*hsp70* primer Fw: CGCTATTGGCATTGACCTGG

*hsp70* primer Rv: CGATCAGCCTCTTGGCATCA

### Pharmacological zebrafish treatments

Embryonic zebrafish were manually dechorionated at 1 dpf using Dumont Tweezers and individually placed in 24-well plates. Drug treatments were conducted at 28 °C. 2,3-Butanedione monoxime (BDM, Sigma) was dissolved in zebrafish E3 medium to a working concentration of 10 mM. All other pharmacological compounds were stored as 10 mM stock solutions in DMSO and diluted in E3 medium to the following working concentrations: 50 μM VER-155008 (Tocris), 50 μM 17-DMAG (Tocris), and 100 μM SB-203580 (MedChemExpress). Control embryos were incubated with 0.5% or 1% DMSO in E3 medium. Following 24 h of drug exposure, embryos were washed thoroughly in E3 medium and prepared for downstream staining/imaging.

### Sudan Black B staining

Zebrafish were fixed overnight in 4% PFA-PBS at 4 °C and subsequently rinsed four times with PBST. Fixed zebrafish were stained with 0.045% Sudan Black B (Sigma) in 70% ethanol for 20 min at room temperature. Following staining, zebrafish were briefly washed three times with 70% ethanol and rehydrated with three rinses of PBST prior to imaging.

### Neutral Red staining

Live zebrafish were incubated in 5 μg/ml Neutral Red (ThermoFisher) dissolved in E3 medium. Staining duration varied based on developmental stage: 2 h for 2 dpf and overnight for 3 dpf zebrafish. Staining was conducted in the dark at 28 °C. Zebrafish were washed three times in E3 medium prior to imaging.

### CellRox staining

CellRox Orange (ThermoFisher) was dissolved in DMSO and used at a working concentration of 5 μM in E3 medium. Live zebrafish embryos were incubated with the dye for 1 h in the dark at 28 °C. Post-staining, zebrafish were rinsed three times in E3 medium before imaging.

### Tail fin vessel amputation and epithelial resection in zebrafish

A developmental model of tail vessel amputation was established in embryonic and larval zebrafish. Zebrafish embryos at 2 dpf were anesthetized in 40 μg/mL tricaine (E3 medium), and the tail vasculature was visualized using stereomicroscopy. A sterile scalpel (Sklar) was used to transect the two most posterior intersegmental vessels, including the caudal end of the dorsal longitudinal anastomotic vessel, dorsal aorta and caudal vein. Amputated embryos were transferred individually to 24-well plates and imaged every 24 h up to 4 days post-amputation to monitor regenerative angiogenesis.

Standard tail fin epithelial resection was performed using a sterile scalpel. The transected tissue area was restricted to the distal epithelial tissue located caudal to the vascular loop and notochord, taking care to avoid both structures, as performed previously^60^.

### Thrombosis assay in zebrafish

FeCl_3_ solution (2.5%) prepared in E3 medium was used to induce thrombosis in 2 dpf and 3 dpf zebrafish. Individual zebrafish were transferred to 96-well plates, and FeCl_3_ was added to each well. Clotting time was determined by observing blood flow occlusion in the dorsal aorta and caudal vein using brightfield stereomicroscopy.

### Image analysis

All images were processed and analyzed using Fiji (ImageJ, version 2.16) software (National Institutes of Health). Confocal microscopy images of Tg(*flk:GFP*) zebrafish were captured as 3D z-stacks and processed into maximum intensity projections (MIPs) to quantify vascular phenotypes and/or fluorescence staining.

#### Vascular Oil Red O (ORO) opacity

Brightfield images were first converted to 32 bit. A line of 600 μm length (running posteriorly from the end of the cloaca) was positioned through the midline of the dorsal aorta or caudal vein. The plot profile function in Fiji was used to quantify gray values, and the mean gray value in the dorsal aorta and caudal vein was normalized to adjacent avascular notochord tissue measured in the same zebrafish. The calculation: 1-(mean ORO gray value in vessel/mean avascular background), was used to estimate mean vascular ORO opacity, where the value between 0-1 corresponds to signal intensity.

#### Vascular morphology quantification

The following morphological vascular parameters were quantified: CVP loop number (dark hollow structures, area ≥ 6 μm^2^ and intraluminal diameter ≥ 4 μm), CVP diameter (length from dorsal to ventral extremities of the CVP) and largest loop area (intraluminal perimeter of the largest loop). The CVP region was defined per image as the first to eleventh intersegmental vessel running posteriorly from the end of the cloaca. The ventral CVP (vCVP) sub-region was defined by the most ventral patent vessel that exhibits a luminal separation from the rest of the CVP. The dorsal CVP (dCVP) sub-region formed the connecting vessels situated dorsally to the vCVP, ending adjacent to the ventral side of the DA^20^.

#### Whole-mount staining quantification

For immunofluorescence (Apolipoprotein B, PCNA, Acetylated tubulin and Hsp70l) and TUNEL staining, confocal images were cropped to the vessel of interest (in *x*, *y* and *z* dimensions), background fluorescence was subtracted in the stained channel, and signal thresholding was applied. Thresholding was used to quantify the presence of individual cells (diameter > 4 μm, PCNA or Hsp70l), thresholded area (ApoB), cilia number and average cilia length (Acetylated tubulin). For apoptotic staining, TUNEL+ and *flk:GFP *+ cells (diameter > 4 μm) were quantified within each indicated trunk vessel or total cerebral vasculature. For FISH staining, *hsp70l+* and *flk:GFP *+ cells (diameter > 6 μm) were quantified within each indicated trunk vessel or total cerebral vasculature.

#### Blood flow visualization and quantification in zebrafish

Time-lapse video recordings of red blood cell movement in Tg(*gata1:dsRed*) zebrafish were recorded using an EVOS Auto2 microscope for 10–15 s at a capture rate of 25 frames per second. Temporal overlay (“hyperstacking”) of individual frames over 8 seconds was performed using Fiji’s “Temporal-Color Code” tool. Each overlaid time point corresponds to a different hue, resulting in a single image that summarizes red blood cell movement routes through the trunk vasculature.

To track RBC movement and quantify RBC behaviors in flow, the Fiji plugin Trackmate was used^72^. First, RBC flow was refined to a vascular compartment (DA, CVP, dCVP or vCVP) by cropping to the region of interest across the defined area (first to eleventh ISV posterior from end of cloaca). The following Trackmate parameters were applied to blood flow recordings (across 120 frames): spot diameter of 6 μm (2 dpf) or 5 μm (5 dpf), QC value of 1 and Laplacian of Gaussian spot detection. LAP tracker and remaining default parameters were applied to perform single RBC tracking, with mean track velocity values and color-coded track speed images exported. RBCs with movement distances of less than 7 μm were excluded (to remove non-motile or artefactual detections), and RBC velocity values were averaged per zebrafish vessel recording.

#### Vascular regeneration and immune cell wound recruitment

To quantify tail vessel regenerative angiogenesis per time point, the neoangiogenesis region was defined by the Tg(*flk:GFP*) signal spanning from the most posterior intersegmental vessel to the most distal vessel sprout. The area of interest was cropped, and thresholding was applied to quantify angiogenic area.

To quantify neutrophil (*mpx:GFP*) and macrophage (*mfap4:tdTomato*) wound recruitment per time point, simultaneously acquired brightfield and epifluorescence images were used. The wound region was defined by a rectangular area from the blastema (posterior wound edge) anteriorly to where the ventral tail fin fold runs in parallel to the most posterior point of the caudal vein. This anatomical landmark was chosen as it was reproducibly identified per fish using brightfield microscopy. The rectangular region of interest was applied to *mpx:GFP* and *mfap4:tdTomato* tail fin images, cropped and thresholded. Individual neutrophils were quantified within the thresholded region of interest. Wound-recruited macrophages displayed complex dendritic morphology, therefore, the thresholded signal was quantified within the region of interest.

#### Immune cell or HSC quantity in the CHT

The number of neutrophils (*mpx:GFP*), macrophages (*mfap4:tdTomato*) and HSCs (*cd41:GFP*^*low*^) within the CHT were quantified from 1 to 3 dpf. A rectangular region of interest was positioned over the CHT, starting from the end of cloaca, with a length varying depending on developmental stage (1 dpf = 400 μm, 2 dpf = 650 μm and 3 dpf = 750 μm). This region was cropped, the background signal was subtracted, thresholding and watershed segmentation were applied to quantify individual cells. For HSCs, *cd41:GFP*^*low*^ expressing cells were quantified within the CHT region at 2 dpf, as previously characterized^73^.

#### CellRox quantification

Zebrafish stained with CellRox orange were imaged using EVOS Auto2 microscopy, and epifluorescence images were quantified by measuring total fluorescence intensity. A rectangular region of interest encapsulating the whole body of the zebrafish was applied, images were cropped, and the intensity density measured was normalized to the background signal. For tail wound quantification, a region of interest (120 μm x 280 μm) was positioned perpendicularly over the wound edge, and the CellRox fluorescence intensity density measured was normalized to the background signal.

### ScRNA-seq analysis

#### Pre-processing and dimensionality reduction

Raw feature count tables for each experimental group were analyzed in R (version 1.3.1) using the *Seurat* package (Version 5.0.3)^74,75^. Quality control criteria were applied as follows: Embryonic zebrafish cells were included if at least 500 Unique Molecular Identifiers (UMIs), 200 genes, and a gene-to-UMI ratio greater than 0.8 were detected; thus, cells with more than 20% mitochondrial reads were excluded. HAECs were included if at least 500 UMIs, 250 genes, and a gene-to-UMI ratio greater than 0.8 were detected; thus, cells with more than 20% mitochondrial reads were excluded. Post quality control, 12,765 zebrafish cells and 10,083 HAECs exposed to various flows conditions were retained for further analysis.

The data were normalized by scaling each cell’s expression values to 10,000 total UMIs, followed by log-normalization using the *Seurat* “NormalizedData” function. Highly variable genes were identified using the “FindVariableFeatures” function to enable downstream analyses.

Scaled, normalized count data were subjected to principal component analysis, using the first 40 principal components (PCs) for dimensionality reduction. The uniform manifold approximation and projection (UMAP) algorithm, implemented in *Seurat*, was then used to project the 40 PCs into a two-dimensional space. This visualization revealed cell type stratification and distinct clustering based on genotype and/or flow state.

#### Differential Gene Expression Analysis and Gene Set Enrichment

Differentially expressed genes between flow and genotype conditions in zebrafish and HAEC datasets were identified using the *Seurat* “FindMarkers” function. Analysis criteria included a minimum log-fold change threshold of 0.1. Statistical *p*-values were computed via the Wilcoxon rank-sum test. Subpopulation-specific markers from clustering analysis were determined using the *Seurat* “FindAllMarkers” function with the same minimum log-fold change threshold of 0.1 and Wilcoxon rank-sum statistical test. Zebrafish cell cluster identities were annotated by examining the most significant genes identified using the “FindConservedMarkers” function (log-fold change threshold of 0.25). These data were cross-referenced with publicly available embryonic zebrafish scRNA-seq datasets^76–80^ for specific annotation of neural cell subsets^76,78,80^ and all other cell types^76,77,79^. For vascular endothelial cell subset annotation in zebrafish, we first examined for enrichment of pan endothelial markers (*cdh5*, *kdrl* and *tie1*). Endothelial cells were then segregated by anatomical location based on cranial (*edn1*, *foxc1b* and *adarb1a*) and trunk endothelial (*sele*, *stab1*, and *mrc1a*) cell expression enrichment. The *hsp70* + EC population in *ldlr*-/- embryos was identified as a subset of the larger trunk EC cluster, and sub-segregated based on the top four most significantly enriched genes, including the *hsp70* orthologs (*hsp70l*, *hsp70.1*, *hsp70.2* and *hsp70.3*).

Gene set enrichment analysis was performed on differentially expressed genes using the R library *fgsea* (version 1.24.0), applying an adjusted *p*-value threshold of 0.05. Gene sets for *Homo sapiens* and *Danio rerio* were annotated using the C5 ontology gene sets from the Molecular Signatures Database^81^. This analysis facilitated the identification of biological processes and pathways significantly associated with flow and genotype conditions in both zebrafish and HAEC datasets.

#### Pseudotime trajectory analysis

Pseudotime trajectory analysis was conducted on zebrafish scRNA-seq data using the *Monocle3* R package^82^. The analysis focused on all trunk endothelial cell populations across experimental groups (*ldlr* + / + and *ldlr*-/- under physiological flow, reduced flow or static flow conditions). Data were preprocessed using a standard workflow including normalization, selection of highly variable features, data scaling, and linear dimensionality reduction. The *Monocle3* preprocessing pipeline was applied at various resolutions, with UMAP utilized for non-linear dimensionality reduction. Trajectory paths were inferred using the “learn_graph” function, with the lineage origin defined as endothelial cells that most transcriptionally resemble undifferentiated cells. This pseudotime origin point was consistently applied across all experimental groups to ensure comparability. Pseudotime computation and cell ordering were subsequently performed using the “plot_cells” and “order_cells” functions, respectively. This analysis enabled the identification of developmental trajectories and transcriptional changes in endothelial cells under varying flow and genotype conditions.

#### scRNA-seq Integration

To integrate single-cell sequencing datasets of HAECs exposed to similar flow and shear stress conditions but sequenced using different platforms (10X Genomics and SeqWell S3), an anchor-based integration workflow implemented in *Seurat* was utilized^74^. This approach harmonized shared cell types and states across platforms, enabling precise comparative analysis while enhancing statistical power. Initially, raw data from both sequencing platforms were normalized, and highly variable features were identified using the *Seurat* “FindVariableFeatures” function. Data from the 10X Genomics and SeqWell S3 platforms were then separated into distinct layers. Initial analysis without integration revealed clustering patterns influenced by both cell type and sequencing platform. Integration was performed using *Seurat’s* “IntegrateLayers” function, which employs canonical correlation analysis to align cells from equivalent subpopulations. This step ensured the removal of technical platform biases while preserving biological variation. The integrated dataset underwent unsupervised clustering and dimensionality reduction using the UMAP algorithm, resulting in a unified representation of HAECs across platforms.

### Statistical analysis

All data are presented as mean ± standard deviation (s.d.) or standard error of the mean (s.e.m.), as specified. Data were analyzed in GraphPad Prism 10 software. Normal distribution of data was confirmed using the Shapiro-Wilk test. Comparisons between two groups were performed using unpaired or paired two-tailed Student’s *t* tests. For comparisons involving more than two groups, an ordinary one-way or two-way analysis of variance (ANOVA) followed by Holm-Sidak’s post-hoc multiple comparison adjustment was performed. Fisher’s exact test was employed for comparing categorical events between two groups. Independent experiments were performed on at least three different clutches of zebrafish on different days to ensure biological reproducibility. Statistical significance was defined as a *P-*value or adjusted *P-*value  < 0.05.

### Reporting summary

Further information on research design is available in the Nature Portfolio Reporting Summary linked to this article.

## Supplementary information

Supplementary Information

Peer Review file

Description of Additional Supplementary Files

Supplementary Data 1

Supplementary Movie 1-10

Reporting Summary

## Source data

Source Data

## Data Availability

ScRNA-seq data from zebrafish and HAEC models have been submitted to the Gene Expression Omnibus repository and made publicly available under the accession number GSE275308. In addition, the HAEC data used for integration that were sequenced with SeqWell S3 are publicly available at GSE212388^71^. All computational analyses were performed using open-source software. Source data are provided in this paper.

## Code availability

The open-source software packages for performing the scRNA-seq analyses have been outlined at: https://github.com/aryan-kaveh/scRNA-seq-flow-mediated-EC-remodeling-ldlr.
