# Supplementary material for: Flow-mediated endothelial remodeling and inflammation drive developmental vascular susceptibility in ldlr loss of function
Source: Nat Commun. 2026 May 21;17:6926. doi: 10.1038/s41467-026-72756-3 (PMC13388945; doi:10.1038/s41467-026-72756-3)
Supplement: Supplementary file 1 — Supplementary Information [file 41467_2026_72756_MOESM1_ESM.pdf]

Flow-mediated endothelial remodeling and inflammation drives developmental vascular susceptibility in *ldlr* loss of function

Kaveh et al., 2026: Supplementary Information

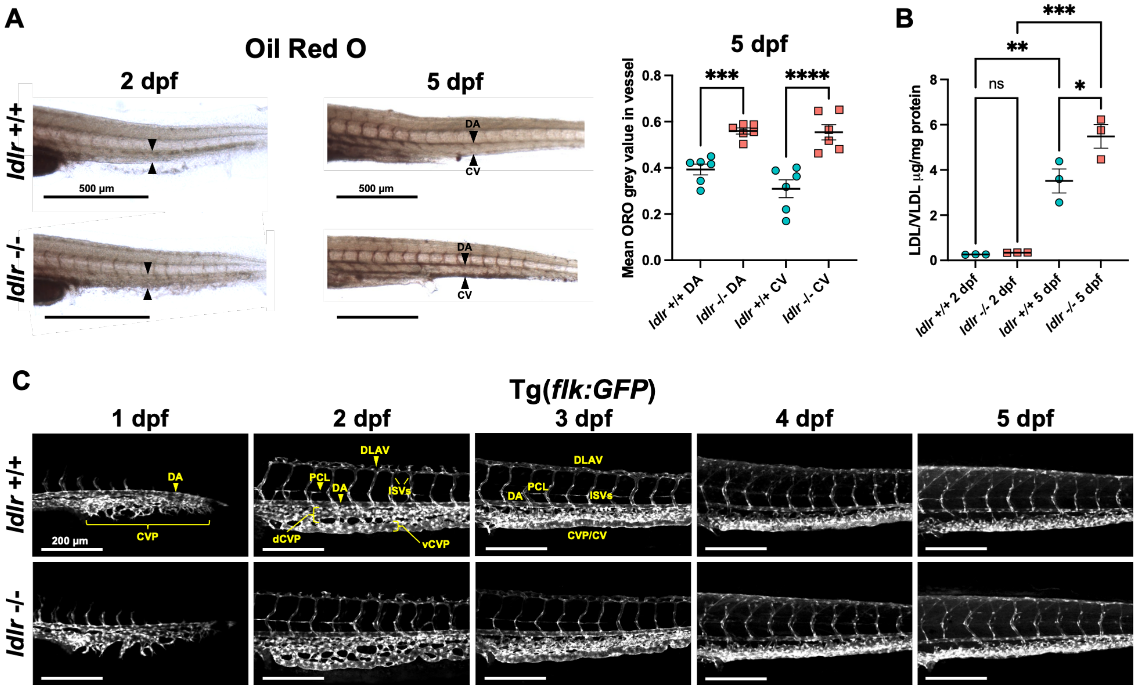

Figure S1. Validation and vascular characterization of *ldlr-/-* zebrafish larvae and embryos. (A) Representative brightfield microscopy of Oil Red O (lipid) stained *ldlr*+/+ (wild-type) and *ldlr*-/- zebrafish trunk region at 2 days post fertilization (dpf) and 5 dpf. Downward arrowheads indicate the dorsal aorta (DA) and upward arrowheads indicate the caudal vein (CV). At 5 dpf, lipids are concentrated in the trunk vasculature (left). Mean Oil Red O (ORO) grey value in the DA and CV of *ldlr*+/+ (n = 6) and *ldlr*-/- (n = 6) zebrafish at 5 dpf. Mean grey value through the vessel was normalised to adjacent avascular tissue per stained zebrafish. Data are mean ± s.e.m. One-way ANOVA and Holm-Sidak's multiple comparison used; 2 independent experiments (right). **(B)** LDL/VLDL measurements (µg/mg protein) from *ldlr*+/+ and *ldlr*-/- homogenates at 2 dpf (n = 60-90 pooled zebrafish) and 5 dpf (n = 20-50 pooled zebrafish). Data are mean ± s.e.m. Two-way ANOVA and Holm-Sidak's multiple comparison used; 3 independent experiments. **(C)** Confocal microscopy of Tg(*flk:GFP*) trunk vasculature in *ldlr*+/+ and *ldlr*-/- zebrafish between 1-5 dpf. Trunk vessels from dorsal to ventral locations indicated: DLAV (dorsal longitudinal anastomotic vessel), ISVs (intersegmental vessels), PCL (parachordal lymphatic), DA, dCVP (dorsal caudal venous plexus), vCVP (ventral caudal venous plexus) and CV.
**** $P < 0.0001$, *** $P < 0.001$, ** $P < 0.01$, * $P < 0.05$, ns, not significant. Source data are provided as a Source Data file.

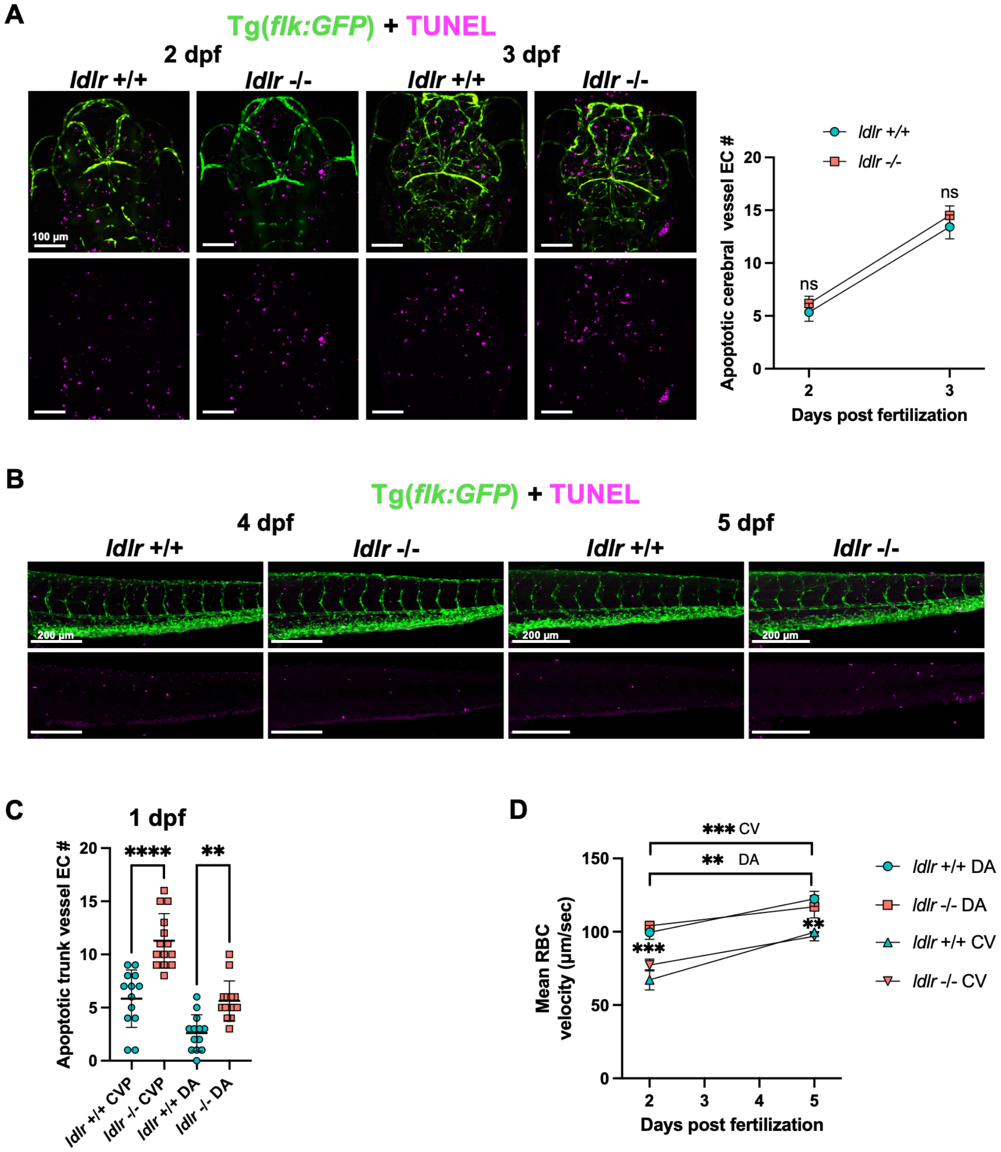

Figure S2. Phenotypic vascular characterization in *ldlr-/-* zebrafish embryos and larvae. (A) Confocal microscopy of TUNEL stained Tg(*flk:GFP*) *ldlr*+/+ and *ldlr*-/- zebrafish cerebral vasculature at 2 dpf and 3 dpf (left). Total number of apoptotic endothelial cells in the cerebral vasculature between *ldlr*+/+ (n = 12) and *ldlr*-/- (n = 12) zebrafish at 2 dpf and 3 dpf. Data are mean ± s.e.m. Two-way ANOVA and Holm-Sidak's multiple comparison used; 2 independent experiments (right). **(B)** Confocal microscopy of TUNEL stained Tg(*flk:GFP*) *ldlr*+/+ and *ldlr*-/- zebrafish trunk vasculature at 4 and 5 dpf. **(C)** Number of apoptotic endothelial cells in the dorsal aorta (DA) and caudal venous plexus (CVP) between *ldlr*+/+ (n = 13) and *ldlr*-/- (n = 14) zebrafish at 1 dpf. Data are mean ± s.d. One-way ANOVA and Holm-Sidak's multiple comparison used; 3 independent experiments. **(D)** Mean red blood cell velocity (µm/sec) measured in the DA and CV of *ldlr*+/+ (n = 6) and *ldlr*-/- zebrafish (n = 6) at 2 dpf and 5 dpf. Data are mean ± s.e.m. Two-way ANOVA and Holm-Sidak's multiple used for *ldlr*+/+ comparisons between DA and CV at 2 dpf and 5 dpf (different vessels, same timepoint) and for comparisons between DA or CV at 2 dpf and 5 dpf (same vessels, different timepoints); 2 independent experiments. **** $P < 0.0001$, *** $P < 0.001$, ** $P < 0.01$, * $P < 0.05$, ns, not significant. Source data are provided as a Source Data file.

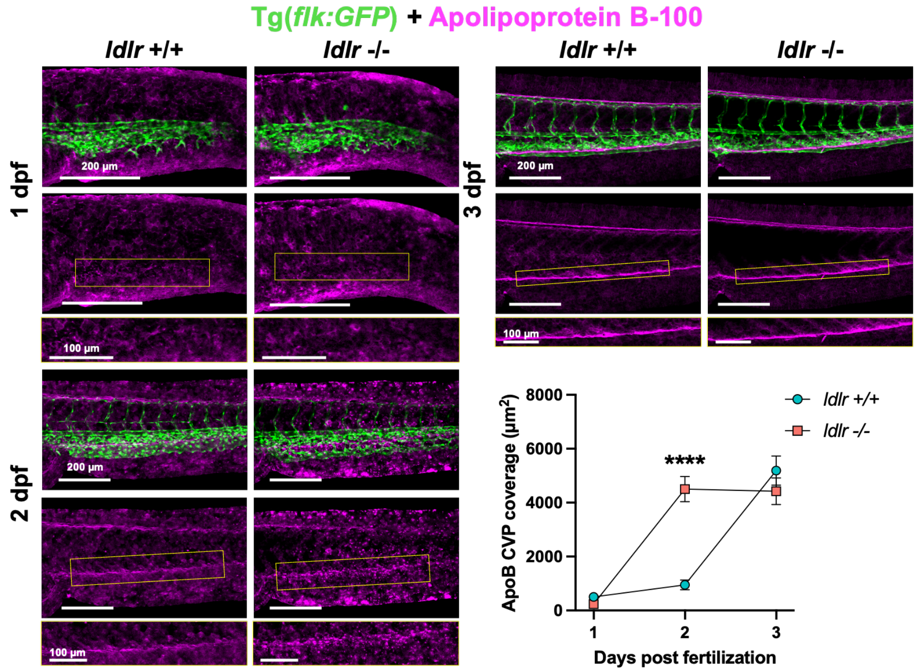

Figure S3. Trunk vessel Apolipoprotein B immunofluorescence staining in *ldlr-/-* zebrafish embryos and larvae. Confocal microscopy of Apolipoprotein B stained Tg(*flk:GFP*) *ldlr*+/+ and *ldlr*-/- zebrafish trunk vasculature at 1 dpf, 2 dpf and 3 dpf. Boxed region indicates caudal venous plexus (CVP) area used for measurements. Inset of CVP ApoB coverage indicated at 1-3 dpf. Area of ApoB coverage in the CVP between *ldlr*+/+ (*n* = 13) and *ldlr*-/- (*n* = 11-13) zebrafish at 1 dpf, 2 dpf and 3 dpf. Data are mean ± s.e.m. Two-way ANOVA and Holm-Sidak's multiple comparison used; 3 independent experiments. **** $P < 0.0001$ (bottom, right). Source data are provided as a Source Data file.

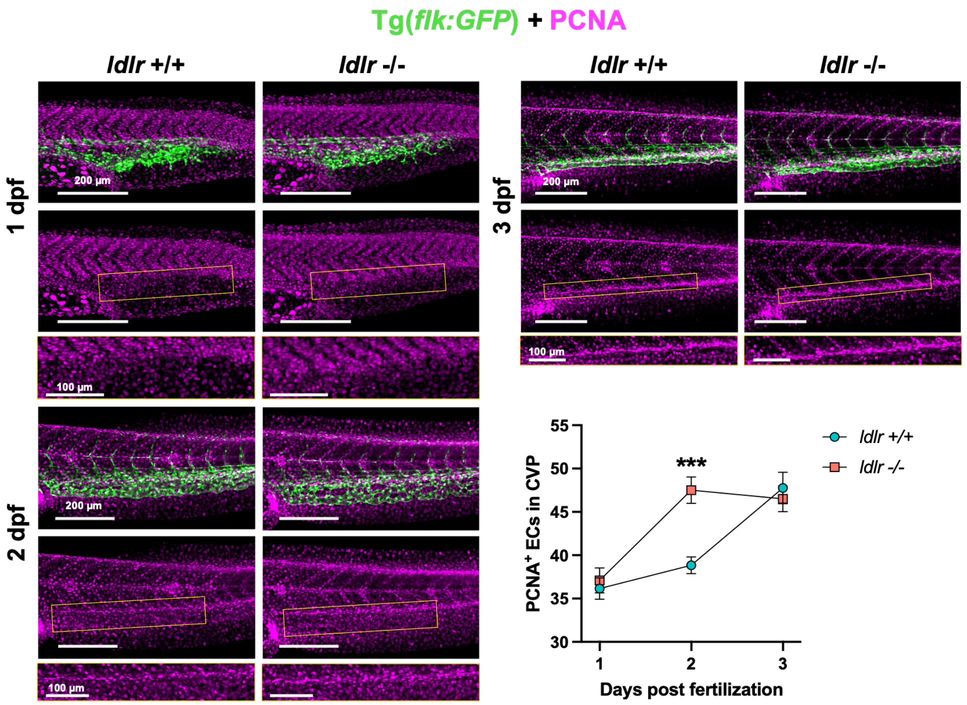

Figure S4. Trunk vessel PCNA immunofluorescence staining in *ldlr*-/- zebrafish embryos and larvae. Confocal microscopy of PCNA stained Tg(*flk:GFP*) *ldlr*+/+ and *ldlr*-/- zebrafish trunk vasculature at 1-3 dpf. Boxed region indicates caudal venous plexus (CVP) area used for measurements. Inset of CVP PCNA+ cells indicated at 1 dpf, 2 dpf and 3 dpf. Number of $PCNA^+$ endothelial cells in the CVP between *ldlr*+/+ (n = 12) and *ldlr*-/- (n = 12) zebrafish at 1 dpf, 2 dpf and 3 dpf. Data are mean ± s.e.m. Two-way ANOVA and Holm-Sidak's multiple comparison used; 3 independent experiments. *** $P < 0.001$ (bottom, right). Source data are provided as a Source Data file.

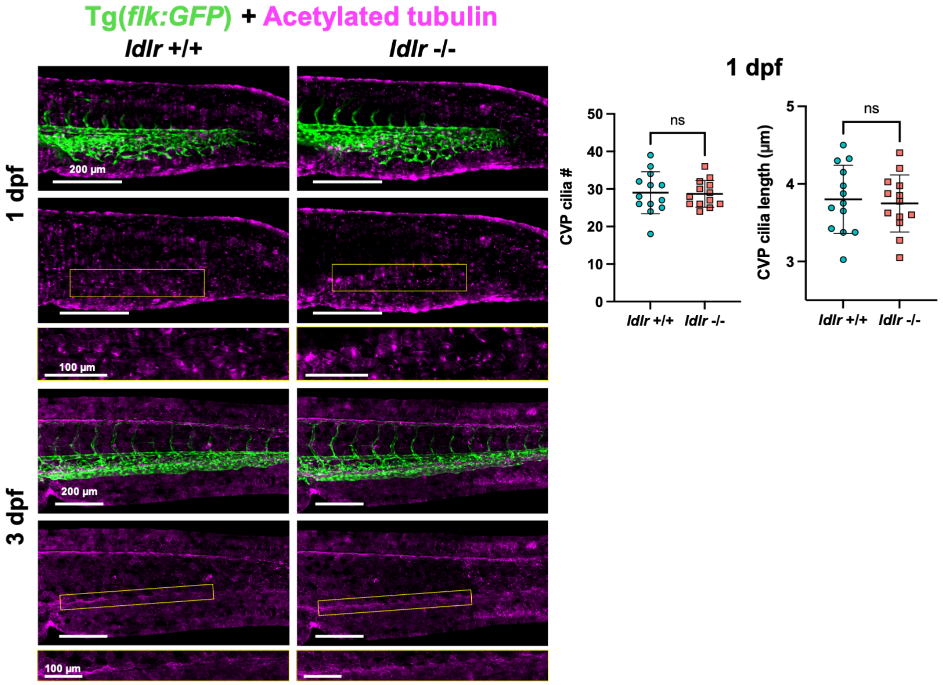

Figure S5. Trunk vessel Acetylated tubulin immunofluorescence staining in *ldlr-/-* zebrafish embryos and larvae. Confocal microscopy of Acetylated tubulin stained Tg(*flk:GFP*) *ldlr*+/+ and *ldlr*-/- zebrafish trunk vasculature at 1 dpf and 3 dpf. Boxed region indicates caudal venous plexus (CVP) area used for measurements. Inset of CVP cilia indicated at 1 dpf and 3 dpf (left). CVP cilia number and average cilia length (µm) in *ldlr*+/+ (*n* = 13) and *ldlr*-/- (*n* = 13) zebrafish at 1 dpf. Data are mean ± s.d. Unpaired two-tailed t-test used; 3 independent experiments. ns, not significant (right). Source data are provided as a Source Data file.

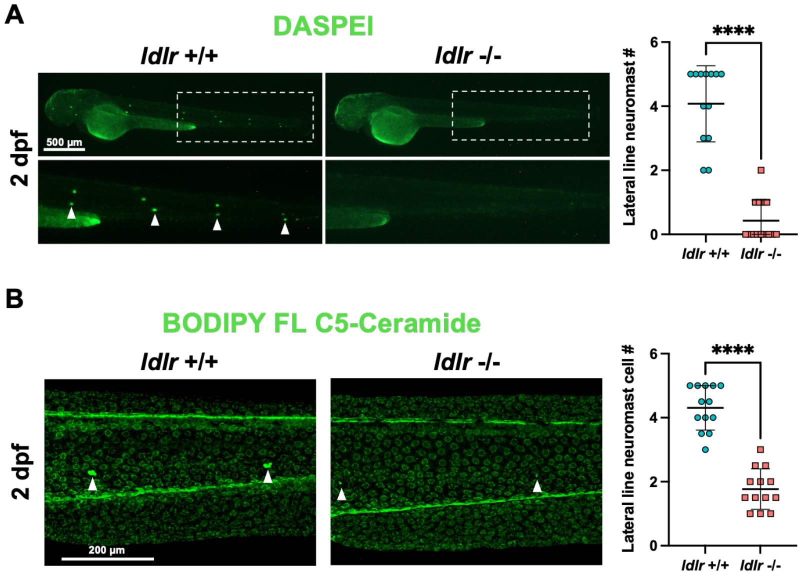

Figure S6. Assessment of mechanosensory neuromasts in *ldlr*-/- zebrafish. **(A)** Epifluorescence microscopy of DASPEI-stained posterior (trunk) lateral line neuromasts in *ldlr*+/+ and *ldlr*-/- zebrafish at 2 dpf. Boxed region indicates neuromast inset and arrowheads indicate functionally active neuromasts (left). Neuromast quantification in *ldlr*+/+ (*n* = 13) and *ldlr*-/- (*n* = 14) zebrafish. Data are mean ± s.d. Unpaired two-tailed t-test used; 3 independent experiments (right). **(B)** Confocal microscopy of BODIPY FL C5-Ceramide-stained lateral line neuromasts in *ldlr*+/+ and *ldlr*-/- zebrafish at 2 dpf. Arrowheads indicate neuromast cells (left). Average number of BODIPY FL C5-Ceramide-stained lateral line neuromast cells per neuromast in *ldlr*+/+ (*n* = 13) and *ldlr*-/- (*n* = 13) zebrafish at 2 dpf. Data are mean ± s.d. Unpaired two-tailed t-test used; 3 independent experiments (right). **** *P* < 0.0001. Source data are provided as a Source Data file.

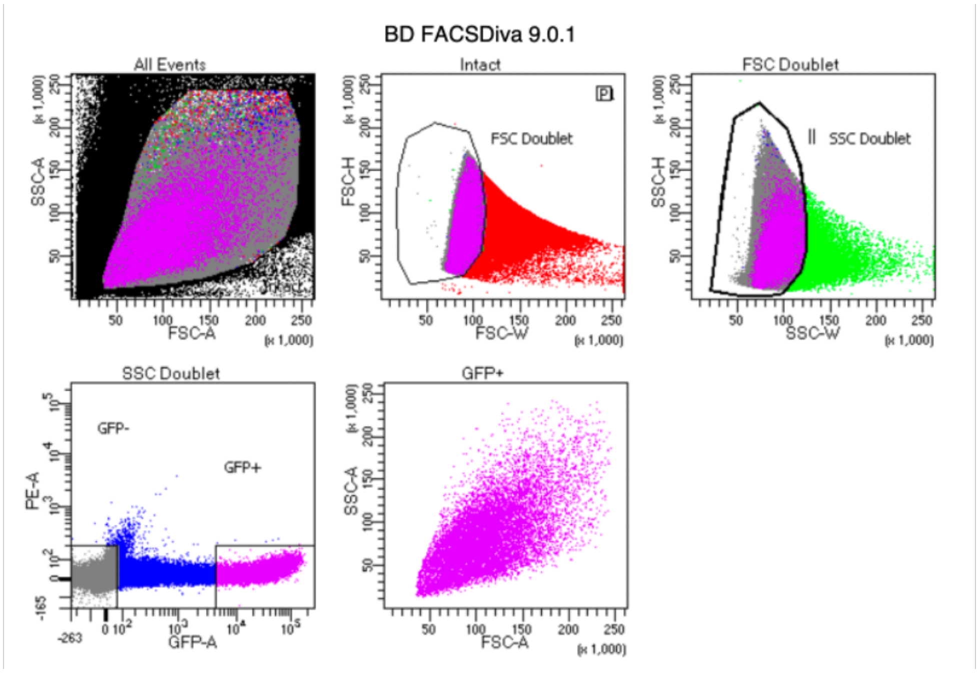

Figure S7: Flow cytometry gating strategy for isolation of GFP+ endothelial and GFP- cell populations. Representative plots illustrating the sequential gating pipeline used to purify GFP+ and GFP- cell fractions from 2 dpf Tg(*flk:GFP*) zebrafish. Initial events are displayed on forward scatter area (FSC-A) versus side scatter area (SSC-A), and the main cell population was selected to exclude debris (top, left). A two-step doublet exclusion process was applied to ensure the analysis of single cells. Cells are gated by FSC Height (FSC-H) versus Width (FSC-W) followed by SSC-H versus SSC-W. Events with disproportionately high pulse width, indicating two or more cells were excluded (top, middle and right). Single cells are separated into GFP- and GFP+ populations based on GFP fluorescence intensity, with gates defined relative to background fluorescence. FSC-A versus SSC-A plot of the gated GFP+ population, confirming a homogeneous single-cell population with expected size and granularity (bottom, right).

A

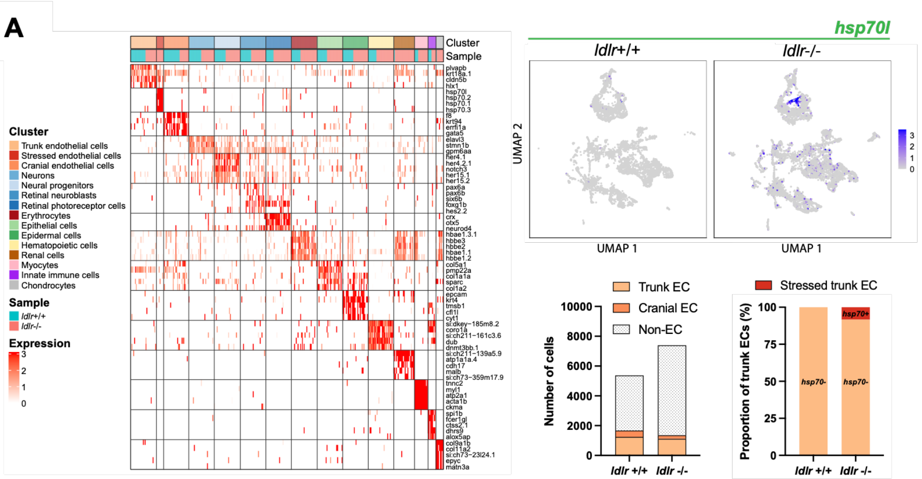

B

Gene	ldlr_avg_log2FC	ldlr_p_val_adj
hsp70l	5.30656886	2.85E-209
hsp70.1	4.07132881	6.76E-192
hsp70.2	4.76014428	2.82E-184
hsp70.3	4.90536636	2.18E-183
dnajb1b	4.30108848	1.28E-121
hsp90aa1.2	4.30001559	1.68E-61
si:ch211-248e11.2	2.41174282	1.22E-55
klf2a	2.75957271	3.36E-55
CABZ01080568.1	1.79220502	1.91E-54
fosb	2.4926953	7.27E-52
jun	2.82535348	3.81E-50
ubb	3.28571453	2.77E-48
serpinh1b	3.08616173	6.67E-46
si:ch73-335l21.4	2.85801429	1.05E-45
jund	2.58247978	9.22E-45
fgd5a	2.05509125	3.05E-44
fosab	2.53581525	1.94E-42
rsrp1	2.48171291	4.64E-42
junba	2.22479813	1.54E-40
klf2b	2.33040302	4.01E-40
kdrl	1.96929793	4.23E-37
cdh5	1.85612233	2.34E-36
creb5a	1.12831634	4.81E-35
ppp1r15a	2.20312839	2.01E-34
RALGDS	1.73069855	9.04E-34
btg2	2.16256581	2.06E-33
sele	1.93644283	2.78E-33

C

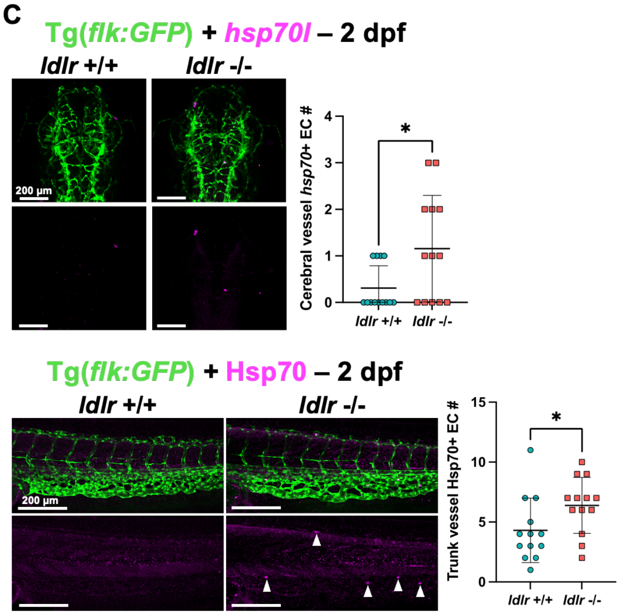

D

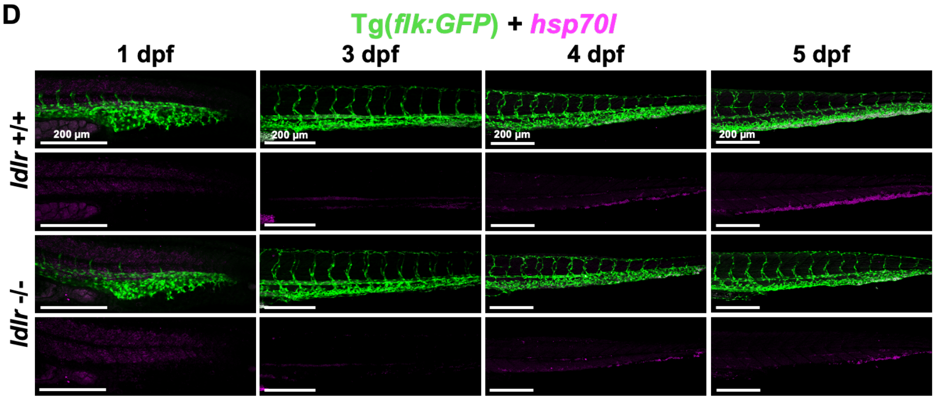

Figure S8. scRNA-seq and whole-mount staining characterization of the stressed EC subpopulation marked by *hsp70* upregulation in *ldlr*-/- zebrafish at 2 dpf. (A) Heatmap indicating the most significantly upregulated genes in each cell cluster identified between *ldlr*+/+ and *ldlr*-/- embryos (left). Uniform manifold approximation and projection (UMAP) of *hsp70l* expression enrichment between *ldlr*+/+ and *ldlr*-/- embryos in all cell populations (top, right). Quantity of trunk ECs (*hsp70*- and *hsp70*+), cranial ECs and non-ECs analyzed by scRNA-seq in *ldlr*+/+ and *ldlr*-/- embryos

(bottom, right). **(B)** Table showing top 27 most upregulated genes in the stressed *hsp70*+ endothelial cell subpopulation in *ldlr*-/- embryos, ordered by significance value. **(C)** Confocal microscopy of *hsp70l* fluorescence *in situ* RNA hybridization (FISH) stained Tg(*flk:GFP*) *ldlr*+/+ and *ldlr*-/- zebrafish cerebral vasculature at 2 dpf (top, left). Number of *hsp70*+ endothelial cells in the cerebral vasculature between *ldlr*+/+ (*n* = 13) and *ldlr*-/- (*n* = 13) zebrafish at 2 dpf; 3 independent experiments (top, right). Confocal microscopy of Hsp70 immunofluorescence stained Tg(*flk:GFP*) *ldlr*+/+ and *ldlr*-/- zebrafish trunk vasculature at 2 dpf. Arrowheads indicate Hsp70+ endothelial cells in the caudal venous plexus(bottom, left). Number of Hsp70+ endothelial cells in the trunk vasculature between *ldlr*+/+ (*n* = 13) and *ldlr*-/- (*n* = 13) zebrafish at 2 dpf; 3 independent experiments (bottom, right) . Data are mean ± s.d. Unpaired two-tailed t-test used. * $P < 0.05$. **(D)** Confocal microscopy of *hsp70l* FISH stained Tg(*flk:GFP*) *ldlr*+/+ and *ldlr*-/- zebrafish trunk vasculature at 1 dpf, 3 dpf, 4 dpf and 5 dpf. Continuous vascular *hsp70l* signal detected at 3 dpf, 4 dpf and 5 dpf is red blood cell autofluorescence. Source data are provided as a Source Data file.

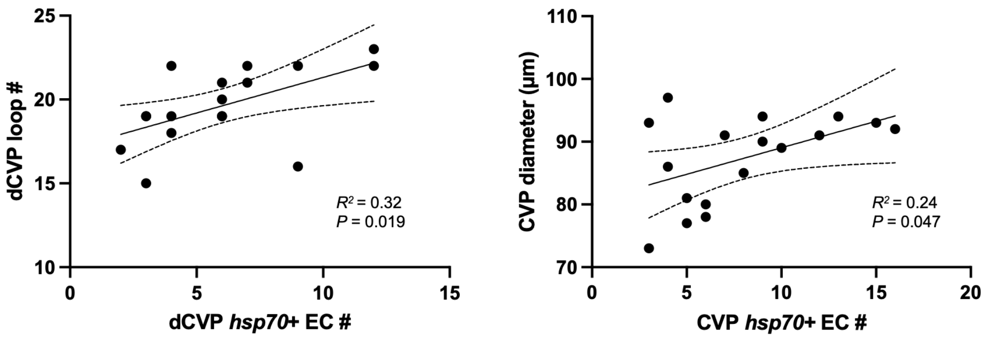

Figure S9. Correlation analysis of *hsp70*+ EC quantity and CVP morphology in *ldlr*-/- embryos at 2 dpf. Linear regression analysis showing correlation between dorsal caudal venous plexus (CVP) *hsp70*+ endothelial cell number and dorsal CVP loop number (left: R^2 = 0.3154; p = 0.019) and the correlation between CVP *hsp70*+ endothelial cell number and CVP diameter (right: R^2 = 0.2385; p = 0.0467) in *ldlr*-/- embryos (n = 17); 3 independent experiments. Mean line of best fit shown and dashed line indicates 95% confidence intervals. Source data are provided as a Source Data file.

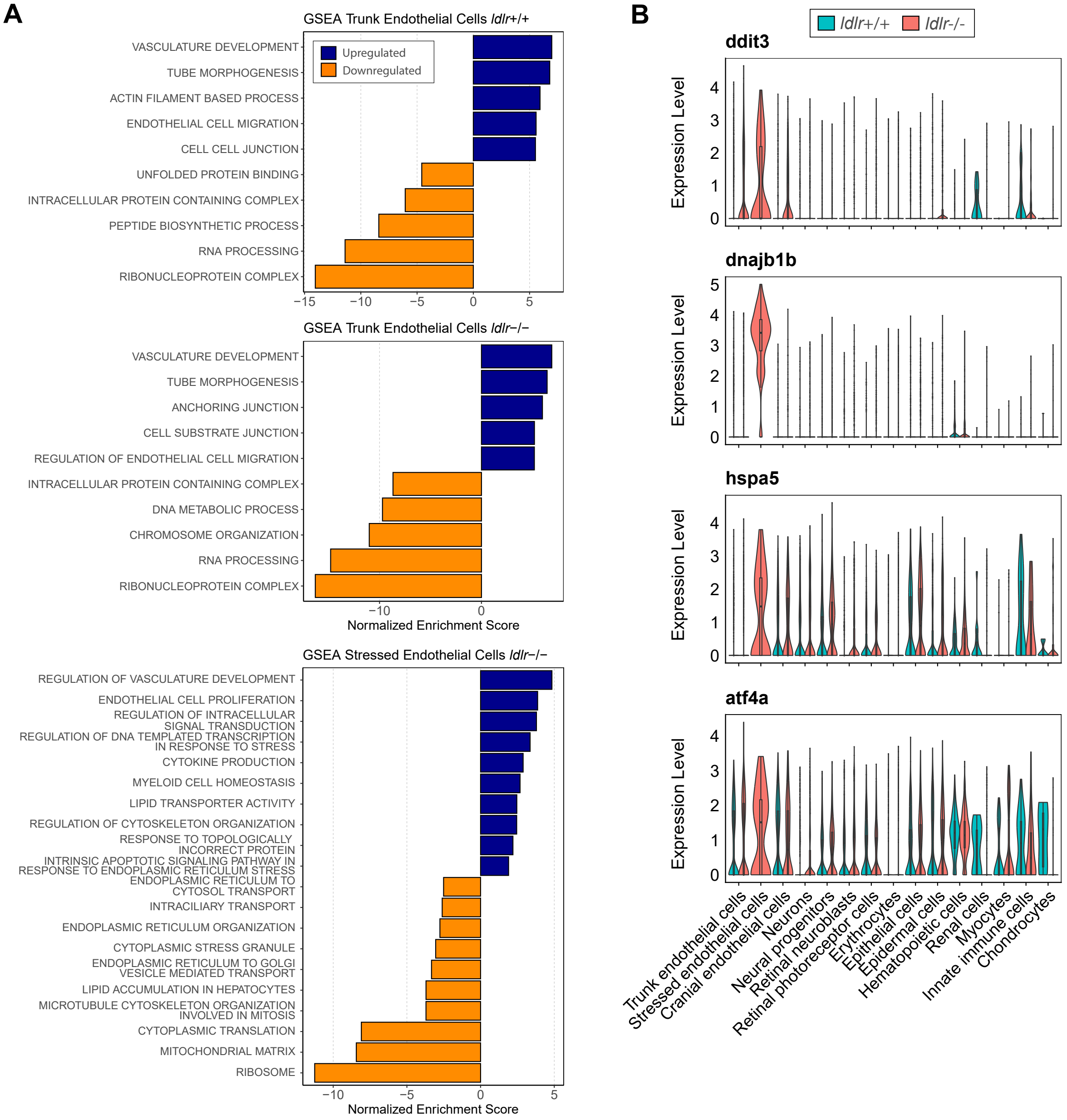

Figure S10. scRNA-seq cluster expression analysis between *ldlr*+/+ and *ldlr*-/- zebrafish under physiological flow at 2 dpf. (A) Gene set enrichment analysis (GSEA) of trunk endothelial cells in *ldlr*+/+ (top) and *ldlr*-/- (middle) embryos indicating top significantly upregulated and downregulated terms. GSEA of stressed *hsp70*+ endothelial cells in *ldlr*-/- embryos indicating significantly upregulated and downregulated terms (bottom). **(B)** Violin expression plots of cell stress response genes *ddit3*, *dnajb1b*, *hspa5* and *atf4a* across all cell populations identified in *ldlr*+/+ and *ldlr*-/- embryos.

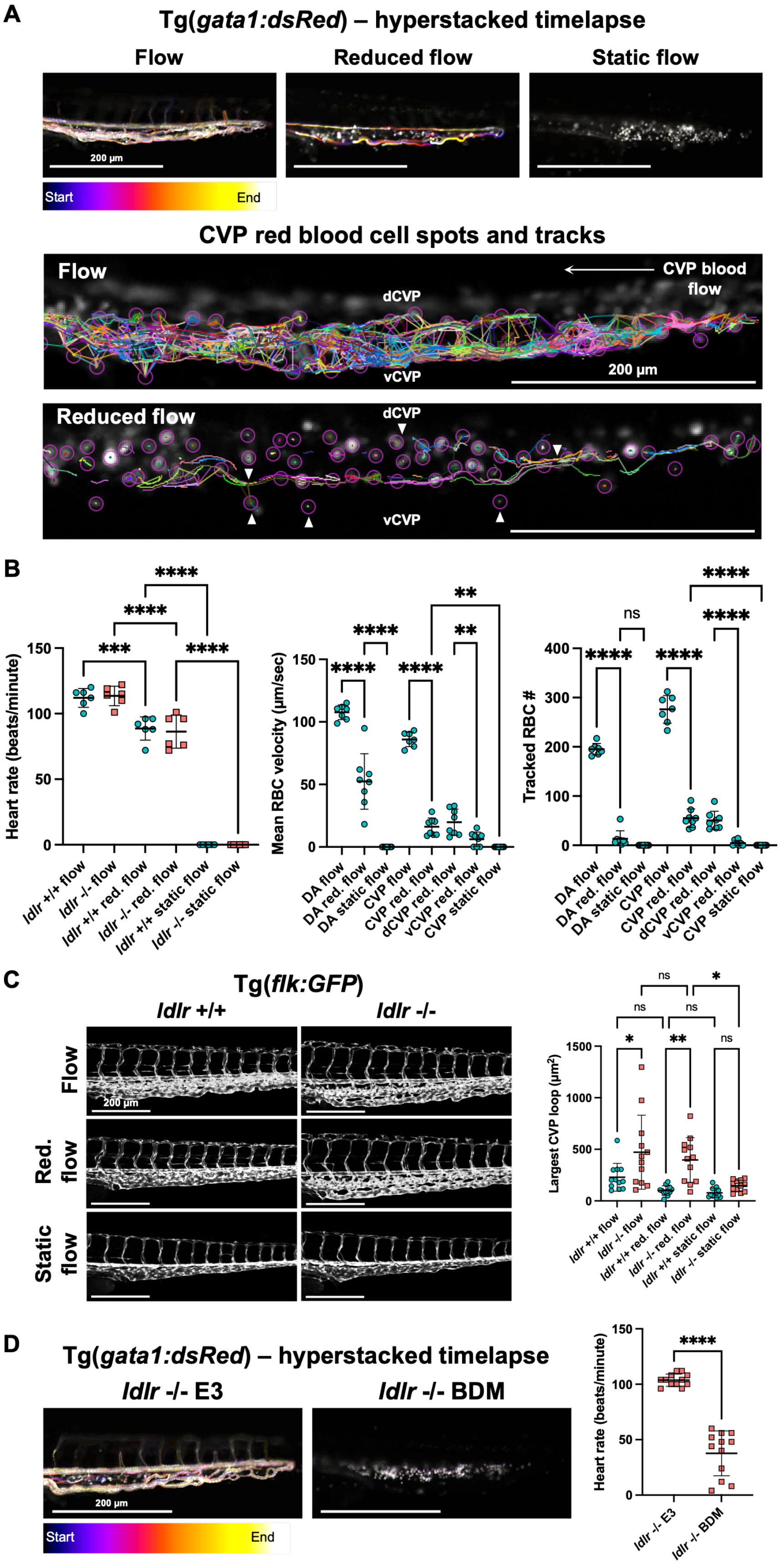
A
Tg(gata1:dsRed) – hyperstacked timelapse
Flow
Reduced flow
Static flow
200 µm
Start
End
CVP red blood cell spots and tracks
Flow
CVP blood flow
dCVP
vCVP
200 µm
Reduced flow
dCVP
vCVP
B
Heart rate (beats/minute)
ldlr +/+ flow
ldlr -/- flow
ldlr +/+ red. flow
ldlr -/- red. flow
ldlr +/+ static flow
ldlr -/- static flow
Mean RBC velocity (µm/sec)
DA flow
DA red. flow
DA static flow
CVP flow
CVP red. flow
dCVP red. flow
vCVP red. flow
CVP static flow
Tracked RBC #
ns
C
Tg(flk:GFP)
ldlr +/+
ldlr -/-
Flow
Red. flow
Static flow
200 µm
Largest CVP loop (µm²)
D
Tg(gata1:dsRed) – hyperstacked timelapse
ldlr -/- E3
ldlr -/- BDM
200 µm
Start
End
Heart rate (beats/minute)
ldlr -/- E3
ldlr -/- BDM

Figure S11. Inhibition of hemodynamics in embryonic zebrafish. (A) Effect of reduced (0.4 ng *tnnt2a* morpholino) and static (2 ng *tnnt2a* morpholino) hemodynamics in Tg(*gata1:dsRed*) zebrafish at 2 dpf. Hyperstacked timelapse of red blood cell motility in the trunk vasculature, summarizing movement routes and activity relative to timelapse period (color-coded, top). Tracking of caudal venous plexus (CVP) red blood cells (RBCs, spots) and analysis of movement routes (tracks). Red blood cell movement tracks through the dorsal CVP (dCVP) and ventral CVP (vCVP) are overlaid, with downward arrowheads indicating dCVP movement and upward arrowheads indicating vCVP movement routes in the reduced flow group (bottom). **(B)** Quantification of heart rate (left), mean red blood cell velocity (μm/sec, middle) and tracked red blood cell number (right) in *ldlr*+/+ (*n* = 6-8) and *ldlr*-/- (*n* = 6-8) zebrafish embryos exposed to physiological, reduced or static blood flow at 2 dpf. Data are mean ± s.d. Two-way ANOVA and Holm-Sidak's multiple comparison used for heart rate data and one-way ANOVA and Holm-Sidak's multiple comparison used for RBC data; 2 independent experiments.. (**C**) Confocal microscopy of trunk vasculature in *ldlr*+/+ and *ldlr*-/- Tg(*flk:GFP*) zebrafish under physiological, reduced (0.4 ng *tnnt2a* morpholino) and static (2 ng *tnnt2a* morpholino) blood flow at 2 dpf (left). Largest CVP loop area (μm^2) in *ldlr*+/+ (*n* = 12) and *ldlr*-/- (*n* = 12) zebrafish across physiological, reduced and static flow conditions at 2 dpf. Data are mean ± s.d. Two-way ANOVA and Holm-Sidak's multiple comparison used; 3 independent experiments (right). **(D)** Effect of 10mM BDM (1-2 dpf) on hemodynamics in *ldlr*-/- zebrafish embryos. Hyperstacked timelapse of red blood cell motility in the trunk vasculature, summarizing movement routes and activity relative to timelapse period (color-coded, left). Heart rate of *ldlr*-/- zebrafish embryos treated with E3 control medium (*n* = 12) or 10mM BDM (*n* = 12) at 2 dpf. Data are mean ± s.d. Paired two-tailed t-test used; 3 independent experiments. (right). **** $P < 0.0001$, *** $P < 0.001$, ** $P < 0.01$, * $P < 0.05$, ns, not significant. Source data are provided as a Source Data file.

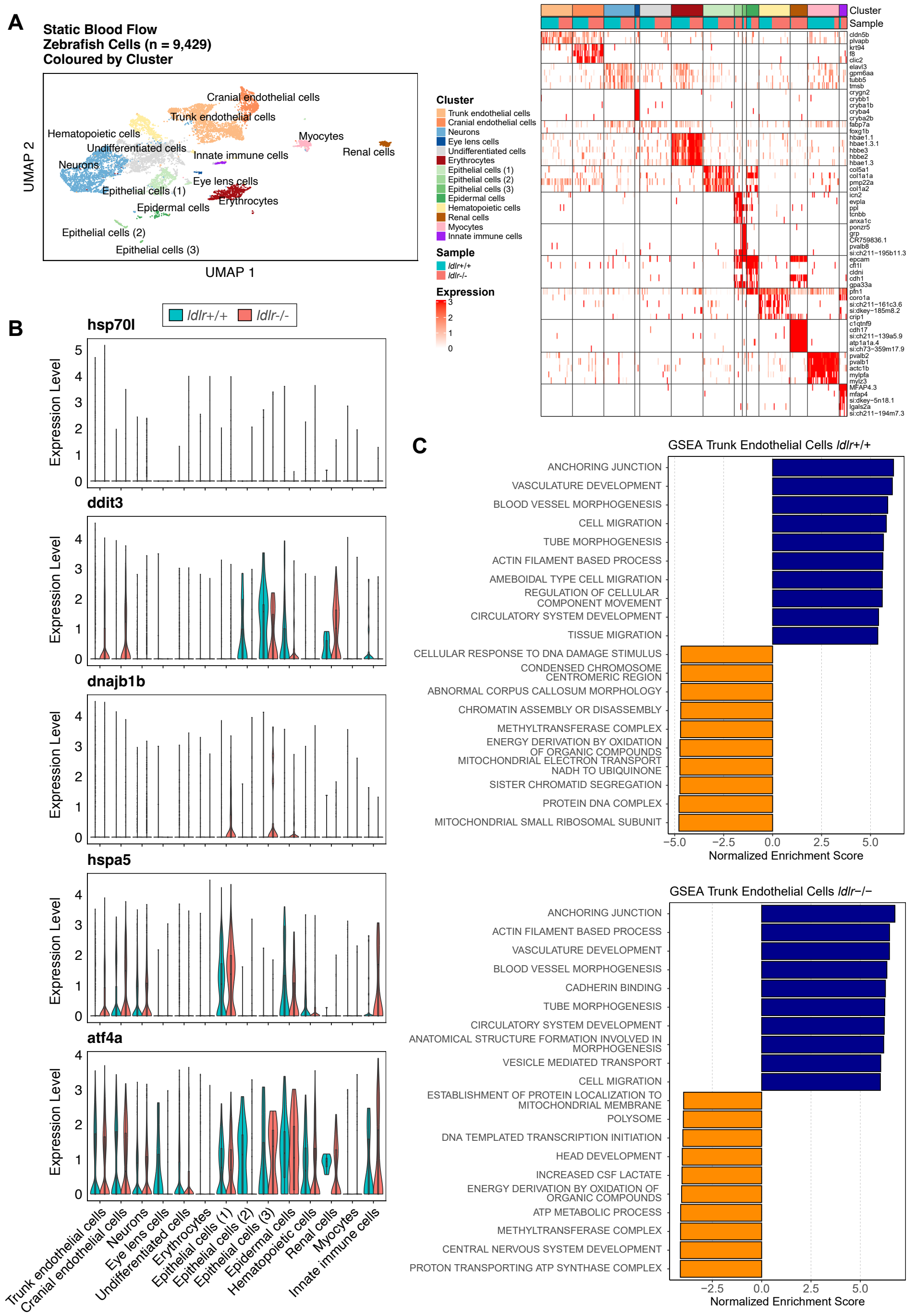

Figure S12. scRNA-seq profiling of *ldlr*+/+ and *ldlr*-/- zebrafish exposed to static blood flow (2 ng *tnnt2a* morpholino) at 2 dpf. (A) Uniform manifold approximation and projection (UMAP) showing annotated cell populations identified by scRNA-seq in embryonic zebrafish with static flow (left). Heatmap indicating the most significantly upregulated genes in each cell cluster identified between *ldlr*+/+ and *ldlr*-/- embryos with static flow (right). **(B)** Violin expression plots of cell stress response genes *hsp70l*, *ddit3*, *dnajb1b*, *hspa5* and *atf4a* across all cell populations identified in *ldlr*+/+ and *ldlr*-/- embryos exposed to static blood flow. **(C)** Gene set enrichment analysis (GSEA) of trunk endothelial cells in *ldlr*+/+ (top) and *ldlr*-/- (bottom) embryos exposed to static blood flow indicating top significantly upregulated and downregulated terms.

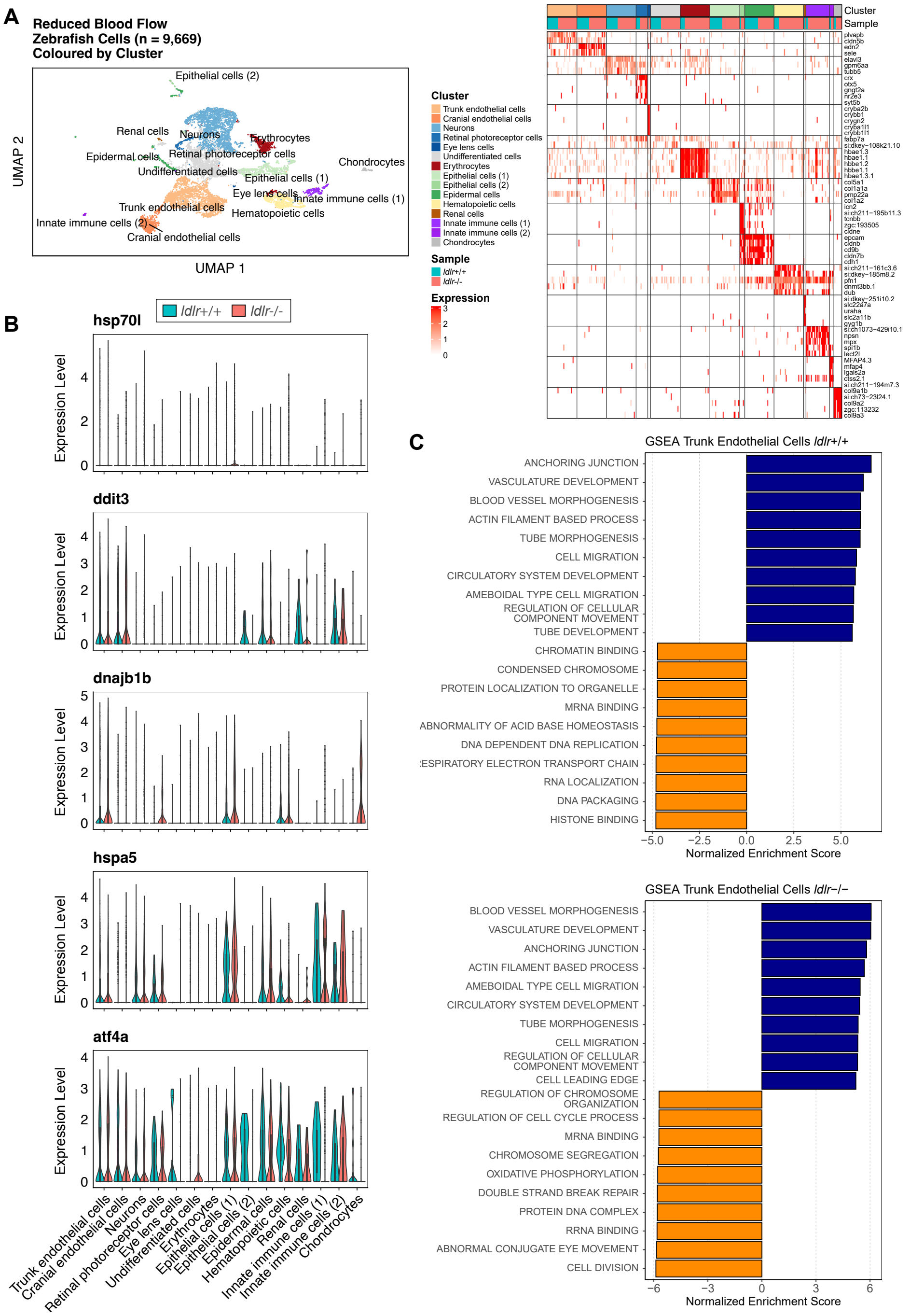

Figure S13. scRNA-seq profiling of *ldlr*+/+ and *ldlr*-/- zebrafish embryos exposed to reduced blood flow (0.4 ng *tnnt2a* morpholino) at 2 dpf. (A) Uniform manifold approximation and projection (UMAP) showing annotated cell populations identified by scRNA-seq in embryonic zebrafish with reduced flow (left). Heatmap indicating the most significantly upregulated genes in each cell cluster identified between *ldlr*+/+ and *ldlr*-/- embryos with reduced flow (right). **(B)** Violin expression plots of cell stress response genes *hsp70l*, *ddit3*, *dnajb1b*, *hspa5* and *atf4a* across all cell populations identified in *ldlr*+/+ and *ldlr*-/- embryos exposed to reduced blood flow. **(C)** Gene set enrichment analysis (GSEA) of trunk endothelial cells in *ldlr*+/+ (top) and *ldlr*-/- (bottom) embryos exposed to reduced blood flow indicating top significantly upregulated and downregulated terms.

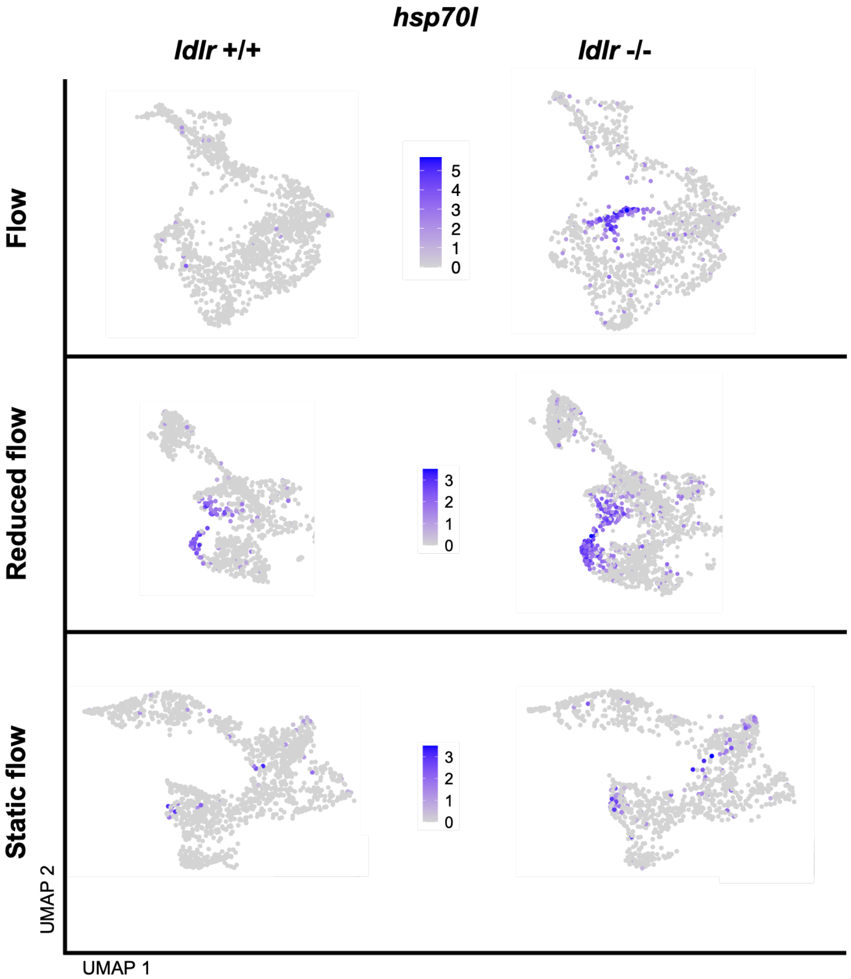

Figure S14: scRNA-seq trunk endothelial cell *hsp70l* expression profiling in *ldlr*+/+ and *ldlr*-/- zebrafish under different flow conditions at 2 dpf. Uniform manifold approximation and projection (UMAP) of *hsp70l* expression enrichment between *ldlr*+/+ and *ldlr*-/- embryos in trunk endothelial cell populations exposed to physiological, reduced (0.4 ng *tnnt2a* morpholino) and static (2 ng *tnnt2a* morpholino) hemodynamics. Expression enrichment scale indicated per flow group.

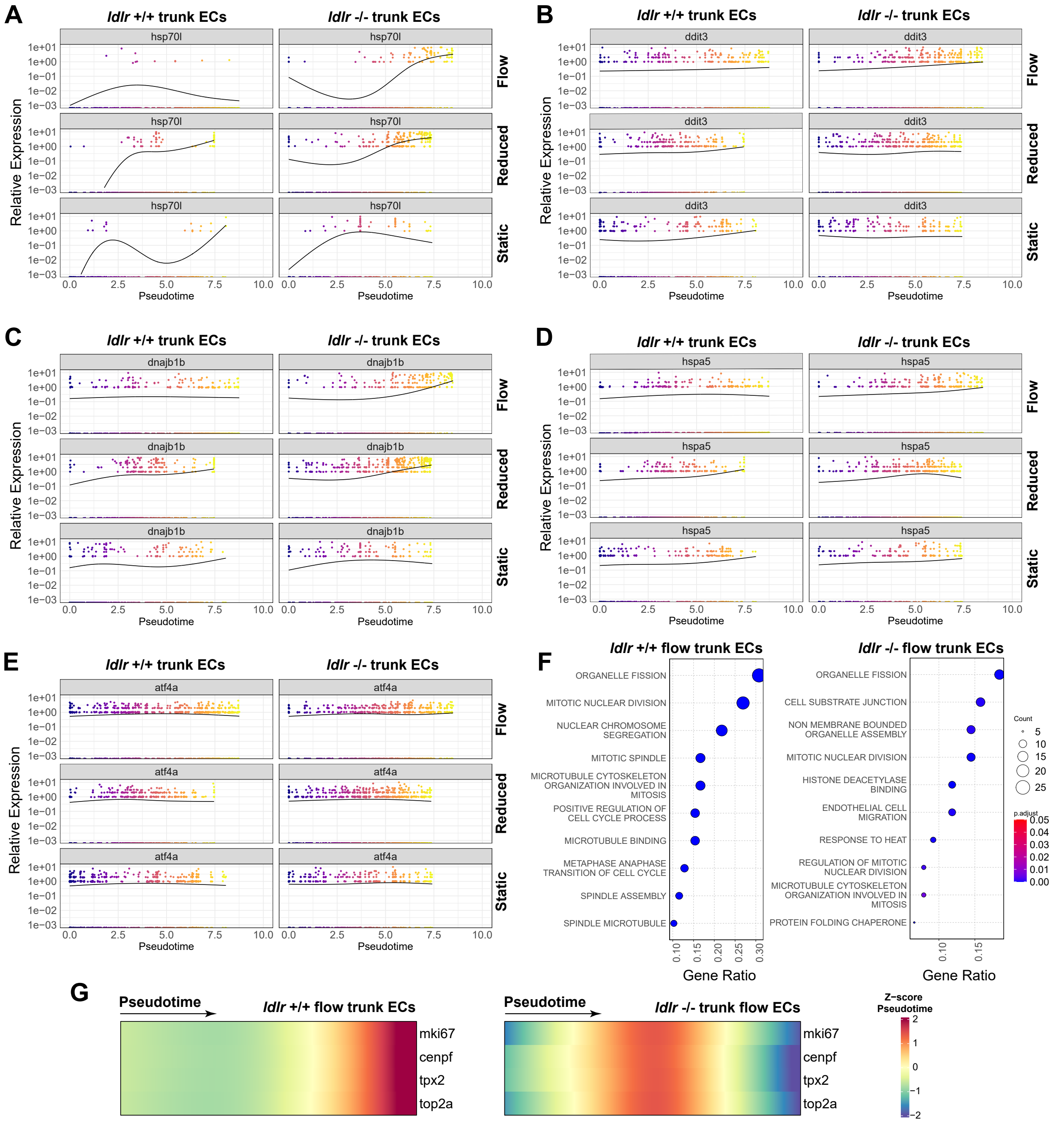

Figure S15. scRNA-seq pseudotime trajectory analysis of trunk endothelial cells between *ldlr*+/+ and *ldlr*-/- zebrafish under different flow conditions at 2 dpf. Expression of *hsp70l* **(A)**, *ddit3* **(B)**, *dnajb1b* **(C)**, *hspa5* **(D)**, and *atf4a* **(E)** across pseudotime in *ldlr*+/+ (left) or *ldlr*-/- (right) trunk endothelial cells with physiological (top), reduced (0.4 ng *tnnt2a* morpholino, middle) or static (2 ng *tnnt2a* morpholino, bottom) blood flow. **(F)** Gene ontology analysis of differentially expressed pseudotime genes in *ldlr*+/+ (left) or *ldlr*-/- (right) trunk endothelial cells exposed to physiological blood flow. **(G)** Pseudotime z-score heatmap plot of genes involved in organelle fission, mitotic nuclear division and microtubule cytoskeleton organization (identified by pseudotime differential expression gene ontology analysis): *mki67*, *cenpf*, *tpx2* and *top2a* in *ldlr*+/+ (left) and *ldlr*-/- (right) zebrafish trunk endothelial cells under physiological blood flow.

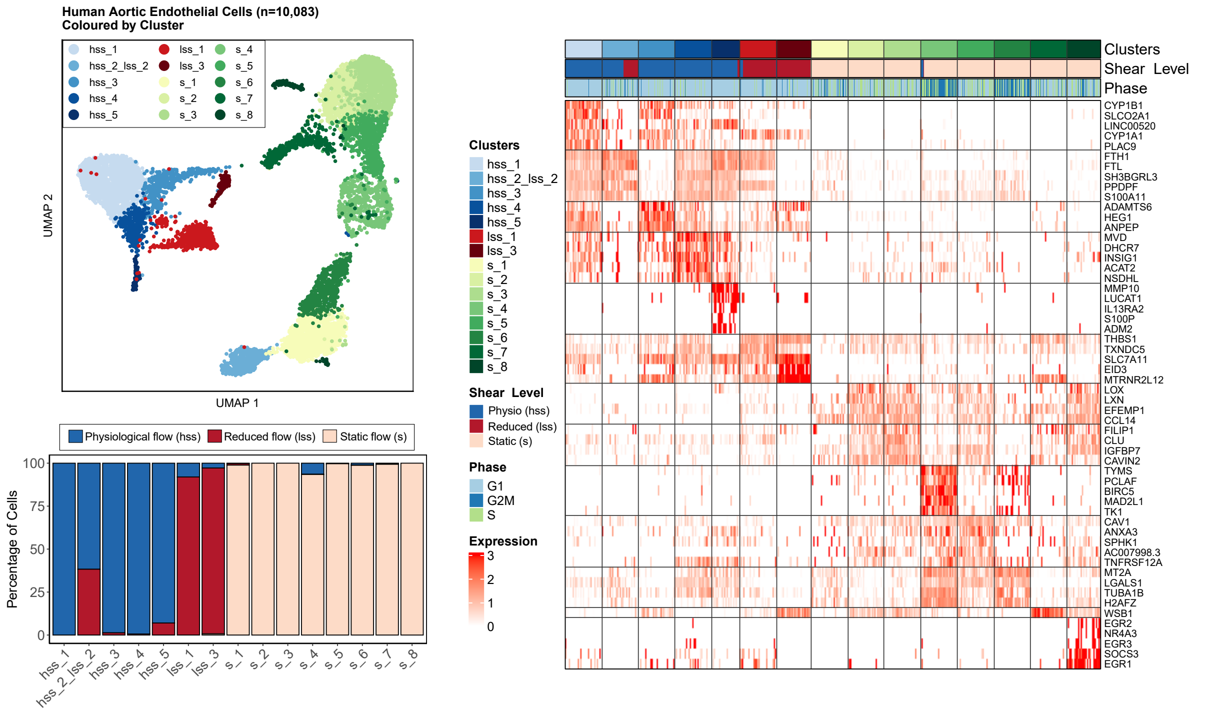

Figure S16. scRNA-seq cluster expression analysis of human aortic endothelial cells (HAECs) exposed to 30 dynes/cm^2 physiological flow (high shear stress, hss), 2.5 dynes/cm^2 reduced flow (low shear stress, lss) or 0 dynes/cm^2 static flow (s). Uniform manifold approximation and projection (UMAP) of HAEC subpopulations identified following exposure to hss, lss or static flow (top, left). Percentage of HAECs exposed to hss, lss or static flow in each subpopulation cluster identified (bottom, left). Heatmap indicating the most significantly upregulated genes in each flow-modulated HAEC cluster identified (right).

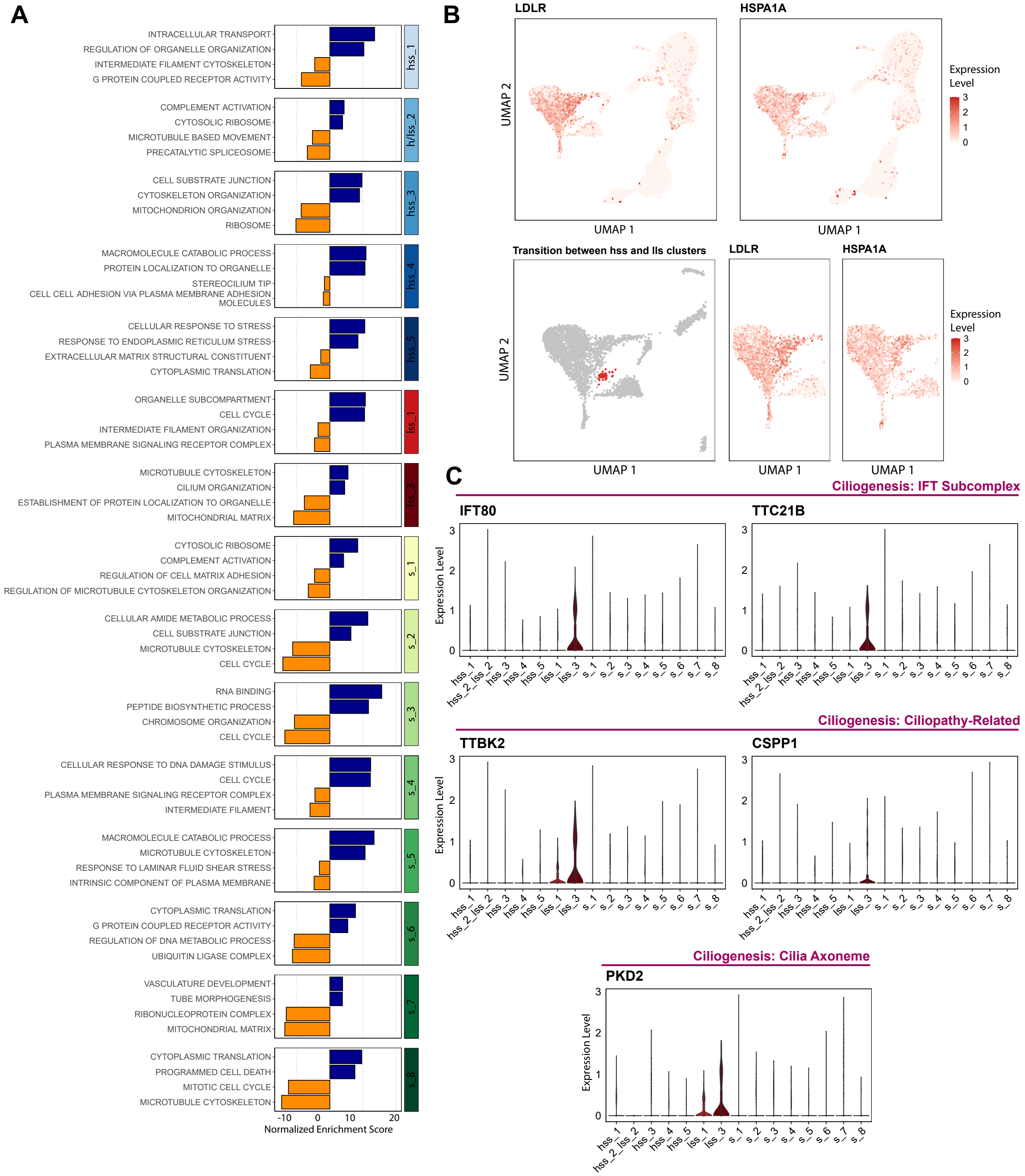

Figure S17. scRNA-seq profiling of flow-modulated HAECs. (A) Gene set enrichment analysis (GSEA) summary of significantly upregulated and downregulated terms for each flow-modulated HAEC subpopulation identified following exposure to hss, lss or static flow. **(B)** Uniform manifold approximation and projection (UMAP) of *LDLR* and *HSPA1A* expression enrichment in all HAECs (top). UMAP highlighting HAECs within the transition zone between hss and lss cell clusters (bottom, left). UMAP of *LDLR* and *HSPA1A* expression enrichment in HAEC clusters hss_1, hss/lss_2, hss_3, hss_4, hss_5 and lss_1 (bottom, right). **(C)**. Violin expression plot of ciliogenesis genes *IFT80*, *TTC21B*, *TTBK2*, *CSPP1* and *PKD2* in all flow-modulated HAEC subpopulations identified following exposure to hss, lss or static flow.

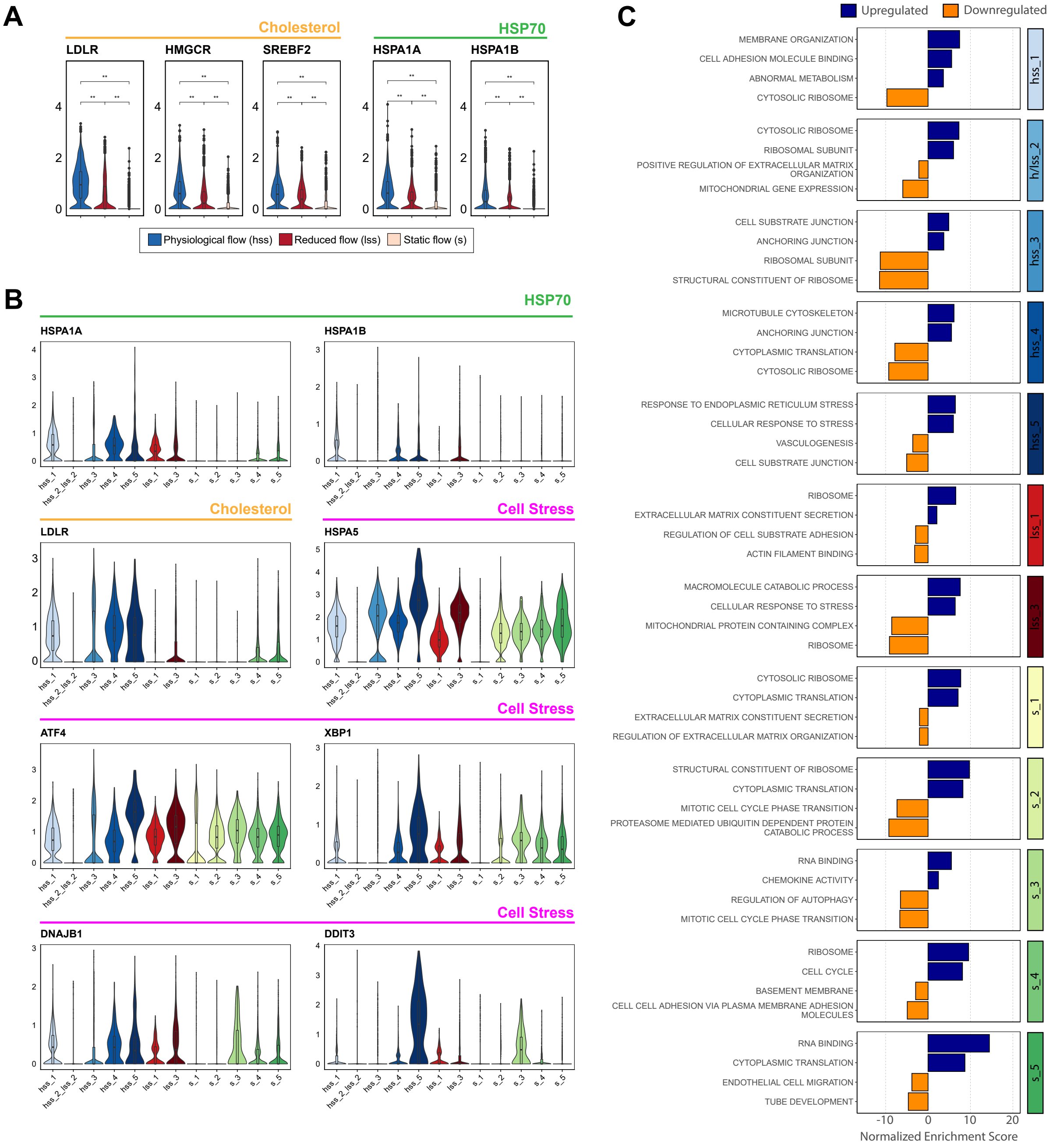

Figure S18. scRNA-seq analysis of integrated flow-modulated HAEC datasets exposed to physiological (hss), reduced (lss) or static (s) flow. (A) Violin expression plots for *LDLR*, *HMGCR*, *SREB2*, *HSPA1A* and *HSPA1B* and statistical comparisons between integrated physiological (hss), reduced (lss) or static (s) flow exposed HAECs. Two-tailed Wilcoxon rank sum text and Bonferroni's multiple comparison test used. ** $P < 0.01$. **(B)** Violin expression plots for *HSPA1A, HSPA1B, LDLR, HSPA5, ATF4, XBP1, DNAJB1 and DDIT3* in all integrated flow-modulated HAEC subpopulations identified. **(C)** Gene set enrichment analysis (GSEA) summary of significantly upregulated and downregulated terms for each integrated flow-modulated HAEC subpopulation identified.

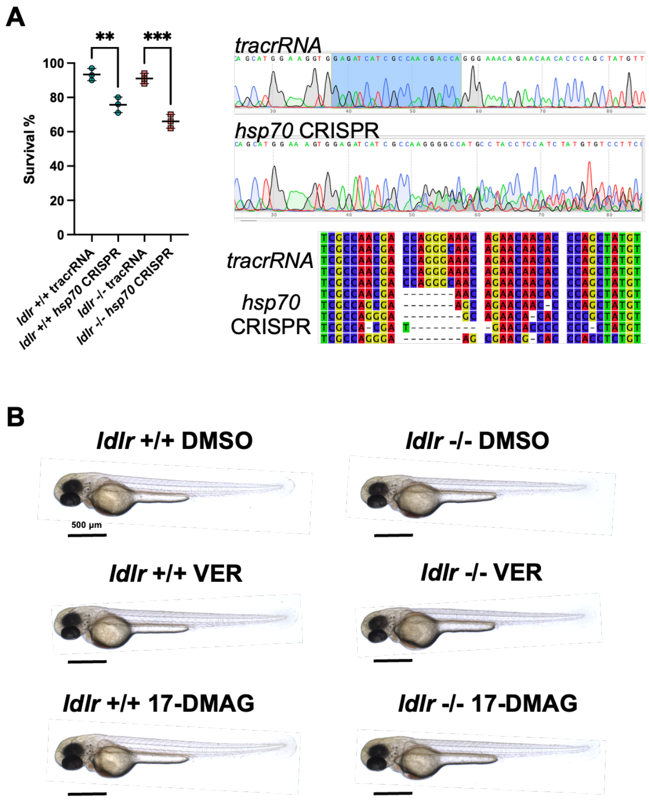

Figure S19. Effect of *hsp70* modulation during zebrafish development. (A) Survival (%) following acute CRISPR/Cas9 knockout of *hsp70* or control tracRNA injection in *ldlr*+/+ and *ldlr*-/- zebrafish at 2 dpf. Average survival (%) of embryos calculated per injected clutch (n = 36) and are represented as mean ± s.d. One-way ANOVA and Holm-Sidak's multiple comparison used; 4 independent experiments. *** $P < 0.001$, ** $P < 0.01$ (left). DNA sequencing showing the target site in exon 1 of the *hsp70l* locus. The *hsp70* gRNA sequence prior to the PAM region (GGG) is highlighted in the tracRNA control group (top, right). TracRNA injected zebrafish do not show any deletions, whereas all CRISPR/Cas9 injected zebrafish display deletions at the target site (bottom, right). **(B)** Brightfield microscopy of *ldlr*+/+ and *ldlr*-/- zebrafish treated (1-2 dpf) with 50 μM VER-155008, 50 μM 17-DMAG or DMSO control at 2 dpf. Source data are provided as a Source Data file.

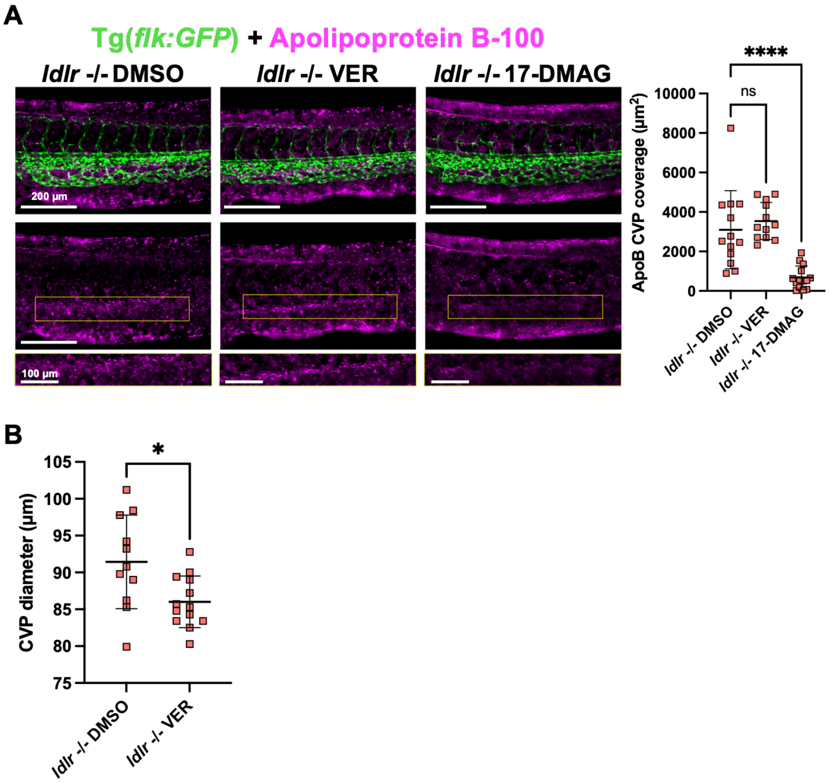

Figure S20. Effect of *hsp70* modulation on CVP ApoB coverage and CVP diameter in *ldlr*-/- zebrafish. (A) Confocal microscopy of ApoB stained Tg(*flk:GFP*) *ldlr*-/- zebrafish following treatment with 50 µM VER-155008, 50 µM 17-DMAG or DMSO control at 2 dpf (left). Area of ApoB coverage in the caudal venous plexus (CVP) *ldlr*-/- zebrafish treated with 50 µM VER-155008 (n = 12), 50 µM 17-DMAG (n = 14) or DMSO control (n = 13) at 2 dpf. Boxed region indicates CVP area used for measurements. Inset of CVP ApoB coverage indicated. Data are mean ± s.d. One-way ANOVA and Holm-Sidak's multiple comparison used; 3 independent experiments (right). **(B)** CVP diameter (µm) in *ldlr*-/- zebrafish following treatment with 50 µM VER-155008 (n = 13) or DMSO control (n = 11) at 2 dpf. Paired two-tailed t-test used; 3 independent experiments. **** $P < 0.0001$, * $P < 0.05$, ns, not significant. Source data are provided as a Source Data file.

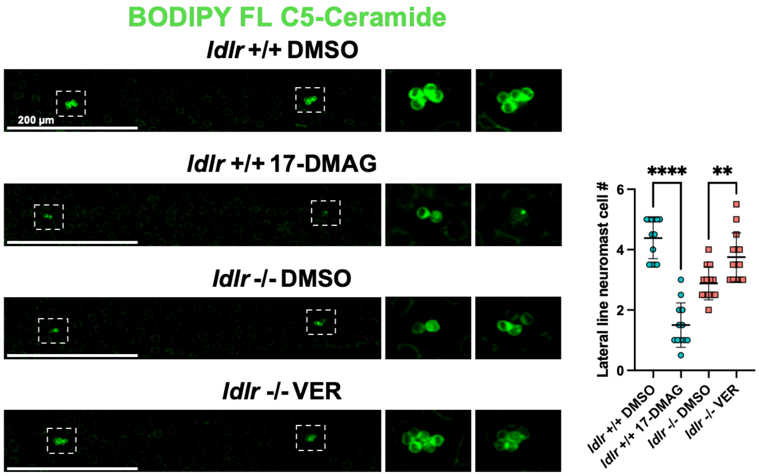

Figure S21. Effect of *hsp70* modulation on neuromast development in embryonic zebrafish. Confocal microscopy of BODIPY FL C5-Ceramide-stained lateral line neuromasts in *ldlr*+/+ or *ldlr*-/- zebrafish following treatment with 50 µM 17-DMAG, 50 µM VER-155008 or DMSO control at 2 dpf (left). Average number of (BODIPY FL C5-Ceramide stained) lateral line neuromast cells per neuromast in *ldlr*+/+ or *ldlr*-/- zebrafish treated with 50 µM 17-DMAG (n = 12), 50 µM VER-155008 (n = 14) or DMSO (n = 13) at 2 dpf. Data are mean ± s.d. One-way ANOVA and Holm-Sidak's multiple comparison used; 3 independent experiments. **** $P < 0.0001$, ** $P < 0.01$ (right). Source data are provided as a Source Data file.

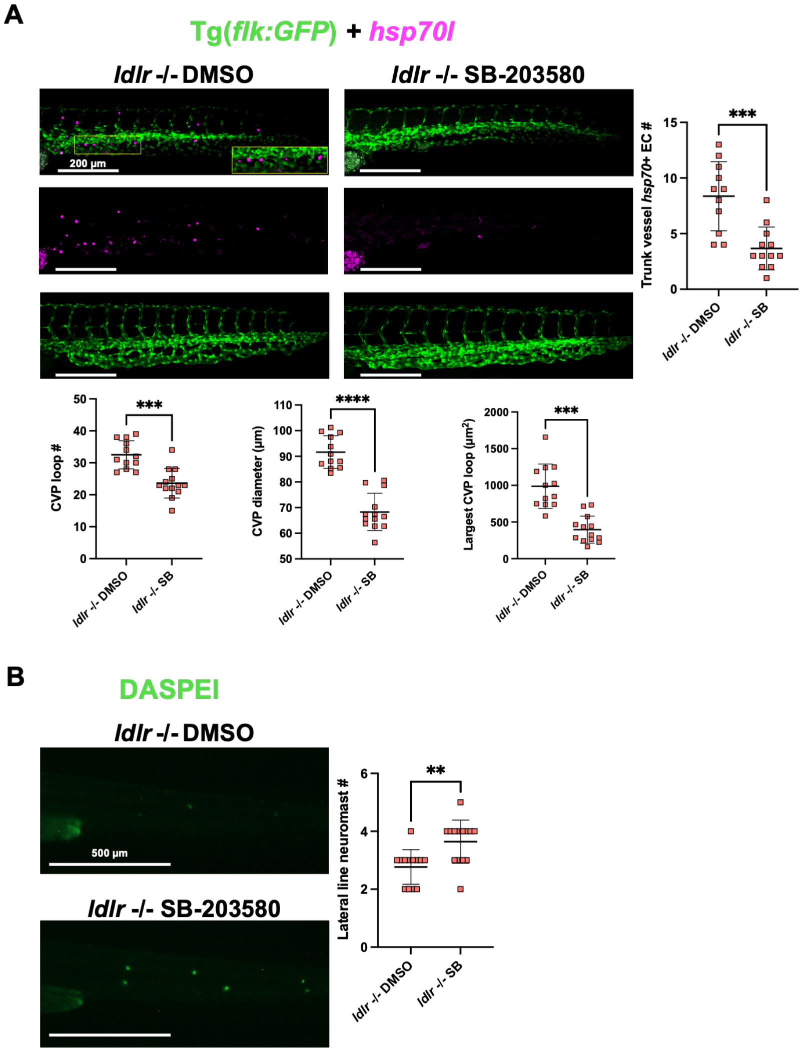

Figure S22. Effect of p38 Mapk signaling inhibition on *hsp70*+ EC quantity and CVP morphology in *ldlr*-/- zebrafish. (A) Confocal microscopy of *hsp70l* FISH stained Tg(*flk:GFP*) *ldlr*-/- zebrafish trunk vasculature following treatment with 100 µM SB-203580 (p38 Mapk inhibitor) or DMSO control at 2 dpf. Boxed region shows inset of caudal venous plexus (CVP) *hsp70*+ endothelial cells (top, left). Total number of *hsp70*+ endothelial cells in the trunk vasculature between *ldlr*-/- DMSO control (*n* = 11) and *ldlr*-/- 100 µM SB-203580 (*n* = 12) treated zebrafish at 2 dpf (top, right). CVP remodeling metrics: loop number, CVP diameter (µm) and loop area (µm^2) in *ldlr*-/- zebrafish treated with 100 µM SB-203580 (*n* = 13) or DMSO (*n* = 12) at 2 dpf. Data are mean ± s.d. Paired two-tailed t-test used; 3 independent experiments. **(B)** Epifluorescence microscopy of DASPEI stained neuromasts in *ldlr*-/- zebrafish following treatment with 100 µM SB-203580 or DMSO control at 2 dpf (left). Number of (DASPEI-stained) posterior lateral line neuromasts in *ldlr*-/- zebrafish treated with 100 µM SB-203580 (*n* = 14) or DMSO control (*n* = 13) at 2 dpf (right). Data are mean ± s.d. Paired two-tailed t-test used; 3 independent experiments (right). **** $P < 0.0001$, *** $P < 0.001$, ** $P < 0.01$. Source data are provided as a Source Data file.

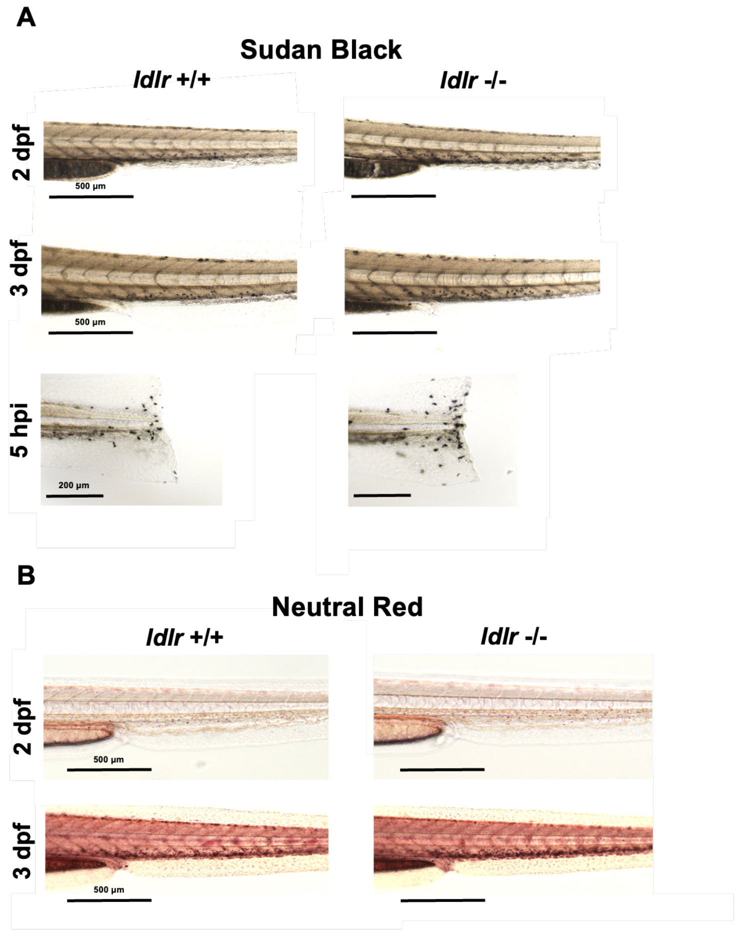

Figure S23. Sudan black and Neutral Red staining in *ldlr*-/- zebrafish embryos and larvae. (A) Representative brightfield microscopy of Sudan black stained (neutrophils) in the caudal hematopoietic tissue (CHT) of *ldlr*+/+ (left) and *ldlr*-/- (right) zebrafish at 2 dpf (top), 3 dpf (middle) and tail epithelium at 5 hours post injury (hpi) following standard tail fin resection (bottom). **(B)** Representative brightfield microscopy of Neutral Red stained (macrophages) in the CHT of in *ldlr*+/+ (left) and *ldlr*-/- (right) zebrafish at 2 dpf (top) and 3 dpf (bottom).

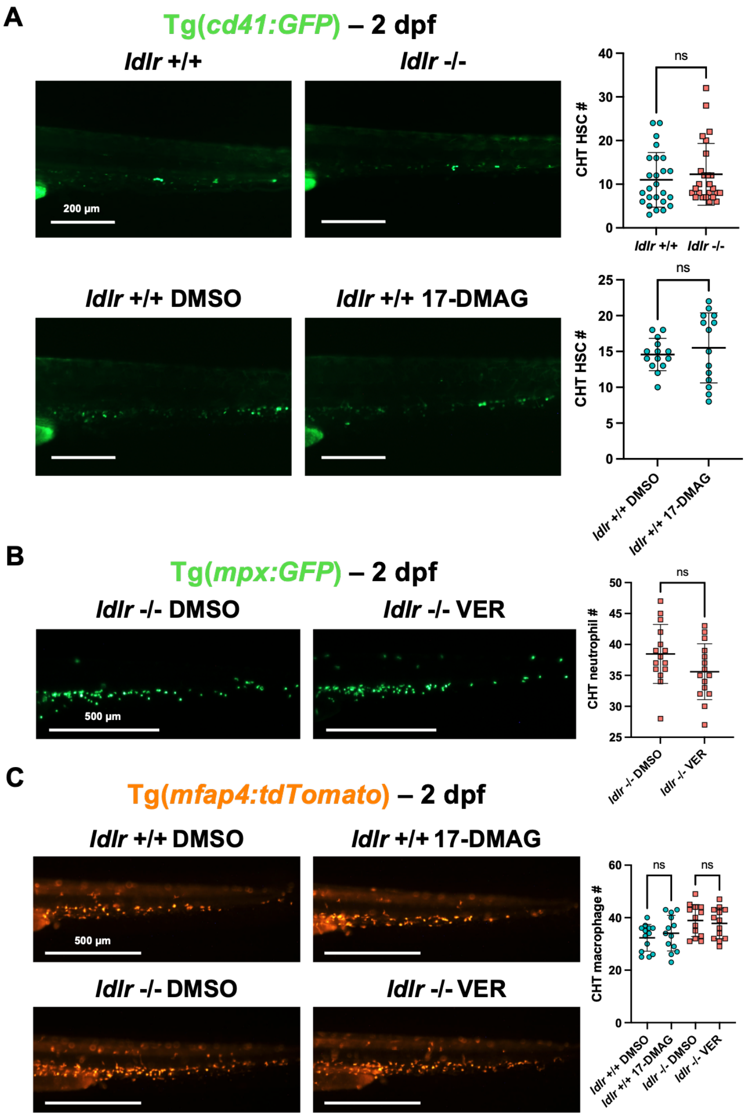

Figure S24. Hematopoietic and myeloid cell phenotypes during development and following *hsp70* modulation in *ldlr-/-* zebrafish. (A) Epifluorescence microscopy of the CHT in Tg(*cd41:GFP*) *ldlr*+/+ and *ldlr*-/- zebrafish at 2 dpf. Number of HSCs (*cd41:GFP*^{low} expressing cells) in the CHT of *ldlr*+/+ (*n* = 25) and *ldlr*-/- (*n* = 25) zebrafish at 2 dpf. Data are mean ± s.d. Unpaired two-tailed t-test used; 4 independent experiments (top). Epifluorescence microscopy of the CHT in Tg(*cd41:GFP*) *ldlr*+/+ zebrafish following treatment with 50 µM 17-DMAG or DMSO control at 2 dpf. Number of HSCs (*cd41:GFP*^{low} expressing cells) in the CHT of *ldlr*+/+ zebrafish treated with 50 µM 17-DMAG (*n* = 14) or DMSO control (*n* = 14) at 2 dpf. Data are mean ± s.d. Paired two-tailed t-test used; 3 independent experiments (bottom). **(B)** Epifluorescence microscopy of the CHT in Tg(*mpx:GFP*) *ldlr*-/- zebrafish following treatment with 50 µM VER-155008 or DMSO control at 2 dpf (left). Number of CHT neutrophils in *ldlr*-/- zebrafish treated with 50 µM VER-155008 (*n* = 15) or DMSO control (*n* = 15) at 2 dpf. Data are mean ± s.d. Unpaired two-tailed t-test used; 3 independent experiments (right). **(C)** Epifluorescence microscopy of the CHT in Tg(*mfap4:tdTomato*) *ldlr*+/+ zebrafish following treatment with 50 µM 17-DMAG or DMSO control at 2 dpf (top) and *ldlr*-/- zebrafish following treatment with 50 µM VER-155008 or DMSO control at 2 dpf (bottom). Number of CHT macrophages in *ldlr*+/+ and *ldlr*-/- zebrafish treated with 50 µM 17-DMAG (*n* = 13), 50 µM VER-155008 (*n* = 13) or DMSO control (*n* = 13) at 2 dpf. Data are mean ± s.d. One-way ANOVA and Holm-Sidak's multiple comparison used; 3 independent experiments (right). *** $P < 0.001$, * $P < 0.05$, ns, not significant. Source data are provided as a Source Data file.

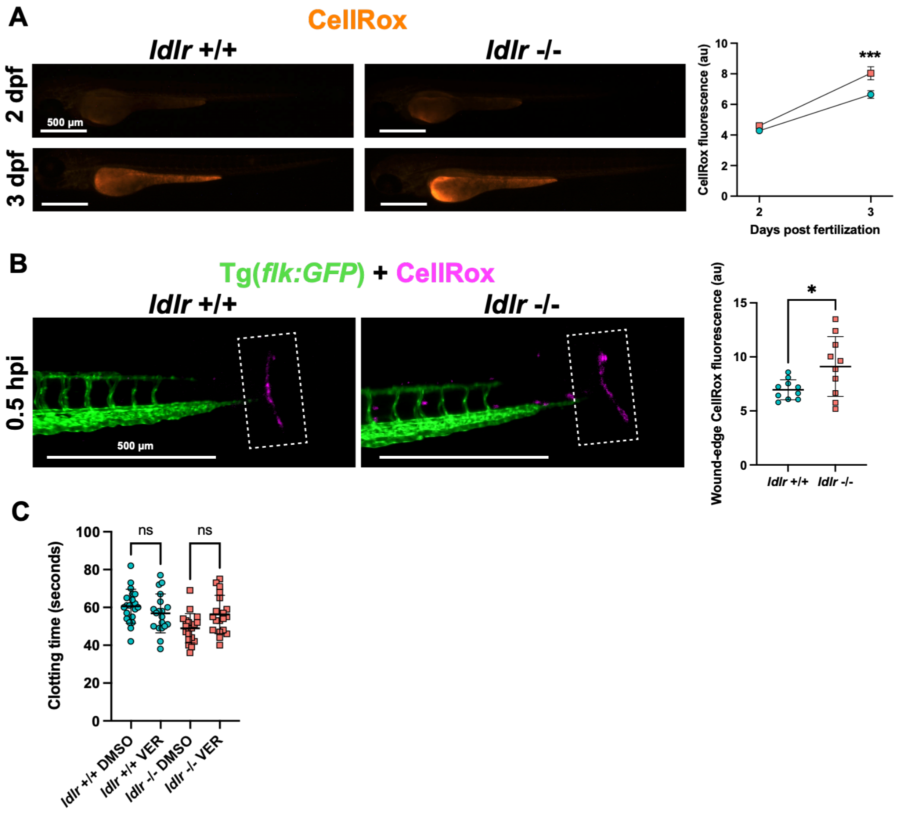

Figure S25. Oxidative stress levels during development and following *hsp70* modulation and/or injury in *ldlr*-/- zebrafish. (A) Epifluorescence microscopy of CellRox Orange stained *ldlr*+/+ and *ldlr*-/- zebrafish at 2 dpf and 3 dpf (left). Whole body CellRox fluorescence intensity in *ldlr*+/+ (*n* = 32) and *ldlr*-/- (*n* = 34) zebrafish at 2 dpf and 3 dpf. Data are mean ± s.e.m. Two-way ANOVA and Holm-Sidak's multiple comparison used; 5 independent experiments. Au, arbitrary units (right). **(B)** Epifluorescence microscopy of the tail fin wound-edge in 3 dpf Tg(*flk:GFP*) *ldlr*+/+ and *ldlr*-/- zebrafish following epithelial resection and CellRox orange staining at 0.5 hours post injury (hpi, left). Wound-edge CellRox fluorescence intensity in *ldlr*+/+ (*n* = 10) and *ldlr*-/- (*n* = 10) zebrafish following tail fin epithelial resection at 0.5 hpi. Data are mean ± s.d. Unpaired two-tailed t-test used; 2 independent experiments (right). **(C)** Time to thrombosis (clotting time in seconds) following $FeCl_3$ exposure in *ldlr*+/+ and *ldlr*-/- zebrafish treated with 50 µM VER-155008 (*n* = 19) or DMSO control (*n* = 19-20). Data are mean ± s.d. One-way ANOVA and Holm-Sidak's multiple comparison used; 3 independent experiments. *** $P < 0.001$, * $P < 0.05$, ns, not significant. Source data are provided as a Source Data file.
