# Supplementary material for: Flow-mediated endothelial remodeling and inflammation drive developmental vascular susceptibility in ldlr loss of function
Source: Nat Commun. 2026 May 21;17:6926. doi: 10.1038/s41467-026-72756-3 (PMC13388945; doi:10.1038/s41467-026-72756-3)
Supplement: Supplementary file 3 — Description of Additional Supplementary Files [file 41467_2026_72756_MOESM3_ESM.pdf]

Description of Additional Supplementary Files

File Name: Supplementary Data 1
Description: Supplementary data files listing significantly enriched genes for each annotated zebrafish cluster, including log_2 fold change values and adjusted p values. Cell type annotation was performed based on gene ranking by log_2 fold change and statistical significance.

File Name: Supplementary Movie 1
Description: Red blood cell flow through ldlr+/+ trunk vasculature at 2 dpf visualized by epifluorescence microscopy in Tg(gata1:dsRed) zebrafish.

File Name: Supplementary Movie 2
Description: Red blood cell flow through ldlr+/+ trunk vasculature at 5 dpf visualized by epifluorescence microscopy in Tg(gata1:dsRed) zebrafish.

File Name: Supplementary Movie 3
Description: Red blood cell flow through ldlr-/- trunk vasculature at 2 dpf visualized by epifluorescence microscopy in Tg(gata1:dsRed) zebrafish.

File Name: Supplementary Movie 4
Description: Red blood cell flow through the trunk vasculature visualized by epifluorescence microscopy of Tg(gata1:dsRed) 2 dpf zebrafish. Embryo was injected with control morpholino and displays physiological heart contractility and blood flow.

File Name: Supplementary Movie 5
Description: Red blood cell flow through the trunk vasculature visualized by epifluorescence microscopy of Tg(gata1:dsRed) 2 dpf zebrafish. Embryo was injected with 2 ng of tnnt2a morpholino and displays arrested heart contractility and static blood flow.

File Name: Supplementary Movie 6
Description: Red blood cell flow through the trunk vasculature visualized by epifluorescence microscopy of Tg(gata1:dsRed) 2 dpf zebrafish. Embryo was injected with 0.4 ng of tnnt2a morpholino and displays reduced heart contractility and blood flow.

File Name: Supplementary Movie 7
Description: Red blood cell flow through the trunk vasculature visualized by epifluorescence microscopy of Tg(gata1:dsRed) 2 dpf zebrafish. Embryo was incubated in control E3 medium and displays physiological heart contractility and blood flow.

File Name: Supplementary Movie 8
Description: Red blood cell flow through the trunk vasculature visualized by epifluorescence microscopy of Tg(gata1:dsRed) 2 dpf zebrafish. Embryo was incubated in 10mM BDM and displays diminished heart contractility and blood flow.

File Name: Supplementary Movie 9
Description: Red blood cell flow through the tail fin vasculature visualized by brightfield microscopy of 4 days post tailfin amputation ldlr+/+ zebrafish. Erythrocyte movement through the regenerated caudal vascular loop is observed.

File Name: Supplementary Movie 10
Description: Red blood cell flow through the tail fin vasculature visualized by brightfield microscopy of 4 days post tailfin amputation ldlr-/- zebrafish. Erythrocytes are unable to move through the caudal vascular loop due to diminished vessel regeneration.
