# Supplementary material for: Flow-mediated endothelial remodeling and inflammation drive developmental vascular susceptibility in ldlr loss of function
Source: Nat Commun. 2026 May 21;17:6926. doi: 10.1038/s41467-026-72756-3 (PMC13388945; doi:10.1038/s41467-026-72756-3)
Supplement: Supplementary file 6 — Reporting Summary [file 41467_2026_72756_MOESM6_ESM.pdf]

nature portfolio

Corresponding author(s): Aryan Kaveh and Calum A MacRae

Last updated by author(s): Apr 19, 2026

Reporting Summary

Nature Portfolio wishes to improve the reproducibility of the work that we publish. This form provides structure for consistency and transparency in reporting. For further information on Nature Portfolio policies, see our Editorial Policies and the Editorial Policy Checklist.

Statistics

For all statistical analyses, confirm that the following items are present in the figure legend, table legend, main text, or Methods section.

n/a	Confirmed	
☐	☒	The exact sample size (*n*) for each experimental group/condition, given as a discrete number and unit of measurement
☐	☒	A statement on whether measurements were taken from distinct samples or whether the same sample was measured repeatedly
☐	☒	The statistical test(s) used AND whether they are one- or two-sided<br>*Only common tests should be described solely by name; describe more complex techniques in the Methods section.*
☒	☐	A description of all covariates tested
☐	☒	A description of any assumptions or corrections, such as tests of normality and adjustment for multiple comparisons
☐	☒	A full description of the statistical parameters including central tendency (e.g. means) or other basic estimates (e.g. regression coefficient) AND variation (e.g. standard deviation) or associated estimates of uncertainty (e.g. confidence intervals)
☐	☒	For null hypothesis testing, the test statistic (e.g. *F*, *t*, *r*) with confidence intervals, effect sizes, degrees of freedom and *P* value noted<br>*Give P values as exact values whenever suitable.*
☒	☐	For Bayesian analysis, information on the choice of priors and Markov chain Monte Carlo settings
☒	☐	For hierarchical and complex designs, identification of the appropriate level for tests and full reporting of outcomes
☒	☐	Estimates of effect sizes (e.g. Cohen's *d*, Pearson's *r*), indicating how they were calculated

Our web collection on statistics for biologists contains articles on many of the points above.

Software and code

Policy information about availability of computer code

Data collection	Olympus Fluoview 1000 software was used to acquire confocal microscopy images. EVOS FL Auto 2 software was used to acquire epifluorescence and brightfield images. CellRanger software (version 6.0.1, 10x Genomics) was used to generate scRNA-seq data files. Fluorescence-activated cell sorting was performed using a BD FACSAria Fusion instrument and BD FACSDiva 9.0.1 software. IbidiTreat µ-Slide I Luer0.8 and an Ibidi flow chamber were used tor HAEC experiments.
Data analysis	ImageJ software (version 2.16) was used to analyze images. GraphPad Prism 10 software was used to generate graphs and analyze data. For scRNA-seq analysis, the Seurat package (version 5.0.3) in R (version 1.3.1) was used for all standard processing steps, including data normalization, dimensionality reduction and cell type clustering. We did not use any custom code in this study. We instead provide access to a Github repository that documents the various open-source scRNA-seq packages associated with our analyses at: https://github.com/aryan-kaveh/scRNA-seq-flow-mediated-EC-remodeling-ldlr.

For manuscripts utilizing custom algorithms or software that are central to the research but not yet described in published literature, software must be made available to editors and reviewers. We strongly encourage code deposition in a community repository (e.g. GitHub). See the Nature Portfolio guidelines for submitting code & software for further information.

Data

Policy information about availability of data

All manuscripts must include a data availability statement. This statement should provide the following information, where applicable:

- Accession codes, unique identifiers, or web links for publicly available datasets
- A description of any restrictions on data availability
- For clinical datasets or third party data, please ensure that the statement adheres to our policy

ScRNA-seq data from zebrafish and HAEC models have been submitted to the Gene Expression Omnibus repository and made publicly available under the accession number GSE275308 [https://www.ncbi.nlm.nih.gov/geo/query/acc.cgi?acc=GSE275308]. Additionally, HAEC data used for integration that were sequenced with SeqWell S3 are publicly available at GSE212388 [https://www.ncbi.nlm.nih.gov/geo/query/acc.cgi?acc=GSE212388]. All computational analyses were performed using open-source software. Other data from this study are available within the article, its supplementary figures/files and/or separate source data file.

Research involving human participants, their data, or biological material

Policy information about studies with human participants or human data. See also policy information about sex, gender (identity/presentation), and sexual orientation and race, ethnicity and racism.

Reporting on sex and gender	N/A
Reporting on race, ethnicity, or other socially relevant groupings	N/A
Population characteristics	N/A
Recruitment	N/A
Ethics oversight	N/A

Note that full information on the approval of the study protocol must also be provided in the manuscript.

Field-specific reporting

Please select the one below that is the best fit for your research. If you are not sure, read the appropriate sections before making your selection.

[x] Life sciences [ ] Behavioural & social sciences [ ] Ecological, evolutionary & environmental sciences

For a reference copy of the document with all sections, see nature.com/documents/nr-reporting-summary-flat.pdf

Life sciences study design

All studies must disclose on these points even when the disclosure is negative.

Sample size	Sample sizes were not statistically predetermined. Instead, they were selected based on prior experience with these experimental systems, consistency with sample sizes reported in the literature, and established field standards. The chosen sample sizes were sufficient to ensure reproducibility of observed effects across independent biological replicates and to enable appropriate statistical analyses.
Data exclusions	No data were excluded from the study.
Replication	Experimental results were independently replicated in multiple experiments, with some reproductions performed by different researchers. Independent experiments were performed on at least three different clutches of zebrafish on different days. The number of zebrafish used and independent experiments performed for each figure is indicated in the corresponding legend.
Randomization	Animals were randomly allocated to treatment groups prior to the intervention. No statistical methods were used to assign animals randomly. For some experiments, group assignment was determined based on animal genotype.
Blinding	During initial phenotypic characterization, heterozygote animals were incrossed and the progeny genotyped post experiment which allowed for inherent blinding across all genotypes. For subsequent experiments, investigators were not blinded to group allocation. All groups in the same experiment were subject to the same standards during data collection and analysis.

Reporting for specific materials, systems and methods

We require information from authors about some types of materials, experimental systems and methods used in many studies. Here, indicate whether each material, system or method listed is relevant to your study. If you are not sure if a list item applies to your research, read the appropriate section before selecting a response.

Materials & experimental systems

n/a	Involved in the study
	[x] Antibodies
	[x] Eukaryotic cell lines
[x]	Palaeontology and archaeology
	[x] Animals and other organisms
[x]	Clinical data
[x]	Dual use research of concern
[x]	Plants

Methods

n/a	Involved in the study
[x]	ChIP-seq
	[x] Flow cytometry
[x]	MRI-based neuroimaging

Antibodies

Antibodies used

Primary antibodies were diluted 1:100 in blocking buffer and incubated overnight at 4 °C. The following primary antibodies were used: anti-apolipoprotein B (rabbit polyclonal, Abcam, ab20737), anti-PCNA (mouse monoclonal, Cell Signaling Technology, #2586S; clone PC10), anti-acetylated tubulin (mouse monoclonal, Sigma-Aldrich, #T7451; clone 6-11B-1), and anti-Hsp70l (rabbit polyclonal, Creative Diagnostics, #CABT-B661).
Secondary antibodies were diluted 1:200 and incubated for 1.5 h at room temperature. The following secondary antibodies were used: goat anti-mouse IgG (highly cross-adsorbed, Alexa Fluor 546, Thermo Fisher Scientific, #A-11030), goat anti-rabbit IgG (highly cross-adsorbed, Alexa Fluor 568, Thermo Fisher Scientific, #A-11011), and goat anti-rabbit IgG (highly cross-adsorbed, Alexa Fluor Plus 647, Thermo Fisher Scientific, #A-32733).

Validation

All primary antibodies used in this study were validated by the manufacturers for the reported applications. Antibody specificity was obtained from previously published literature and expected staining patterns.
The anti-apolipoprotein B antibody (Abcam, ab20737) has been validated for detection of ApoB in vertebrate systems and produced staining consistent with lipid-associated structures. The anti-PCNA antibody (Cell Signaling Technology, #2586S) is a widely used proliferation marker, with established nuclear localization in proliferating cells; staining in this study was restricted to nuclei of actively cycling cells, consistent with known PCNA expression patterns. The anti-acetylated tubulin antibody (Sigma-Aldrich, #T7451; clone 6-11B-1) is a well-characterized reagent for stable microtubules and cilia, and produced signal localized to ciliary and microtubule structures consistent with established profiles. The anti-Hsp70l antibody (Creative Diagnostics, #CABT-B661) showed inducible expression patterns consistent with stress-responsive Hsp70 family proteins, in agreement with expected biology. Across all antibodies, staining patterns were reproducible across independent experiments and exhibited low background signal.

Eukaryotic cell lines

Policy information about cell lines and Sex and Gender in Research

Cell line source(s)

Primary human aortic endothelial cells (HAECs, #C-12271) were acquired from Promocell (Heidelberg, Germany).

Authentication

HAECs were authenticated by the manufacturer (Promocell, Heidelberg, Germany). This included testing for for cell morphology, adherence rate, and cell viability. Flow cytometric analyses for cell-type specific markers, CD31 and Dil-Ac-LDL uptake were also carried out. Growth performance was tested through multiple passages up to 15 population doublings under culture conditions without antibiotics or antimycotics.

Mycoplasma contamination

All cells were frequently tested for Mycoplasma contamination, and all cultures tested negative throughout the study.

Commonly misidentified lines
(See ICLAC register)

N/A

Animals and other research organisms

Policy information about studies involving animals; ARRIVE guidelines recommended for reporting animal research, and Sex and Gender in Research

Laboratory animals

Zebrafish (Danio rerio) were used as an animal model. The following transgenic and mutant lines were used in this study: Tg(flk:eGFP)s843, Tg(gata1a:dsRed)sd2, Tg(mpx:GFP)i114, Tg(mfap4:tdTomato)xt12, Tg(cd41:eGFP)la2 and ldlr sd52/sd52. All lines were maintained in-house at Brigham and Women's Hospital. The ldlr sd52 allele was originally generated and described previously (PMID: 29187523). Transgenic lines were obtained from established laboratory stocks as described in the Methods. For imaging experiments, animals were maintained on casper or nacre pigmentation mutant backgrounds. Mutant and transgenic zebrafish were outcrossed to wild type (AB strain) zebrafish after two consecutive incrossings, with new generations derived of embryos from several group crosses. Embryos and larvae were used for all experiments. Developmental stages are reported in the manuscript as days post-fertilisation (dpf) (e.g. 1–5 dpf as specified per experiment).

Wild animals

N/A

Reporting on sex

Since the sex of zebrafish is not specified at embryonic or larval stages, sex discrimination was not involved in our study.

Field-collected samples

N/A

Ethics oversight	All animal experiments were conducted in accordance with protocols approved by the Institutional Animal Care and Use Committee (IACUC) to Brigham and Women's Hospital. All protocols and procedures followed the guidelines and recommendations outlined by by the Guide for the Care and Use of of Laboratory Animals.

Note that full information on the approval of the study protocol must also be provided in the manuscript.

Plants

Seed stocks	N/A
Novel plant genotypes	N/A
Authentication	N/A

Flow Cytometry

Plots

Confirm that:

- [x] The axis labels state the marker and fluorochrome used (e.g. CD4-FITC).
- [x] The axis scales are clearly visible. Include numbers along axes only for bottom left plot of group (a 'group' is an analysis of identical markers).
- [x] All plots are contour plots with outliers or pseudocolor plots.
- [x] A numerical value for number of cells or percentage (with statistics) is provided.

Methodology

Sample preparation	Approximately 200 zebrafish Tg(flk:eGFP) embryos per group were enzymatically dissociated using preheated 0.25% Trypsin-EDTA (Gibco) at 28°C for 20 minutes, with mechanical homogenization performed every 5 minutes to ensure complete dissociation. Following disassociation, embryos were washed with PBS supplemented with 1% Fetal Bovine Serum (FBS). Cell pellets were collected by centrifugation and resuspended in PBS containing 1% FBS in preparation for fluorescence-activated cell sorting.
Instrument	BD FACSAria Fusion Cell Sorter.
Software	BD FACSDiva Software was used to collect flow data. FlowJo was used to analyze the data.
Cell population abundance	Following sorting, approximately 15% of single cells were identified as GFP+ (vascular endothelial) per experimental group and a 1:1 ratio of GFP+ and GFP- cells were processed for downstream analysis.
Gating strategy	A forward and side scatter (FSC/SSC) size selection gate was constructed and validated through back-gating of cells with high GFP expression. Vascular endothelial (GFP+) and non-endothelial (GFP-) single-cell fractions were equally sorted according to the presence of GFP signal. A more detailed explanation is given in Supplementary Figure 7.

- [x] Tick this box to confirm that a figure exemplifying the gating strategy is provided in the Supplementary Information.
