# Supplementary material for: Flow-mediated endothelial remodeling and inflammation drive developmental vascular susceptibility in ldlr loss of function
Source: Nat Commun. 2026 May 21;17:6926. doi: 10.1038/s41467-026-72756-3 (PMC13388945; doi:10.1038/s41467-026-72756-3)
Supplement: Supplementary file 2 — Peer Review file [file 41467_2026_72756_MOESM2_ESM.pdf]

nature portfolio

Peer Review File

Flow-mediated endothelial remodeling and inflammation drive developmental vascular susceptibility in *ldlr* loss of function

Corresponding Author: Dr Aryan Kaveh

This file contains all reviewer reports in order by version, followed by all author rebuttals in order by version.

Version 0:

Reviewer comments:

Reviewer #1

(Remarks to the Author)
Kaveh et al used ldlr-/- zebrafish to study the early stages of atherosclerosis before the development of hypercholesterolemia. Ldlr-/- fish with labeled endothelial cells showed disrupted angiogenesis in the caudal venous plexus (CVP) of zebrafish. Using single RNA-seq analysis, they identify a sub-population of trunk endothelial cells with elevated stress-responsive genes, in particular hsp70. Elevation of hsp70 was blocked by morpholinos or drugs that inhibited cardiac contraction and thus blood flow, which they interpret as due to a requirement for fluid shear stress. Pharmacological activation of Hsp70 recapitulates the phenotypes observed in ldlr-/- fish while hsp70 inhibition suppresses aberrant vascular remodeling. The manuscript thus identifies some interesting but subtle phenotypes in this early vascular development in the zebrafish hypercholesterolemia model.

Despite a number of interesting observations, the study premise and conclusions have significant weaknesses. One prominent difficulty is based on the claim, made in the abstract, repeated in the introduction and again in the discussion that the observed defects are independent of circulating LDL. No measurements of LDL are made in the manuscript, instead they cite a paper (ref 18) examining ldlr morpholino treated embryos where LDL was elevated only at day 5. But this is a different model, morpholinos likely act more slowly than total knockout. In fact, Kaveh et al show increased tissue staining for apoB100 at day 2 when developmental defects are observed. The conclusion that effects are independent of LDL is likely wrong. The insertion of a mild caveat in the Discussion does not ameliorate this major problem.

Specific criticisms.

1. The experiments where the heart is slowed or stopped are interpreted in terms of fluid shear stress. However, the effects could equally be due to decreased transport such that LDL particles do not reach the tissues.

2. Previous studies, not cited, have reported that shear stress activates ER stress responses /UPR in ECs (see PMID 23619421 for a review, PMID 18556570 and 21289099 for primary papers, among others). This diminishes novelty. They also raise questions about the type of shear stress pattern that triggers ER stress.

3. There is a potentially interesting point that the current manuscript touches on but is not developed in a satisfactory way, that the activation of ER stress by disturbed flow may be amplified by high LDL. More careful and convincing exploration of this idea could make for a significant paper.

4. The authors need to correlate their observations and underline significant deviations from the athero-studies in higher organisms. The Hsp70 gene family has a positive role in mitigating cell injury when present intracellularly, however, serum Hsp70 is thought to exacerbate ASCVD. How does this study correlate with the above observations?

5. Many of the images of vascular morphology in vivo are of low magnification and poor resolution, and do not allow readers to assess the phenotypes that are scored. The authors need to provide better images.

Reviewer #2

(Remarks to the Author)
I co-reviewed this manuscript with one of the reviewers who provided the listed reports. This is part of the Nature Communications initiative to facilitate training in peer review and to provide appropriate recognition for Early Career Researchers who co-review manuscripts.

Reviewer #3

(Remarks to the Author)
Dear Editor,

The current study by Kaveh et al. aims to investigate the regulation of flow-dependent endothelial cell (EC) Hsp70 in EC function and inflammation during the development of ldlr-/- zebrafish. The authors show that Ldlr knockout impairs angiogenesis and ciliogenesis and increases apoptosis, particularly in the caudal venous plexus (CVP) at 2-3 days dpf. The group identified hsp70 as a crucial gene that regulates the stressed EC in response to different flows using tnnt2a blocker (reduced flow) and BDM (static flow) in the ldlr-/- zebrafish development. They also demonstrate the Hsp70 in response to physiological and pathophysiological shear stress using HAECs. By using Hsp70 inhibitor (VER) and activator (17-DMAG), the authors demonstrate the role of hsp70 in vascular morphology during zebrafish development. The authors also proposed that hsp70 modulates EC regeneration and neutrophilic inflammation. Although the manuscript is well-written and intriguing, there are several major and minor concerns that would need to be addressed. These concerns are following:

Major:
1. The role of Ldlr and Ldlr knockout animal models is well-established in larger models such as mice, including studies on EC dysfunction associated with atherosclerosis using both human and mouse models. Therefore, the authors should clarify the necessity of using a zebrafish development model. They should discuss the similarities and differences compared to larger animal models. Additionally, the authors emphasize Ldlr in atherosclerosis, yet clinically, atherosclerosis predominantly occurs in adults or with aging. The authors need to address the relevance of studying atherosclerosis in a developmental context.
2. The authors use DASPEI and BODIPY staining to examine hair cell development as a mechanosensory, but it is unclear how these findings relate to mechanosensory phenotypes. Although cilia is a mechanosensor in ECs, there are many other canonical mechanosensors in ECs. Please provide additional explanation or data to clarify how the observed changes in hair cell development contribute to mechanosensor in CVP of zebrafish.
3. In Figure 3A and S3, the proportion of stressed endothelial cells (EC) is very limited. In the HAEC scRNAseq data, the stressed EC cluster (HSP70+, HSS 5) also exhibited a limited number on the UMAP (Figure S9). Given that physiological shear stress typically promotes athero-protective and anti-inflammatory phenotypes in ECs. It is unclear why only a small subset exhibits opposite effects in response to HSS in HAECs or CVP in zebrafish. The manipulation of HSP70, which is expressed in only a small portion of the EC population, leads to vascular regeneration and inflammation. The manuscript should clarify how targeting this minor fraction of trunk ECs influences such significant phenotypic changes.
4. It is difficult to ascertain from the current data how these stressed ECs play a crucial role in ldlr-/- zebrafish development.
5. In the discussion, the authors mention that the CVP is exposed to disturbed flow or lower shear stress. Therefore, reduced flow in CVP is not necessary. Additionally, the comparison between physiological flow in HAECs and CVP EC is not clearly matched. This point should be addressed.
6. Please review each dataset for completeness; some parts are missing quantifications or representative images. Providing these details will ensure the data supports the results more robustly.
7. It vascular phenotype of ldlr-/- zebrafish between 2-3 dpf. But the vascular phenotype including hsp70l expression has recovered after 5 dpf. Please discuss this time-dependent manner effects.
8. For the in vitro experiments, have you considered using HSP70 siRNA or an inhibitor to inhibit the effects of HSP70?
9. The ldlr-/- EC phenotypes have recovered after 4-5 dpf. Is this due to the change of hemodynamics during the development? For example, flow in PCV is higher at 5 dpf vs. 2 dpf? It would be great to compare hemodynamics at different time points.
10. Please carefully review all figures to ensure completeness, as some quantifications, representative images, and comparisons are missing. Additionally, the data presentation in the main manuscript should be reorganized to improve readability. Currently, the layout is overly dense and confusing.
11. Please explain and provide more details on scRNAseq data analysis such as cell markers, statistics, etc. Specifically, describe how you annotated the cell types in each dataset, especially how to segregate 3 different EC populations.
12. The flow study in zebrafish is well-designed. For the scRNA seq, the authors used tnnt2a MO to minimize off-target effects, and used Control MO. In the following violin plot of hsp70l expression (Figure 3B), it shows that in the "normal ECs", hsp70l is higher in ldlr KO embryos under reduced flow as compared to normal flow. The authors should explain the indication of this observation, as the other data suggests that reduced flow leads to reduced hsp70l activation. Providing a UMAP expression plot of hsp70l and annotations of the two EC types and experimental groups would help clarify this.
13. The authors presented in situ hybridization (ISH) of hsp70l mRNA on BDM vs treated ldlr KO embryos. Would it be possible to perform hsp70l ISH on the tnnt2a M-treated group? the reason is that BDM is known to have many off-target effects, and it is more representative of "static flow", not reduced flow (Figure S5B). Staining on the tnnt2a MO groups would be a better validation of their scRNA seq.
14. hsp70l showed elevation in ldlr KO at 2 dpf but then returned to normal at 3 dpf (Figure 2D). This is an intriguing phenomenon, as 2-3 dpf is a major window for cardiac development, and a significant increase of cardiac output (flow). I would recommend the authors also look into hsp70l expression in tnnt2a-treated WT and ldlr KO embryos at 3-4 dpf. This

may reveal more about the relationship between hsp70l and flow.

Minor:
1. For Figure 1C, please include images from ldlr+/+ for the xz and yz planes for a more comprehensive comparison. Also, the representative images for cilia are not clear.
2. In Figure 1D, please provide quantification data for the BODIPY staining to support the visual observations made from the staining results.
3. Please provide representative images for 4 and 5 dpf. Also, indicate the dorsal aorta (DA), intersegmental vessels (ISV), and posterior cardinal vein (PCV) in the representative images if these structures are visible. If these structures are not currently shown, please include images that display them.
4. Additionally, it is unclear whether the stressed ECs are derived from trunk ECs.
5. Additionally, please interpret the Figure 3F well.
6. For Figure 2E, relative mRNA expression is generally set the value as 1 for the control. Also, it would be better to have the dot plot and bar graph.
7. In Figure 4, would it be better to replace the bottom of panel A with Figure S9?
8. Using an activator or inhibitor of Hsp70 in ldlr+/+ and ldlr-/- zebrafish is a key experiment in this study. Thus, it has to be carefully dissected out the results in Figure 5. Additionally, some data is not consistent with results in the Figure 5. For example, Hsp70 inhibitor (VER) or CRISPR increased apoptosis in both ldlr+/+ and ldlr-/-, whereas VER decreased the CVP pillar and increased CVP cilia # in Figure 5D. Also, there are no effects of VER on CVP pillar and cilia in ldlr+/+ fishes in Figure 5D. Please address this discrepancy.

Reviewer #4

(Remarks to the Author)
Noteworthy results
The authors use a new zebrafish ldlr knockout model of familial hypercholesterolemia to study endothelial cells at early stages before cholesterol becomes elevated. They show that there are stressed endothelial cells in early development and identified a gene program leading to hsp70 expression. Hsp70 expression was only induced with physiological flow, a similar result to human endothelial coronary artery cells where hsp expression was flow dependent. Ciliogenesis and intussusceptive angiogenesis are affected in the zebrafish model of ldlr loss, and apoptosis is induced in endothelial cells. If hsp70 is inhibited or activated in ldlr fish, there is a corresponding rescue or worsening of apoptosis, angiogenesis and ciliogenesis suggesting hsp70 marks cells that behave abnormally. Downstream angiogenesis defects and alteration in immune cells as seen. Overall, the authors suggest a novel hypothesis, that early abnormalities that give rise to endothelial cell dysfunction and promote inflammation prior to the emergence of hypercholesterolemia.

Significance and novelty
Overall, the data are novel. No other model (fish or mouse) has shown similar early endothelial defects prior to cholesterol misregulation. The connection to hsp70 upregulation is also novel. Findings on immune dysregulation (neutrophils), and ciliogenesis defects are also unique. Overall, there is a substantial novelty in the data and approach of the manuscript.

There are several points that must be addressed:

1. The reviewer is left wondering why scattered cells upregulate hsp70, particularly in light of the later findings with blood flow. There is a missed opportunity to understand where the hsp70 expressing cells arise in vivo. Blood flow in the CVP is heterogeneous, faster in the larger vessels, slower in the smaller vessels. Flow will also change at bifurcations. The localization of the hsp70 expressing cells with respect to the architecture would be very informative. Are the hsp70 expressing cells only in the smallest vessels or large ones? Are they in remodelling vessels or in vessels that will be maintained? How does their location relate to blood flow? Do they have altered morphology? Understanding this relationship would strengthen the claim that blood flow interrelates with hsp70 expression downstream of a stress program.

Is hsp70 expressed downstream of mechanosensory pathways?
Is ciliogenesis disrupted in all endothelial cells, or only the ‘stressed’ cells? Cell level resolution would be informative as it could indicate that the most severely stressed cells that are scattered throughout vessels are also the same cells that show other phenotypes. If all cilia are affected, why does rescue with VER, which would affect cells expressing hsp70 have an effect?
In Figure 5D there does not appear to be significance between DMSO for LDLR positive and mutant cells. Is this an oversight(i.e. not placing significance bars on the graph) as in earlier figures they are significantly different?

2. Images of critical data to support claims (intussusceptive pillars) are too low resolution to view the underlying data. Better resolution images are imperative for all data supporting a claim that there is altered intussusceptive angiogenesis. Because these are not visible from the images provided, it makes the reviewer wonder whether the authors correctly counted pillars. An excellent example of what the pillars should look like can be found in figure 2 of this paper:
https://doi.org/10.1038/s41598-018-27791-6
Insets must be shown to support every claim of decreased intussusceptive angiogenesis. Methods of measurement (where, when, how) should be briefly outlined in the results, and marked on at least one of the images. The pillars are within single vessels and are distinguished from loops. How was the largest pillar identified.

Cilia are also similarly shown at too low resolution to support the data.
3. The authors used huAECs under different flow conditions to show that Ldlr and Hsp70 orthologs (HSPA1A/B) and other stress genes are downregulated with reduced flow, matching the fish data, although there is heterogeneity in the samples. There is very little data on the HuAECs from the commercial supplier, but were all HuAECs from a single donor? How do the authors know that the heterogeneity in the scRNAseq is not just due to genetic background effects? It should be clarified in the methods whether different batches/donors were used.

4. Similar to the above point about flow and heterogeneity in fish, flow devices may have slightly uneven flow. In situ, are the cells at the edge (or middle of the device) more likely to express hsp70 as a response to differential flow?

5. The HuAECs are wildtype. The relevance to ldlr knockout heterogeneity is questionable since in ldlr mutant fish cells under flow, hsp70 is upregulated, but in the human wildtype cells, HSP1 is uniformly expressed in physiological flow. Why are the human cells behaving differently?
a. What happens to the gene expression when LDLR is reduced in the human cells (siRNA)?
b. An alternative to the human data would be to analyze the hsp70 expression in ldlr mutant embryonic mice. This is not necessary for publication in our opinion, but would dramatically increase the impact of the findings.

6. Multiple sets of data show that phenotypes appear and then normalize in the zebrafish over time. For instance, there is a stark increase at 2 dpf of apoptosis, apolipoprotein B particles, PCNA cilia length and other phenotypes, but these resolve by 3 dpf. This is puzzling and not sufficiently rationalized. The authors suggest there is a 'normalization of CVP EC turnover'. Are the stressed cells dead at this point? Or are the authors certain that this is not genetic compensation by other pathways that might compensate for ldlr loss?

7. There is no difference in the head vessel apoptosis, yet increased apoptosis line in the trunk. Is there a difference between head and trunk lipoprotein metabolism? Along the same lines, why are the DLAV and CVP more affected, as opposed to other vessels? Could this be a flow dependent effect?

8. The idea that there is a connection between hair cells and endothelial cilia is not supported by any analysis in any other ciliated cell type in the zebrafish. There likely is no connection other than the cells are ciliated. If the authors wish to keep this statement, they should show that the phenotype is unique to these two cell types and not other ciliated cells.

9. The supplied word file for the supplementary data is at unacceptable resolution. The reviewer assumes this was a mistaken upload. This must be corrected.

10. Figure 1, panel c. For ciliated pillar, show boxes to show insets (XZ and YZ). Where is the pillar?

11. Fig 1 Graphs have different axes, and axes don't start at 0. It is very hard to compare different timepoints due to the changing axes. If the 2 and 3 dpf data on the same graph, it would give perspective on how parameters change over time.

12. Fig 5: graph in D, top right is missing the legend below the graph.

13. Have the authors tested whether any of the phenotypes are cholesterol-dependent? Do statins rescue the early phenotypes?

14. The one experiment that may not meet field standards is the CRISPR F0 analysis of hsp70 mutation. This experiment is backed up by chemical inhibitor data, but we believe that the zebrafish field standard is that the data should be backed up by a germline knockout due to possible transient effects due to the CRISPR knockout (i.e. stress effects, which would be relevant to this manuscript topic). Can the authors please address this?

Version 1:

Reviewer comments:

Reviewer #4

(Remarks to the Author)
The authors have done an excellent job in this revision. Their reply to reviewers is precise and detailed (perhaps a little overly long even), and outlines the rationale for changes and for a few experiments that they could not perform. I am satisfied the the queries that I and other reviewers raised have been met. The addition of the model figure in this revision was good.

Open Access This Peer Review File is licensed under a Creative Commons Attribution 4.0 International License, which permits use, sharing, adaptation, distribution and reproduction in any medium or format, as long as you give appropriate credit to the original author(s) and the source, provide a link to the Creative Commons license, and indicate if changes were made.
In cases where reviewers are anonymous, credit should be given to 'Anonymous Referee' and the source.
The images or other third party material in this Peer Review File are included in the article's Creative Commons license, unless indicated otherwise in a credit line to the material. If material is not included in the article's Creative Commons license and your intended use is not permitted by statutory regulation or exceeds the permitted use, you will need to obtain permission directly from the copyright holder.
To view a copy of this license, visit https://creativecommons.org/licenses/by/4.0/

Response to Reviewers

Dear Reviewers,

Thank you very much for giving us the opportunity to submit a revised version of our manuscript, “Flow-mediated endothelial remodeling and inflammation drive developmental vascular susceptibility in *ldlr* loss of function” for further consideration at *Nature Communications*. There were several important and insightful points raised, and we have performed additional experiments, expanded our analyses and discussion points to address the concerns raised. We believe these revisions have significantly strengthened the paper and provided further support for our core conclusions regarding the role of flow in regulating angiogenic remodeling in developmental vascular susceptibility.

Specific highlights of our revision include:

1. **Lipid analysis.** Analysis of hyperlipidemia and LDL-cholesterol levels in *ldlr* knockout embryonic and larval zebrafish. We also include experimental data in the rebuttal on the effect of statin treatments on developmental angiogenesis.
2. **Hemodynamic analysis**. Analysis of red blood cell migration which revealed flow heterogeneity *in vivo* and confirmed the dorsal venous plexus as the primary site of abnormal remodeling angiogenesis and *hsp70*+ endothelial cells.
3. **Mechanistic validation.** Validation of flow-dependent *hsp70*+ endothelial induction using fluorescence *in situ* hybridization, confocal imaging and scRNA-seq analysis.
4. **Expanded contextualization.** Further contextualizing the zebrafish model with other atherosclerosis systems; expanding on cell stress and lipid/metabolomic interactions in the Discussion.
5. **Visual synthesis.** Inclusion of a new summary schematic (**Figure 7**) to illustrate the temporal dynamics of the model, along with higher resolution figure panels, and reorganized figures for clarity.

We also provide a more detailed comparative perspective between zebrafish and human endothelial cell data, new spatial analyses and additional scRNA-seq supplementary information and metadata files.

The manuscript edits are visible in tracked changes, and our point-by-point responses are provided in blue text below. Thank you again for your continued consideration.

Best wishes,

Aryan Kaveh

Reviewer #1 (Remarks to the Author):

Kaveh et al used ldlr-/- zebrafish to study the early stages of atherosclerosis before the development of hypercholesterolemia. Ldlr-/- fish with labeled endothelial cells showed disrupted angiogenesis in the caudal venous plexus (CVP) of zebrafish. Using single RNA-seq analysis, they identify a sub-population of trunk endothelial cells with elevated stress-responsive genes, in particular hsp70. Elevation of hsp70 was blocked by morpholinos or drugs that inhibited cardiac contraction and thus blood flow, which they interpret as due to a requirement for fluid shear stress. Pharmacological activation of Hsp70 recapitulates the phenotypes observed in ldlr-/- fish while hsp70 inhibition suppresses aberrant vascular remodeling. The manuscript thus identifies some interesting but subtle phenotypes in this early vascular development in the zebrafish hypercholesterolemia model.

Despite a number of interesting observations, the study premise and conclusions have significant weaknesses. One prominent difficulty is based on the claim, made in the abstract, repeated in the introduction and again in the discussion that the observed defects are independent of circulating LDL. No measurements of LDL are made in the manuscript, instead they cite a paper (ref 18) examining ldlr morpholino treated embryos where LDL was elevated only at day 5. But this is a different model, morpholinos likely act more slowly than total knockout. In fact, Kaveh et al show increased tissue staining for apoB100 at day 2 when developmental defects are observed. The conclusion that effects are independent of LDL is likely wrong. The insertion of a mild caveat in the Discussion does not ameliorate this major problem.

We thank the reviewer for this careful and thoughtful critique, which highlighted an important issue regarding the interpretation of LDL dependence in our study. We agree that the original manuscript did not sufficiently substantiate the conclusion that the early vascular phenotypes are independent of LDL, and we have therefore substantially revised both the experimental data and the framing of our conclusions.

First, we would like to clarify that our study uses a germline *ldlr* loss of function mutant, not a morpholino-based knockdown. The *ldlra*$^{sd52/sd52}$ allele, originally described by Liu et al., 2018 (PMID: 29187523, our original ref. 18), was generated by CRISPR/Cas9-mediated genome editing and contains a 10 bp deletion resulting in a frameshift and premature stop codon. Homozygous *ldlr*-/- zebrafish therefore represent a stable genetic knockout rather than a transient or delayed knockdown model. Liu et al. demonstrated that *ldlr-/-* zebrafish larvae exhibit elevated cholesterol and triglyceride levels from 5 dpf (PMID: 29187523, Figures 3C-D), which motivated our focus on primary vascular phenotypes arising earlier, between 1-5dpf, prior to overt hypercholesterolemia. However, as the reviewer correctly points out, our data also raise the possibility that LDL-related processes may contribute to the observed developmental defects.

To directly address this concern, we have now performed additional experiments, including direct measurements of LDL levels, and have revised the manuscript accordingly. These new data, together, with revised text in the Abstract, Results and Discussion, more carefully delineate the extent to which the early endothelial phenotypes precede or may partially overlap with changes in LDL. Detailed responses to each specific point raised by the reviewer are provided below.

Specific criticisms.

1. The experiments where the heart is slowed or stopped are interpreted in terms of fluid shear stress. However, the effects could equally be due to decreased transport such that LDL particles do not reach the tissues.

We thank the reviewer for raising this important alternative interpretation. We agree that reduced cardiac output, could, in principle, affect vascular phenotypes by altering lipoprotein transport rather than fluid shear stress per se. To address this possibility, we have performed additional experiments and revised the manuscript accordingly.

First, we directly measured whole-body LDL/VLDL levels in *ldlr*+/+ and *ldlr*-/- zebrafish at 2 dpf and 5 dpf (**new Figure S1B**). At 2 dpf, LDL/VLDL levels were low and indistinguishable between genotypes. In contrast, at 5 dpf there was a developmental increase in LDL/VLDL in both groups, with a further significant elevation in *ldlr*-/- larvae, consistent with prior reports (Liu et al., 2018).

We attempted to corroborate these findings using circulatory blood measurements; however, the extremely small blood volumes obtainable at these developmental stages (<1nL at 2 dpf and <2nL at 5 dpf) precluded reliable detection above background. We therefore additionally assessed lipid accumulation using Oil Red O staining,

which indicated background-level staining between genotypes at 2 dpf, but increased vascular staining in *ldlr*-/- larvae at 5 dpf (**new Figure S1A**).

Together, these data indicate that overt elevations in circulating LDL/VLDL are unlikely to account for the early (2 dpf) vascular phenotypes. That said, as the reviewer notes, we do observe a transient increase in apoB immunostaining in the caudal venous plexus of *ldlr*-/- embryos at 2 dpf. Based on recent work demonstrating reduced turnover and prolonged half-life of apoB-containing lipoproteins in the same *ldlra*$^{sd52/sd52}$ model (Moll et al., 2025, PMID: 40836916), we now interpret this finding as reflecting altered local vascular handling of apoB-containing particles rather than systemic hyperlipidemia and hypercholesteremia which manifest later in development (Thierer et al., 2019, PMID: 31366908, Moll et al., 2025, PMID: 40836916, Liu et al.,. 2018, **new figure S1A-B**)

In light of these considerations, we have removed all statements stating that the observed phenotypes are independent of LDL or hypercholesterolemia and have revised the Discussion to emphasize how flow-dependent endothelial remodeling may intersect with altered vascular lipoprotein trafficking in *ldlr*-/- zebrafish (also related to our response to points 2, 3 and 4 from reviewer 1 in which we further expand).

2. Previous studies, not cited, have reported that shear stress activates ER stress responses /UPR in ECs (see PMID 23619421 for a review, PMID 18556570 and 21289099 for primary papers, among others). This diminishes novelty. They also raise questions about the type of shear stress pattern that triggers ER stress.

We thank the reviewer for drawing attention to prior work demonstrating shear stress-dependent activation of ER stress and UPR pathways in in endothelial cells. We agree that this literature is important to acknowledge and have now updated our Discussion accordingly, clarifying how our findings relate to and extend beyond these studies.

The studies referenced primarily focus on ER-resident chaperones, particularly HSPA5/GRP78, and UPR signaling in adult mammalian endothelial cells exposed to defined low/disturbed shear stress, often in the context of atherosusceptible arterial regions (Feaver et al., 2008, PMID: 18556570; Matsushita et al., 2011, PMID: 21289099). By contrast, our study identifies a flow- and cell stress-responsive endothelial subpopulation characterized by induction of the molecular chaperone, HSP70, during early vascular development in *ldlr*-/- zebrafish.

Importantly, our scRNA-seq analysis of human aortic endothelial cells exposed to graded flow conditions revealed that while classical ER stress markers (including HSPA5, ATF4, DDIT3 and XBP1) show heterogenous expression across shear stress states, HSP70 and DNAJB1 in particular display flow-associated expression coupling not reported in prior studies (**Figure 4C-D**). This expression pattern parallels our zebrafish endothelial scRNA-seq data (**Figure 3C**), supporting cross-species conservation of this response.

In zebrafish, we further show that ER stress signaling precedes and contributes to *hsp70*+ endothelial cell induction. Specifically, *hspa5* and *ddit3* (CHOP) are upregulated at 1 dpf, coincident with increased endothelial apoptosis. Additionally, pharmacological inhibition of p38 Mapk, previously shown to inhibit HSPA5 upregulation (Feaver et al., 2008), abolishes the emergence of *hsp70*+ endothelial cells and rescues vascular patterning defects. These data indicate that ER stress is positioned upstream of cytoplasmic chaperone activation.

Regarding the type of blood flow patterns involved *in vivo*, we have now performed additional hemodynamic analyses in relation to the vascular abnormalities in *ldlr*-/- zebrafish. Live imaging and red blood cell (RBC) tracking revealed region-specific disturbed flow within the dorsal caudal venous plexus (dCVP) of *ldlr*-/- embryos, characterized by reduced RBC velocity, precisely where *hsp70*+ endothelial cells accumulate and where there are additional vascular loops and apoptotic endothelial cells (**new Figure 1D-E**). In contrast, the adjacent ventral caudal venous plexus (vCVP), which displays higher RBC velocities and more uniform flow, shows relatively fewer *hsp70*+ and apoptotic endothelial cells. We also provide new staining, imaging and scRNA-seq data supporting the physiological flow-dependent upregulation of *hsp70*+ ECs in *ldlr*-/- zebrafish and how it correlates to abnormal CVP remodeling and altered RBC movement (**Figure 2D**, **Figure S9**, **Figure S11**, **Figure S14**).

Together, our data support a model in which early ER stress signaling converges with disturbed developmental blood flow to induce *hsp70*, which in turn disrupts angiogenic remodeling. We have revised the Results and Discussion to articulate this distinction from prior UPR-focused studies and to highlight the mechanistic and conceptual novelty of our findings, and we now also include a summary schematic to convey this model (**Figure 7**).

3. There is a potentially interesting point that the current manuscript touches on but is not developed in a satisfactory way, that the activation of ER stress by disturbed flow may be amplified by high LDL. More careful and convincing exploration of this idea could make for a significant paper.

We thank the reviewer for highlighting this important conceptual possibility. We agree that amplification of disturbed flow-induced cell stress by elevated LDL would represent a compelling mechanism that could enhance the impact of the study.

Our data indicate that key components of ER stress signaling and endothelial apoptosis are already elevated at 1 dpf in *ldlr*-/- embryos, preceding both detectable increases in LDL/VLDL and the transient elevation of apoB immunostaining observed at 2 dpf. This temporal sequence suggests that initiation of endothelial cell death and ER stress occurs upstream of overt LDL elevation during early vascular development.

To directly test whether transient increases in apoB/LDL could amplify vascular stress responses, we performed additional pharmacological experiments using two clinically used LDL-lowering statins, atorvastatin and simvastatin (**Figure 1 for reviewer**). Across dose-response analyses, both compounds induced dose-dependent vascular toxicity, including CVP regression, vascular blebbing and intracranial haemorrhage, at nanomolar concentrations in both *ldlr*+/+ and *ldlr*-/- embryos. At higher doses, atorvastatin caused embryonic lethality, consistent with its known potency. Disturbances in CVP patterning and intracranial haemorrhage were detected as low as 5 nM and 10 nM for atorvastatin and simvastatin, respectively. Additionally, statin-induced vascular defects were comparable between genotypes, indicating that these effects were likely not specific to LDL lowering in the *ldlr*-/- background. Together, these findings suggest that statin-mediated toxicity and pleiotropic effects could confound their use as tools to selectively interrogate LDL-dependent modulation of endothelial stress during early zebrafish development.

Based on these results, we conclude that while LDL likely influences later stages of vascular development, our current data do not directly support that LDL amplification is required for the initiation of early endothelial stress and disturbed-flow associated vascular dysfunction in *ldlr*-/- embryos. That said, we have now highlighted in the Discussion how lipid and cell stress interactions are an important area of further investigation.

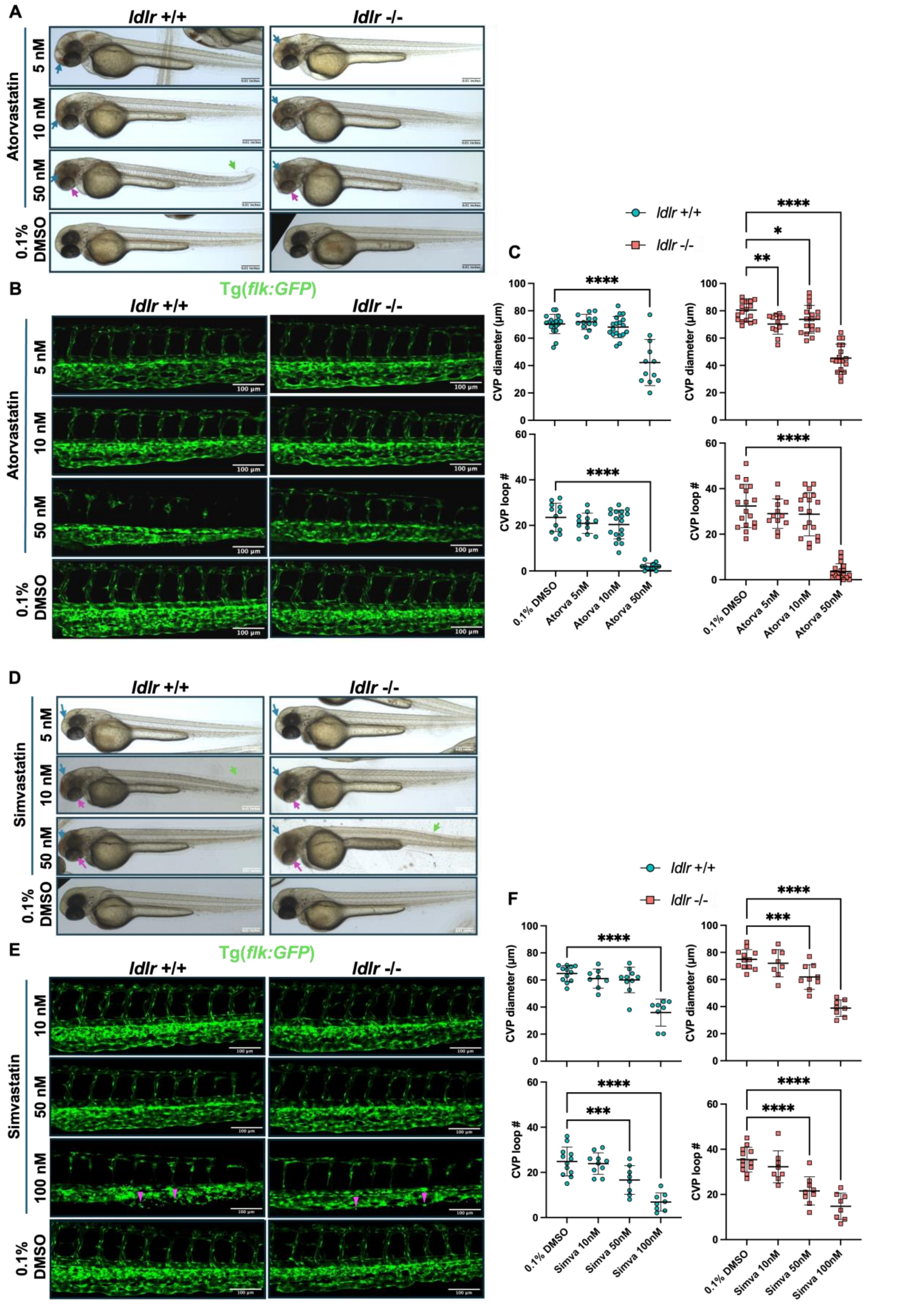

Figure 1 for reviewer: effect of Atorvastatin and Simvastatin on developmental vascular phenotypes in embryonic *ldlr*+/+ and *ldlr*-/- zebrafish. (A) Brightfield microscopy of *ldlr*+/+ and *ldlr*-/- zebrafish treated (1-2 dpf) with 5nM, 10nM, 50nM Atorvastatin or DMSO control at 2 dpf. Blue and magenta arrows indicate dorsal and ventral intracranial haemorrhage sites respectively. Green arrow indicates increased curvature in body axis. **(B)** Confocal microscopy of *ldlr*+/+ and *ldlr*-/- zebrafish Tg(*flk:GFP*) trunk vasculature treated with 5nM,

10nM, 50nM Atorvastatin or DMSO control at 2 dpf. **(C)** Quantification of caudal venous plexus diameter (top) and loop number (bottom) in *ldlr*+/+ and *ldlr*-/- zebrafish treated with 5nM, 10nM, 50nM Atorvastatin or DMSO control at 2 dpf. Data are mean ± s.d. One-way ANOVA and Holm-Sidak's multiple comparison used. **(D)** Brightfield microscopy of *ldlr*+/+ and *ldlr*-/- zebrafish treated (1-2 dpf) with 10nM, 50nM, 100nM Simvastatin or DMSO control at 2 dpf. Blue and magenta arrows indicate dorsal and ventral intracranial haemorrhage sites respectively. Green arrow indicates increased curvature in body axis. **(E)** Confocal microscopy of *ldlr*+/+ and *ldlr*-/- zebrafish Tg(*flk:GFP*) trunk vasculature treated with 10nM, 50nM, 100nM Simvastatin or DMSO control at 2 dpf. Magenta arrowheads indicate caudal venous plexus endothelial blebbing suggestive of cell death in embryos treated with 100nM simvastatin. **(F)** Quantification of caudal venous plexus diameter (top) and loop number (bottom) in *ldlr*+/+ and *ldlr*-/- zebrafish treated with 10nM, 50nM, 100nM Simvastatin or DMSO control at 2 dpf. Data are mean ± s.d. One-way ANOVA and Holm-Sidak's multiple comparison used.

4. The authors need to correlate their observations and underline significant deviations from the athero-studies in higher organisms. The Hsp70 gene family has a positive role in mitigating cell injury when present intracellularly, however, serum Hsp70 is thought to exacerbate ASCVD. How does this study correlate with the above observations?

We thank the reviewer for raising this important conceptual point. We agree that the role of HSP70 in vascular biology is context dependent and that distinguishing our findings from established atherosclerosis studies in higher organisms is essential.

In mammalian models, intracellular HSP70 is generally regarded as cytoprotective, whereas extracellular or circulating HSP70 has been associated with immune activation and vascular inflammation. Our study interrogates *hsp70* induction within endothelial cells rather than serum HSP70-mediated effects. Our data indicate that in the context of early vascular development, *hsp70*+ endothelial cells emerge in regions of disturbed physiological flow, apoptosis and altered angiogenic remodeling. Furthermore, pharmacological activation or inhibition of Hsp70 respectively exacerbates or rescues vascular patterning defects. Thus, in this developmental setting, our working model is that endothelial *hsp70* reflects an attempt to resolve cellular stress, rather than successful cytoprotection (new schematic model**, Figure 7**).

This interpretation does not contradict the established protective roles of intracellular Hsp70 in adult tissues or the pro-inflammatory role of circulating HSP70. Instead, our findings demonstrate a previously unexplored developmental window in which endothelial stress responses, shaped by blood flow and genetic susceptibility, regulate vascular patterning and disease susceptibility. We have revised the Discussion accordingly, comparing our findings with mammalian studies, and to clarify that our work identifies a unique developmental role for endothelial *hsp70.*

5. Many of the images of vascular morphology in vivo are of low magnification and poor resolution, and do not allow readers to assess the phenotypes that are scored. The authors need to provide better images.

We thank the reviewer for this constructive comment. We have now improved the presentation of vascular morphology throughout the main and supplementary figures. Specifically, we have included additional higher resolution confocal insets, highlighting the scored phenotypes. Where possible, we have also updated figure legends to indicate anatomical regions and features used for quantification.

Reviewer #2 (Remarks to the Author):

I co-reviewed this manuscript with one of the reviewers who provided the listed reports (reviewer 4). This is part of the Nature Communications initiative to facilitate training in peer review and to provide appropriate recognition for Early Career Researchers who co-review manuscripts.

We thank the reviewer for their contribution in assessing our manuscript. We have included our point-by-point response to the corresponding reviewer below.

Reviewer #3 (Remarks to the Author):

Dear Editor,

The current study by Kaveh et al. aims to investigate the regulation of flow-dependent endothelial cell (EC) Hsp70 in EC function and inflammation during the development of ldlr-/- zebrafish. The authors show that Ldlr knockout impairs angiogenesis and ciliogenesis and increases apoptosis, particularly in the caudal venous plexus

(CVP) at 2-3 days dpf. The group identified hsp70 as a crucial gene that regulates the stressed EC in response to different flows using tnnt2a blocker (reduced flow) and BDM (static flow) in the ldlr-/- zebrafish development. They also demonstrate the Hsp70 in response to physiological and pathophysiological shear stress using HAECs. By using Hsp70 inhibitor (VER) and activator (17-DMAG), the authors demonstrate the role of hsp70 in vascular morphology during zebrafish development. The authors also proposed that hsp70 modulates EC regeneration and neutrophilic inflammation. Although the manuscript is well-written and intriguing, there are several major and minor concerns that would need to be addressed. These concerns are following:

We thank the reviewer for their thorough evaluation, thoughtful comments and interest in our work. We have carefully considered each concern and have addressed them through additional analyses, new data where appropriate and clarification in the revised manuscript. Detailed point-by-point response are provided below.

Major:

1. The role of Ldlr and Ldlr knockout animal models is well-established in larger models such as mice, including studies on EC dysfunction associated with atherosclerosis using both human and mouse models. Therefore, the authors should clarify the necessity of using a zebrafish development model. They should discuss the similarities and differences compared to larger animal models. Additionally, the authors emphasize Ldlr in atherosclerosis, yet clinically, atherosclerosis predominantly occurs in adults or with aging. The authors need to address the relevance of studying atherosclerosis in a developmental context.

We thank the reviewer for raising these important points regarding model choice and disease relevance. We agree that Ldlr knockout animals and atherosclerosis-associated dysfunction have been extensively studied in adult mouse and human systems, and we have revised the manuscript to more clearly position our zebrafish model as complementary to these established approaches.

The primary rationale for using zebrafish lies in their unique suitability for *in vivo* analysis of endothelial behaviours during early vascular development, including real-time imaging of blood flow, angiogenic remodeling and cell state transitions at single-cell resolution. These features allowed us to interrogate flow-dependent endothelial stress responses in an intact organism, which remains technically challenging in larger vertebrate models, particularly at early developmental stages.

Although atherosclerosis typically manifests in adulthood, there is growing recognition that early-life vascular dysfunction can predispose vessels to later disease susceptibility. Our study therefore focuses on a developmental window in which genetic loss of Ldlr intersects with physiological blood flow to induce a unique endothelial stress program, impaired angiogenic remodeling, and influence inflammatory responses, processes that are mechanistically relevant to atherosclerosis.

We now discuss the similarities and differences between the zebrafish *ldlr*-/- model and adult mammalian models, including lesion complexity, developmental context of the vasculature, and the emphasis on endothelial-intrinsic stress responses. By doing so, we aim to clarify that our findings provide insight into early, flow-sensitive mechanisms that shape vascular vulnerability, complementing existing larger animal studies that focus on later stages of disease.

These points have been incorporated into the Introduction and Discussion to articulate the relevance, strengths and limitations of the developmental zebrafish model in the context of atherosclerosis research.

2. The authors use DASPEI and BODIPY staining to examine hair cell development as a mechanosensory, but it is unclear how these findings relate to mechanosensory phenotypes. Although cilia is a mechanosensor in ECs, there are many other canonical mechanosensors in ECs. Please provide additional explanation or data to clarify how the observed changes in hair cell development contribute to mechanosensor in CVP of zebrafish.

We thank the reviewer for raising this important point regarding mechanosensory interpretation. We agree that endothelial cells possess multiple canonical mechanosensors beyond primary cilia and that neuromast hair cells should not be considered direct proxies for endothelial mechanotransduction.

Our initial inclusion of neuromast hair cell analyses was exploratory and motivated by the observation that ciliary and microtubule-associated genes were altered in pilot experiments of bulk RNA-sequencing in *ldlr*-/- embryos, prompting us to ask whether other mechanosensitive cell types exhibit related developmental sensitivity. Importantly, we do not propose that neuromast hair cells model endothelial flow sensing in the CVP. Rather after

initially characterizing there was a neuromast defect in *ldlr*-/- embryos, we found the Hsp70 modulatory compounds to exert similar detrimental or rescuing effects between the CVP and neuromast systems, raising our interest.

To further clarify this point, we have now examined known hair cell-specific genes in our zebrafish endothelial scRNA-seq dataset and found limited enrichment of canonical hair cell markers in endothelial populations. Conversely, analysis of the most significantly enriched genes in the *hsp70+* endothelial subpopulation (**Figure S8B**) identified some overlap with neuromast hair cells, notably including the axonemal gene *pierce1* (si:ch211−248e11.2) and the molecular chaperone *hsp90aa1.2*. Cross-referencing with a published neuromast scRNA-seq dataset (Baek et al., 2022, PMID: 35316618) confirmed that these genes are preferentially expressed in hair cells rather than support cells, suggesting shared sensitivity to cytoskeletal or cellular stress (**Figure 2 for reviewer**).

Given the limited evidence for conserved mechanosensory pathways between neuromast hair cells and endothelial cells, and to avoid overinterpretation, we have moved the neuromast DASPEI/BODIPY data to Supplementary Information and revised the manuscript text. We have also expanded the Discussion to highlight other established endothelial mechanosensors, including caveolae and junctional complexes as potential contributors to the flow-sensitive CVP endothelial phenotypes. We believe these revisions clarify the scope and better align the manuscript’s focus with endothelial-specific mechanobiology.

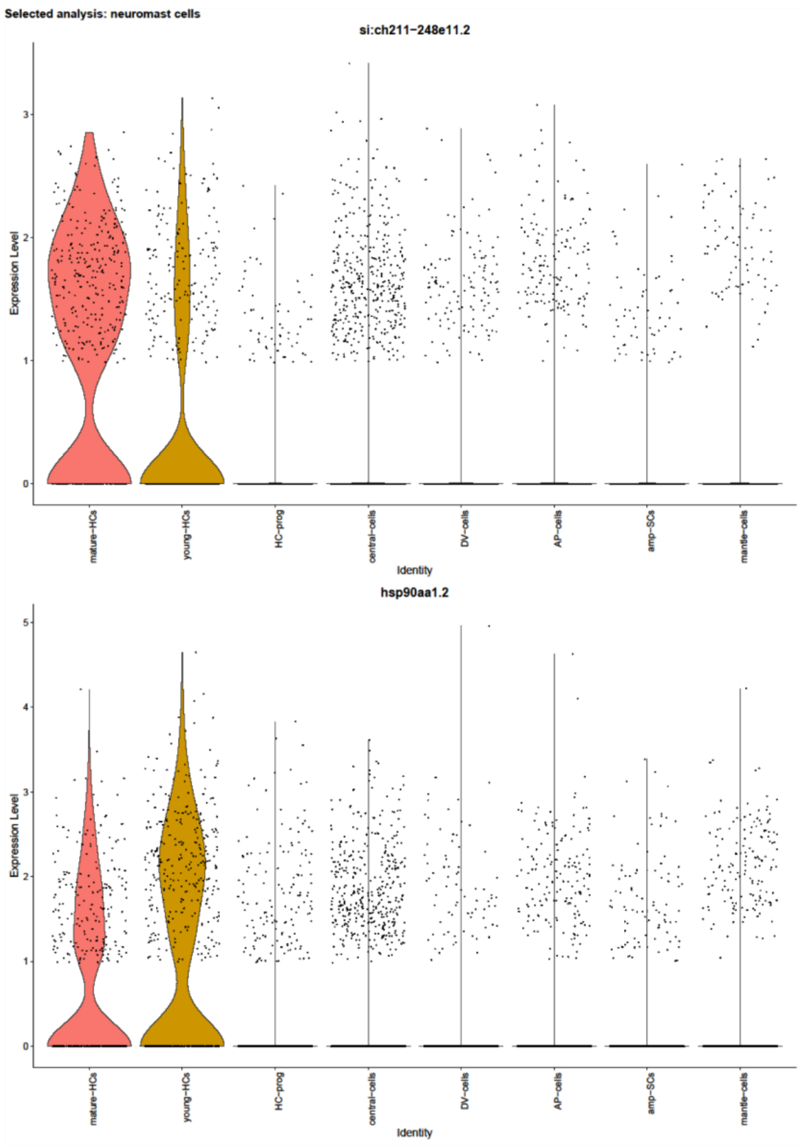

Figure 2 for reviewer: Neuromast scRNA-seq expression profile of topmost significantly enriched genes in the *hsp70+* endothelial cell subset in *ldlr-/-* embryos (dataset from Baek et al., 2022, PMID: 35316618). Violin plot showing expression levels of *si:ch211−248e11.2* (*pierce1*, top) and *hsp90aa1.2* (bottom) in each neuromast cell type, indicating expression enrichment primarily in hair cell populations. HC, hair cell. Prog, progenitor. DV, dorsal-ventral. AP, anterior-posterior. Amp, amplifying.

3. In Figure 3A and S3, the proportion of stressed endothelial cells (EC) is very limited. In the HAEC scRNAseq data, the stressed EC cluster (HSP70+, HSS 5) also exhibited a limited number on the UMAP (Figure S9). Given that physiological shear stress typically promotes athero-protective and anti-inflammatory phenotypes in ECs. It is unclear why only a small subset exhibits opposite effects in response to HSS in HAECs or CVP in zebrafish. The manipulation of HSP70, which is expressed in only a small portion of the EC population, leads to vascular regeneration and inflammation. The manuscript should clarify how targeting this minor fraction of trunk ECs influences such significant phenotypic changes.

We thank the reviewer for raising this important conceptual question. We agree that the relatively small proportion of stressed endothelial cells identified in both zebrafish and HAEC scRNA-seq datasets is a key feature of our findings and central to the biological interpretation of the study.

In both systems, physiological flow promotes homeostatic phenotypes. However, our recent zebrafish data support that in genetically susceptible and geometrically complex vascular regions, a minor, spatially restricted population of endothelial cells enters an acute stress state, characterized by the induction of *hsp70*. More specifically, in *ldlr*-/- zebrafish, *hsp70*+ endothelial cells arise preferentially within regions of disturbed physiological flow in the dorsal caudal venous plexus and coincide with defective angiogenic remodeling, loss of

endothelial ciliogenesis and increased vascular apoptosis (**Figure 1D-E**). Similarly, in HAECs exposed to physiological shear stress, scRNA-seq reveals transcriptional heterogeneity, with a small subset of cells exhibiting a stress-associated expression program, despite the overall protective flow environment. Cross-species comparison of zebrafish and HAEC data highlighted conserved flow-coupling of *HSP70* and *DNAJB1* (cytoplasmic molecular chaperones) within these stressed subpopulations.

We propose that these discretely stressed endothelial cells act as local regulators of vascular dysfunction, exerting disproportionate effects on angiogenic remodeling. Consistent with this, pharmacological modulation of Hsp70, despite its expression in a minority of cells, either exacerbates or rescues vascular remodeling phenotypes, linking *hsp70+* endothelial cells to these vascular outcomes. This is in line with growing evidence from vascular biology that small, transcriptionally distinct endothelial subsets can substantially influence vessel-level outcomes (reviewed by Trimm and Red-Horse, 2022, PMID: 36198871 and Becker et al., 2023, PMID: 35179567).

We have revised the Results and Discussion to more clearly articulate this concept, emphasizing endothelial heterogeneity, spatial flow disturbances and the impact of stress-responsive endothelial subsets. We have also included a summary schematic (**Figure 7**) to illustrate how the early emergence of *hsp70+* endothelial cells regulates developmental vascular susceptibility in *ldlr*-/- zebrafish.

4. It is difficult to ascertain from the current data how these stressed ECs play a crucial role in ldlr-/- zebrafish development.

We agree that the role of the stressed *hsp70+* endothelial cell population in *ldlr*-/- zebrafish should be more clearly articulated. We have therefore revised the Results and Discussion to define how the emergence of these cells contributes to the developmental phenotypes observed.

Our data support a model in which *ldlr* deficiency leads to early endothelial apoptosis and ER stress detectable at 1dpf, during sprouting angiogenesis. As circulation is established, regions of disturbed physiological flow within the CVP give rise to a spatially restricted subpopulation of endothelial cells that activate a cytoplasmic molecular chaperone stress response marked by *hsp70* induction at 2 dpf. Importantly, genetic and pharmacologic data suggest that flow- and cell stress-induced *hsp70+* endothelial cells alter angiogenic remodeling, inhibit CVP ciliogenesis and limit vascular apoptosis, reflecting an attempt to resolve cellular stress but failing as endothelial stress phenotypes persist at 3 dpf.

We have incorporated this framework into the revised manuscript, and the summary schematic (**Figure 7**) visually integrates the temporal relationship between ER stress, endothelial apoptosis, *hsp70+* endothelial emergence, angiogenic remodeling and downstream vascular and inflammatory defects. Additional new analyses of CVP hemodynamics and the spatial localization of *hsp70+* endothelial cells supports this model and are described in response to point 5.

5. In the discussion, the authors mention that the CVP is exposed to disturbed flow or lower shear stress. Therefore, reduced flow in CVP is not necessary. Additionally, the comparison between physiological flow in HAECs and CVP EC is not clearly matched. This point should be addressed.

We thank the reviewer for this important point and we have now clarified both the hemodynamic characteristics of the CVP and the rationale for our flow comparisons. Indeed, the CVP is intrinsically heterogenous and contains regions exposed to disturbed or lower shear stress flow, particularly at vascular loops and bifurcation points. However, the CVP as a vascular bed also contains regions exposed to relatively higher flow. To directly address this, we performed new live imaging analyses using of Tg(*gata1:dsRed*) zebrafish to track individual red blood cells (RBCs) through distinct CVP subregions under physiological and reduced-flow conditions (**Figure 1D**, **Figure S11**).

These analyses reveal that in *ldlr*-/- embryos, disturbed flow is spatially localised, rather than uniform across the CVP. Specifically, the dorsal caudal venous plexus (dCVP) exhibits significantly reduced RBC velocities compared to the ventral CVP (vCVP), and the dCVP coincides with increased vascular looping, enrichment of *hsp70+* endothelial cells, elevated endothelial apoptosis and reduced ciliogenesis (**Figure 1D-E**). In contrast, the vCVP retains higher RBC velocities and fewer stress-associated phenotypes. Importantly, experimentally reducing blood flow preferentially amplifies low-velocity through the dCVP, with the vCVP minimally utilized,

indicating that reduced flow has region- and dose-dependent effects rather than uniformly recapitulating CVP hemodynamics (**Figure S11A-B**).

Together, these data support a model in which physiological blood flow through a structurally disturbed CVP, rather than reduced flow per se, promotes acute endothelial *hsp70* induction in *ldlr*-/- embryos. We have now clarified this distinction in the Results and Discussion.

We also acknowledge that physiological flow in the zebrafish CVP *in vivo* is not directly equivalent to physiological shear stress applied to HAECs *in vitro*. Our intent here is to compare flow-dependent transcriptional responses across model systems. Despite intrinsic differences, we observe conserved flow-associated induction of cytoplasmic stress chaperones, HSP70 and DNAJB1, in both zebrafish endothelial cells and HAECs. We have now stated the limitations and interpretive framework of this cross-model comparison in the revised manuscript.

6. Please review each dataset for completeness; some parts are missing quantifications or representative images. Providing these details will ensure the data supports the results more robustly.

We thank the reviewer for highlighting this point. We have reviewed all datasets and figures for completeness and consistency. Where applicable, we have added missing quantitative labels, statistical annotations and included representative images to ensure all reported phenotypes are directly supported by the presented data. These additions have been incorporated into both the main figures and supplementary materials.

7. It vascular phenotype of ldlr-/- zebrafish between 2-3 dpf. But the vascular phenotype including hsp70l expression has recovered after 5 dpf. Please discuss this time-dependent manner effects.

We thank the reviewer for raising this point. We agree that the transient nature of CVP and *hsp70+* endothelial phenotypes is a key aspect of our findings and warrants discussion. We have revised the Discussion to address the time-dependent progression and limited resolution of these phenotypes.

Our data indicate that *ldlr*-/- zebrafish exhibit an acute window of endothelial vulnerability between 1-3 dpf, during which upstream ER stress and endothelial apoptosis, followed by altered flow dynamics in the CVP and subsequent emergence of *hsp70+* endothelial cells converge to disrupt angiogenic remodeling. By 5 dpf vascular architecture and *hsp70+* endothelial expression are largely normalized, yet at this stage *ldlr*-/- zebrafish display elevated endothelial apoptosis, while also showing beginning signs of systemic hypercholesterolemia.

We interpret this temporal restriction as evidence that *hsp70+* endothelial cells represent an early, stress-responsive endothelial state. This transient and converging response at 2 dpf is sufficient to perturb developmental vascular remodeling during a critical window. We have now incorporated this interpretation into the revised manuscript and summarized it schematically (**Figure 7**).

8. For the in vitro experiments, have you considered using HSP70 siRNA or an inhibitor to inhibit the effects of HSP70?

We thank the reviewer for this experimental suggestion and agree that genetic or pharmacological inhibition of HSP70 *in vitro* represents a valuable complementary approach. In the present study, our *in vitro* HAEC experiments were designed to assess flow-dependent transcriptional heterogeneity, rather than to establish causality, which we addressed primarily using *in vivo* approaches in zebrafish.

Importantly, HSP70 inhibition was tested extensively *in vivo*, where we demonstrate rescue of aberrant vascular remodeling in *ldlr*-/- embryos (**Figure 5**), providing evidence that endothelial *hsp70* activity is responsible for the observed developmental vascular abnormalities.

While siRNA-mediated knockdown or pharmacological inhibition of HSP70 in HAECs would be informative, these approaches are technically challenging under prolonged shear stress conditions and may introduce confounding effects related to global proteostasis disruption. We will consider the suggested manipulations by altering experimental conditions in future mechanistic studies.

9. The ldlr-/- EC phenotypes have recovered after 4-5 dpf. Is this due to the change of hemodynamics during the

development? For example, flow in PCV is higher at 5 dpf vs. 2 dpf? It would be great to compare hemodynamics at different time points.

We thank the reviewer for this insightful suggestion. To address whether developmental changes in hemodynamics may contribute to partial phenotype resolution, we have now quantified RBC velocity in the caudal vein and dorsal aorta at both 2 dpf and 5 dpf (**Figure S2D**).

These analyses demonstrate a developmental increase in mean RBC velocity between 2 dpf and 5 dpf in both vascular beds (**Figure S2D**). This increase in flow coincides temporally with normalization of endothelial *hsp70* expression and recovery of CVP/CV architecture at 4-5 dpf, supporting the interpretation that maturation of hemodynamic forces may contribute to endothelial *hsp70* resolution.

Importantly, this normalization is selective, as increased endothelial apoptosis and ER stress persist at 3 dpf, with endothelial apoptosis and LDL-cholesterol also elevated in *ldlr*-/- zebrafish at 5 dpf (**Figure 1B, Figure S1B**). Thus, our data suggest that distinct abnormalities arise at different developmental stages, with early flow-sensitive endothelial stress preceding later cholesterol-associated phenotypes.

We have clarified this temporal and mechanistic distinction in the revised Results and Discussion.

10. Please carefully review all figures to ensure completeness, as some quantifications, representative images, and comparisons are missing. Additionally, the data presentation in the main manuscript should be reorganized to improve readability. Currently, the layout is overly dense and confusing.

We thank the reviewer for this important comment. We have reviewed all figures and addressed missing quantifications, representative images and key comparisons, including the addition of newly acquired data where appropriate. We have also grouped related datasets to better align with the Results text and moved secondary or supporting analyses to Supplementary Information.

11. Please explain and provide more details on scRNAseq data analysis such as cell markers, statistics, etc. Specifically, describe how you annotated the cell types in each dataset, especially how to segregate 3 different EC populations.

We thank the reviewer for this request and agree that additional methodological detail will improve transparency of the key scRNA-seq analyses. We have expanded the Methods section accordingly and provided supporting data.

We now include datasheets listing the most significantly enriched genes for each annotated cluster, including $\log_2$ fold change values and adjusted significance statistics. Cell type annotation was performed by ranking genes within each cluster by $\log_2$ fold change and statistical significance, followed by cross-referencing these gene signatures with published zebrafish scRNA-seq atlases and validation against the ZFIN database.

Neural cell-associated clusters were annotated using reference datasets from Raj et al., 2020 (PMID: 33068532), Baek et al., 2022 (PMID: 35316618) and Farnsworth et al., 2020 (PMID: 31782996). All remaining cell populations were annotated using established zebrafish atlases and vascular datasets from Farnsworth et al., 2020, Metikala et al., 2021 (PMID: 34234366) and Gurung et al., 2022 (PMID: 35906287). A visual summary of the top enriched genes across clusters is provided in the supplementary heatmap (**Figure S8A**).

Endothelial cell populations were annotated in a stepwise manner. All three EC clusters were first identified based on enrichment of canonical pan-endothelial markers (*cdh5, kdrl,* and *tie1*). These ECs were then subdivided based on anatomically informative gene expression patterns. One cluster corresponded to cranial endothelial cells, characterized by enrichment of genes associated with cranial vasculature and cytoskeleton (e.g., *edn1, foxc1b,* and *adarb1a*). A second cluster represented trunk vessel endothelial cells expressing markers associated with the caudal venous endothelium (*sele, stab1, mrc1a*) and arterial/intersegmental vessels (*notchl, esm1,* and *arhgef9b*).

A third endothelial population, present exclusively in *ldlr*-/- embryos, was identified as a stressed EC subpopulation within the trunk EC cluster. This population was defined by maximal and selective enrichment of the four *hsp70* orthologs (*hsp70l, hsp70.1, hsp70.2,* and *hsp70.3*, **Figure S8B**), which were minimally expressed in all other clusters. This annotation strategy is now explicitly described in the revised Methods.

12. The flow study in zebrafish is well-designed. For the scRNA seq, the authors used tnnt2a MO to minimize off-target effects, and used Control MO. In the following violin plot of hsp70l expression (Figure 3B), it shows that in the "normal ECs", hsp70l is higher in ldlr KO embryos under reduced flow as compared to normal flow. The authors should explain the indication of this observation, as the other data suggests that reduced flow leads to reduced hsp70l activation. Providing a UMAP expression plot of hsp70l and annotations of the two EC types and experimental groups would help clarify this.

We thank the reviewer for carefully noting this discrepancy. We agree that this observation required clarification and have now addressed it in the Results and Supplementary Figures.

Under reduced flow conditions, scRNA-seq clustering did not resolve a distinct *hsp70*+ trunk endothelial cell subpopulation in *ldlr*-/- zebrafish. Instead, we observed a modest elevation of *hsp70l* expression distributed across the broader "normal" trunk EC population. In contrast, under regular physiological flow, *ldlr*-/- embryos exhibited a clearly separable trunk EC cluster characterized by strong, focal enrichment of *hsp70* orthologs.

We interpret this to indicate that reduced flow induces a low-level, diffuse endothelial stress response that raises baseline *hsp70l* expression but is insufficient to drive the emergence of a discrete stressed EC state. Physiological flow, in combination with *ldlr* deficiency, appears necessary to concentrate stress signaling spatially and transcriptionally, resulting in the formation of a distinct *hsp70*+ trunk EC subpopulation.

As suggested, we have added UMAP expression plots showing *hsp70l* distribution across trunk endothelial cells under the three flow conditions and between genotypes (**Figure S14**), which clarifies this distinction. To independently validate these findings, we have also now performed whole-mount fluorescence RNA in situ hybridization (FISH) for *hsp70* in *ldlr*+/+ and *ldlr*-/- embryos exposed to reduced and static flow (**Figure 3D**), which corroborates the scRNA-seq results.

Together, these data reconcile the violin plot observation and support a model in which physiological flow enables focal *hsp70*+ EC specification in *ldlr*-/- embryos, whereas reduced flow produces a lower grade and more generalized stress response.

13. The authors presented in situ hybridization (ISH) of hsp70l mRNA on BDM vs treated ldlr KO embryos. Would it be possible to perform hsp70l ISH on the tnnt2a M-treated group? the reason is that BDM is known to have many off-target effects, and it is more representative of "static flow", not reduced flow (Figure S5B). Staining on the tnnt2a MO groups would be a better validation of their scRNA seq.

We agree with the reviewer that *hsp70* FISH in *tnnt2a* morpholino-injected embryos provides a more appropriate validation of the scRNA-seq findings than BDM treatment alone. We have now performed FISH for *hsp70* in *tnnt2a*–injected *ldlr*+/+ and *ldlr*-/- zebrafish embryos exposed to reduced and static flow.

These data, now included in the revised manuscript (**Figure 3D**), show that both *ldlr*+/+ and *ldlr*-/- embryos exposed to reduced flow display similarly low numbers of *hsp70*+ endothelial cells. This level is significantly lower than that observed in *ldlr*-/- embryos under physiological flow, yet higher than in embryos exposed to static flow.

Together, these results validate the scRNA-seq observations and support a flow-dependent, dose-responsive induction of endothelial *hsp70* expression in *ldlr*-/- zebrafish.

14. hsp70l showed elevation in ldlr KO at 2 dpf but then returned to normal at 3 dpf (Figure 2D). This is an intriguing phenomenon, as 2-3 dpf is a major window for cardiac development, and a significant increase of cardiac output (flow). I would recommend the authors also look into hsp70l expression in tnnt2a-treated WT and ldlr KO embryos at 3-4 dpf. This may reveal more about the relationship between hsp70l and flow.

We thank the reviewer for highlighting this developmental window and agree that the transient elevation of endothelial *hsp70* at 2 dpf coincides with a period of increasing cardiac output and changing hemodynamics.

We considered extending flow-reduction experiments into the 3-4 dpf window using *tnnt2a* morpholino treatment. However, zebrafish lacking cardiac contraction develop oedema and reduced viability from 3 dpf onwards, making it difficult to distinguish direct effects of altered flow from secondary systemic toxicity. For this reason, *tnnt2a* morphants are not well suited for assessing endothelial stress responses beyond embryonic stages.

Our newly added hemodynamic analyses demonstrate a marked developmental increase in RBC velocity between 2 and 5 dpf (**Figure S2D**), coincident with normalization of endothelial *hsp70* and recovery of CVP architecture. Together with the spatial CVP flow analyses at 2 dpf (**Figure 1D–E**), these data support a model in which endothelial *hsp70* induction is restricted to an early, flow-sensitive developmental window.

We have clarified this interpretation in the revised Discussion. While alternative models of chronically reduced flow may help address this question in future studies, such experiments fall beyond the scope of the present work.

Minor:

1. For Figure 1C, please include images from ldlr+/+ for the xz and yz planes for a more comprehensive comparison. Also, the representative images for cilia are not clear. We have now included the ldlr+/+ xz and yz plane images for direct comparison and added higher magnification insets to more clearly visualize CVP cilia and adjacent vascular loop structures in both genotypes.
2. In Figure 1D, please provide quantification data for the BODIPY staining to support the visual observations made from the staining results. We have now included quantification of the BODIPY staining alongside the representative images to support the visual observations (**Figure S6**).
3. Please provide representative images for 4 and 5 dpf. Also, indicate the dorsal aorta (DA), intersegmental vessels (ISV), and posterior cardinal vein (PCV) in the representative images if these structures are visible. If these structures are not currently shown, please include images that display them. We have now included representative images of the trunk vasculature at 4 dpf and 5 dpf (**Figure S1C, Figure S2B**). In both the main and supplementary figures, the dorsal (DA), intersegmental vessels (ISVs), dorsal longitudinal anastomotic vessel (DLAV), parachordal lymphatic (PCL) and caudal venous plexus/caudal vein (CVP/CV) are labelled.
4. Additionally, it is unclear whether the stressed ECs are derived from trunk ECs. We have clarified in the Results and figure legend that the *hsp70*+ endothelial cell subpopulation is derived from the main trunk EC population, confirming its origin within the trunk vasculature.
5. Additionally, please interpret the Figure 3F well. We have now clarified the interpretation of Figure 3F in both the Results and figure legend, highlighting the key observations and their relevance to *hsp70*+ endothelial cells.
6. For Figure 2E, relative mRNA expression is generally set the value as 1 for the control. Also, it would be better to have the dot plot and bar graph. We appreciate the reviewer's point that RT-qPCR data are often presented with experimental groups normalized to corresponding control. In this case we aimed to highlight developmental changes in gene expression in both ldlr+/+ and ldlr-/- zebrafish. Normalizing each ldlr-/- timepoint to the stage-matched ldlr+/+ zebrafish (ldlr+/+ = 1) would emphasize mutant deviations but mask the developmental expression changes observed in ldlr+/+ zebrafish. Retaining the current visualization allows transparent comparison of both genotype- and stage-dependent expression changes for the cell stress response genes analyzed.
7. In Figure 4, would it be better to replace the bottom of panel A with Figure S9? We thank the reviewer for this formatting suggestion. The bottom panel of Figure 4A illustrates the overall scRNA-seq analysis workflow, while Figure S9 (now **Figure S16**) provides detailed cluster analysis, including UMAPs of clusters, cluster proportions and heatmaps of upregulated genes in each cluster. We believe keeping these data in a stand-alone supplementary figure better serves readers who wish to examine the HAEC subpopulations in detail, while maintaining a clear and interpretable main figure.
8. Using an activator or inhibitor of Hsp70 in ldlr+/+ and ldlr-/- zebrafish is a key experiment in this study. Thus, it has to be carefully dissected out the results in Figure 5. Additionally, some data is not consistent with results in the Figure 5. For example, Hsp70 inhibitor (VER) or CRISPR increased apoptosis in both ldlr+/+ and ldlr-/-, whereas VER decreased the CVP pillar and increased CVP cilia # in Figure 5D. Also, there are no effects of VER on CVP pillar and cilia in ldlr+/+ fishes in Figure 5D. Please address this discrepancy. We thank the reviewer for highlighting the need to clarify the phenotypic relationships in Figure 5. The apparent discrepancy may arise from measurements reflecting distinct vascular processes: (1) endothelial apoptosis and (2) CVP remodeling and related ciliogenesis. These processes differ both in baseline state and their sensitivity to Hsp70 modulation. Ldlr-/- embryos exhibit elevated baseline vascular apoptosis, while VER increases apoptosis in ldlr+/+ trunk vessels, but does not further elevate apoptosis in ldlr-/- embryos, likely due to a ceiling effect. In contrast, CVP remodeling is a flow-dependent phenotype and occurs independently of elevated trunk-vessel apoptosis. Consequently, VER selectively rescues CVP ciliogenesis and loop formation in ldlr-/- embryos, while having minimal effect in ldlr+/+ embryos which lack a baseline CVP phenotype. We have now clarified these points in the Results text, revised Figure 5 panels to improve interpretability and included some data in the supplementary materials to simplify the main figures.

We thank the reviewer again for the helpful and detailed suggestions in clarifying our results figures and associated text.

Reviewer #4 (Remarks to the Author):

Noteworthy results
The authors use a new zebrafish ldlr knockout model of familial hypercholesterolemia to study endothelial cells at early stages before cholesterol becomes elevated. They show that there are stressed endothelial cells in early development and identified a gene program leading to hsp70 expression. Hsp70 expression was only induced with physiological flow, a similar result to human endothelial coronary artery cells where hsp expression was flow dependent. Ciliogenesis and intussusceptive angiogenesis are affected in the zebrafish model of ldlr loss, and apoptosis is induced in endothelial cells. If hsp70 is inhibited or activated in ldlr fish, there is a corresponding rescue or worsening of apoptosis, angiogenesis and ciliogenesis suggesting hsp70 marks cells that behave abnormally. Downstream angiogenesis defects and alteration in immune cells as seen. Overall, the authors suggest a novel hypothesis, that early abnormalities that give rise to endothelial cell dysfunction and promote inflammation prior to the emergence of hypercholesterolemia.

Significance and novelty
Overall, the data are novel. No other model (fish or mouse) has shown similar early endothelial defects prior to cholesterol misregulation. The connection to hsp70 upregulation is also novel. Findings on immune dysregulation (neutrophils), and ciliogenesis defects are also unique. Overall, there is a substantial novelty in the data and approach of the manuscript.

We thank the reviewer for their careful evaluation, positive remarks and valuable feedback on our study. In the revised manuscript, we have addressed the specific points raised by the reviewer, as detailed in our point-by-point responses below.

There are several points that must be addressed:

1. The reviewer is left wondering why scattered cells upregulate hsp70, particularly in light of the later findings with blood flow. There is a missed opportunity to understand where the hsp70 expressing cells arise in vivo. Blood flow in the CVP is heterogeneous, faster in the larger vessels, slower in the smaller vessels. Flow will also change at bifurcations. The localization of the hsp70 expressing cells with respect to the architecture would be very informative. Are the hsp70 expressing cells only in the smallest vessels or large ones? Are they in remodelling vessels or in vessels that will be maintained? How does their location relate to blood flow? Do they have altered morphology? Understanding this relationship would strengthen the claim that blood flow interrelates with hsp70 expression downstream of a stress program.

We thank the reviewer for their insightful comments and we agree that defining the spatial relationship between *hsp70*+ ECs, vascular architecture and blood flow is critical for interpreting their role in CVP remodeling. To address this, we acquired additional live imaging datasets using Tg(*gata1:dsRed*) zebrafish and performed detailed tracking of individual red blood cells (RBC) moving through the CVP in *ldlr*+/+ and *ldlr*-/- embryos (**Figure 1D**, **Figure S2D**, **Figure S11**).

The reviewer correctly noted that blood flow is heterogenous within the CVP. We identified that the ventral CVP subregion (vCVP) was exposed to high RBC velocities, whereas the dorsal CVP subregion (dCVP) was exposed to lower RBC movement velocities. Notably, the dCVP of *ldlr*-/- embryos exhibited a further reduction in RBC velocity compared to their *ldlr*+/+ counterparts (**Figure 1D**), prompting us to perform more detailed spatial vascular phenotyping.

We reanalysed our confocal datasets and quantified the number of vascular loops, apoptotic ECs, endothelial cilia and *hsp*70+ ECs separately within the dCVP and vCVP sub-vessels between the genotypes. These analyses revealed that the dCVP is the primary site of altered vascular remodeling in *ldlr*-/- embryos, characterized by increased vascular loops, elevated numbers of *hsp70*+ ECs and apoptotic ECs, and reduced endothelial cilia (**Figure 1E**, **Figure 2D**). In contrast, the vCVP in both *ldlr*+/+ and *ldlr*-/- embryos, exhibited higher RBC velocities and significantly fewer vascular loops, apoptotic ECs and cilia, suggesting relative protection from aberrant remodeling (**Figure 1D-E**).

To further evaluate the relationship between *hsp70*+ ECs and altered vascular remodeling in *ldlr*-/- zebrafish, we performed linear regression analyses and identified significant positive correlations between (1) dCVP *hsp70*+ ECs and dCVP loop number and (2) *hsp70*+ EC number and overall CVP diameter (**Figure S9**). Together, these data demonstrate that *hsp70*+ ECs localise preferentially to disturbed flow, remodeling CVP subregions, strengthening the conclusion that blood flow heterogeneity spatially interrelates with endothelial stress responses in *ldlr*-/- zebrafish. We have incorporated these new analyses into the main/supplementary figures and conclusions of the revised manuscript.

Is hsp70 expressed downstream of mechanosensory pathways?

To address whether *hsp70* is expressed downstream of mechanosensory pathways, we have expanded our scRNA-seq analysis and now provide additional supplementary datasheets detailing gene enrichment (ranked by log□FC and significance) for each annotated zebrafish cluster. Within the *hsp70*+ EC cluster identified in *ldlr*-/- embryos, we detected enrichment of *klf2a* and *klf2b* among the top 27 genes (**Figure S8B**), both of which are flow-sensitive transcription factors induced by shear stress.

These transcription factors are activated from 1 dpf, at the onset of cardiac contractility, whereas endothelial *hsp70* induction occurs at 2 dpf and is spatially restricted to the CVP of *ldlr*-/- embryos. This temporal relationship supports the interpretation that *hsp70* expression occurs downstream of the main mechanosensory pathways.

Furthermore, we provide additional fluorescence *in situ* hybridization experiments for *hsp70* in zebrafish exposed to reduced and static flow, which demonstrated that *ldlr*+/+ and *ldlr*-/- embryos exposed to reduced flow display similarly low numbers of *hsp70*+ ECs, significantly fewer than *ldlr*-/- embryos exposed to physiological flow, while more than *ldlr*-/- embryos exposed to static flow (**Figure 3D**). RBC tracking under reduced flow conditions further revealed preferential utilization of the dCVP with markedly reduced RBC velocities (**Figure S11A-B**).

Together, these data support a flow gradient-dependent requirement for endothelial *hsp70* induction in *ldlr*-/- zebrafish, consistent with *hsp70* acting downstream of mechanosensory pathways. We have clarified these points in the Results and Discussion. While comprehensive genetic dissection of individual mechanosensors is beyond the scope of the present study, the zebrafish model offers a tractable platform for future investigation of these pathways.

Is ciliogenesis disrupted in all endothelial cells, or only the ‘stressed’ cells? Cell level resolution would be informative as it could indicate that the most severely stressed cells that are scattered throughout vessels are also the same cells that show other phenotypes. If all cilia are affected, why does rescue with VER, which would affect cells expressing hsp70 have an effect?

To determine whether disrupted ciliogenesis is a global endothelial phenotype or restricted to stressed EC populations, we quantified endothelial cilia across all major trunk vessels at 2 dpf. We found no significant differences in ciliogenesis between *ldlr*+/+ and *ldlr*-/- embryos in the the DLAV, ISVs, PCL or DA (**Figure 1C**), indicating that impaired ciliogenesis is not a pan-endothelial effect.

Instead, the CVP, and particularly dCVP, emerged as the primary site of reduced endothelial ciliogenesis in *ldlr*-/- embryos (**Figure 1C, Figure 1E**). The CVP normally contains the highest density of endothelial cilia at this developmental stage, and our new data show that these cilia are spatially concentrated in the same subregion in *ldlr*+/+ embryos that exhibit decreased ciliogenesis and increased *hsp70*+ ECs, vascular loops and endothelial apoptosis in *ldlr*-/- embryos (**Figure 1E**).

We agree that single-cell-level co-localization of *hsp70* expression and cilia loss would be highly informative. However, due to technical limitations in performing combined staining while preserving sufficient resolution to visualize endothelial cilia, we were unable to reliably assess these features within the same cells. Despite this limitation, the spatial convergence of these phenotypes within the dCVP supports the interpretation that disrupted ciliogenesis is associated with localized endothelial stress rather than a uniform vascular defect.

In Figure 5D there does not appear to be significance between DMSO for LDLR positive and mutant cells. Is this an oversight(i.e. not placing significance bars on the graph) as in earlier figures they are significantly different?

Thank you for bringing this to our attention. We have now added this statistical comparison to the graph.

2. Images of critical data to support claims (intussusceptive pillars) are too low resolution to view the underlying data. Better resolution images are imperative for all data supporting a claim that there is altered intussusceptive angiogenesis. Because these are not visible from the images provided, it makes the reviewer wonder whether the authors correctly counted pillars. An excellent example of what the pillars should look like can be found in figure 2 of this paper: https://doi.org/10.1038/s41598-018-27791-6
Insets must be shown to support every claim of decreased intussusceptive angiogenesis. Methods of measurement (where, when, how) should be briefly outlined in the results, and marked on at least one of the images. The pillars are within single vessels and are distinguished from loops. How was the largest pillar identified.
Cilia are also similarly shown at too low resolution to support the data.

We agree with the reviewer that higher resolution images are important to support claims regarding CVP vascular remodeling and endothelial cilia. We have now added higher resolution insets throughout the manuscript (**Figures 1, 3 and 5**), specifically highlighting CVP loops and endothelial cilia.

We appreciate the reviewer's point about CVP pillars and bringing the cited study to our attention. It seems the definition of zebrafish CVP intussusceptive pillars or vascular loops/columns can vary with studies quoting different sizes e.g., ~3 μm diameter (Karthik et al., 2018, PMID: 29959335) or greater than 9 μm^2 by area (Eisa-Beygi et al., 2023, PMID: 37128914). In many studies the size of the CVP hole is not specifically defined yet still referred to as a pillar or loop or column. As a result, CVP intussusceptive angiogenesis is often confused with remodeling/pruning angiogenesis. As the reviewer rightly points out, the cited study by Karthik et al., 2018 is currently one of the best characterizations of CVP pillars in zebrafish, and defines the pillars as having diameters approximately ≤ 2.5 μm, with holes greater than 2.5 μm considered to be meshes (large vascular pillars), which are perhaps assumed to be vascular loops in other studies (Goetz et al., 2014, PMID: 24561257; Wen et al., 2021, PMID: 34319989; Eisa-Beygi et al., 2023). To use more precise nomenclature for larger CVP pillars, we have changed the naming convention of CVP pillars to CVP loops throughout the manuscript, based on how we have defined their size (a hole in the vascular plexus of area greater than or equal to 6 μm^2 and intraluminal diameter greater than or equal to 4 μm). Correspondingly, we have replaced textual references of "intussusceptive angiogenesis" throughout the manuscript to vascular remodeling/pruning or remodeling angiogenesis.

We have updated the Methods section to describe in more detail how CVP loops were identified and the criteria for measurements. The main representative image insets are now also annotated to indicate CVP loops. The largest CVP loop was identified by measuring the along the intraluminal perimeter of the largest resolvable opening in the CVP (image processed as maximum intensity projection of 3D z-stack), within the defined the region of interest. Finally, we have similarly improved the presentation of endothelial cilia to support associated quantifications (**Figure 1** and **Figure 5**). We thank the reviewer for prompting these clarifications and corrections which have strengthened the interpretability of the vascular remodeling analyses.

3. The authors used huAECs under different flow conditions to show that Ldlr and Hsp70 orthologs (HSPA1A/B) and other stress genes are downregulated with reduced flow, matching the fish data, although there is heterogeneity in the samples. There is very little data on the HuAECs from the commercial supplier, but were all HuAECs from a single donor? How do the authors know that the heterogeneity in the scRNAseq is not just due to genetic background effects? It should be clarified in the methods whether different batches/donors were used.
We thank the reviewer for raising this important point regarding potential sources of transcriptional heterogeneity in the HAEC scRNA-seq data. All primary HAECs used for the flow experiments were isolated from a single donor in a single batch. Importantly, all experimental flow conditions were applied using this same HAEC population, indicating the transcriptional heterogeneity observed is most likely not because of donor-to-donor variability or genetic background differences. We have now added this information to the methods section.

4. Similar to the above point about flow and heterogeneity in fish, flow devices may have slightly uneven flow. In situ, are the cells at the edge (or middle of the device) more likely to express hsp70 as a response to differential flow?

We expect there to be a degree of transcriptomic heterogeneity in endothelial populations, as has been reported in recent HAEC scRNA-seq studies (Adelus et al., 2024, PMID: 38578680). We also acknowledge that minor shear stress gradients may exist near the peripheral edges of the rectangular Ibidi flow slide. However, in our experiments, the vast majority of HAECs exposed to flow were located within the central region of the Ibidi slide.

The flow conditions were established following the manufacturer's validated protocol for the IbidiTreat u-slide I Luer$^{0.8}$ (https://ibidi.com/img/cms/downloads/an/AN11_Shear_stress.pdf, page 9), which indicates that homogeneous shear stress is achieved across the central field of the chamber. We have now stated this in the Methods section. Based on these conditions, we would therefore expect *HSP70* induction to predominantly reflect flow-dependent transcriptional responses rather than positional effects within the device.

5. The HuAECs are wildtype. The relevance to ldlr knockout heterogeneity is questionable since in ldlr mutant fish cells under flow, hsp70 is upregulated, but in the human wildtype cells, HSP1 is uniformly expressed in physiological flow. Why are the human cells behaving differently?

We agree that the human HAECs data do not fully recapitulate the genetic context of *ldlr* loss-of-function in zebrafish. Our intent in including the HAEC data was not to model LDLR deficiency per se, but rather to test whether flow-dependent endothelial stress responses, particularly HSP70-associated programs, are conserved across species and experimental systems.

We acknowledge that physiological flow in the zebrafish CVP *in vivo* differs from shear stress applied to HAECs *in vitro*, including differences in vessel type (venous vs arterial), developmental state and biomechanical environment. An additional potential reason for the discordant extent of the transcriptional heterogeneity in wt HAECs compared to wt zebrafish ECs could be due to capturing approximately 2-fold more human ECs compared to embryonic zebrafish ECs. These differences combined perhaps all contribute to the more uniform *HSP70* expression observed in HAECs under physiological shear compared to the *ldlr*-/- zebrafish endothelium.

Despite these differences, we observe partial conservation of flow-responsive cytoplasmic stress chaperones between zebrafish and human ECs, namely *HSP70* and *DNAJB1* (detailed in our response to reviewer 3 point #3). We have now highlighted these model-specific considerations in the Results text.

a. What happens to the gene expression when LDLR is reduced in the human cells (siRNA)?

This is an excellent question that we hope to address in future mechanistic studies. As the primary focus of the current study is related to understanding *in vivo* vascular abnormalities arising during early development, we prioritized experimental efforts toward flow-dependent mechanistic dissection in the *ldlr*-/- zebrafish model.

b. An alternative to the human data would be to analyze the hsp70 expression in ldlr mutant embryonic mice. This is not necessary for publication in our opinion, but would dramatically increase the impact of the findings.

We agree that extending our findings to additional vertebrate models, such as *Ldlr* mutant embryonic mice, could further strengthen the translational impact of this work. However, we were unable to identify prior studies examining vascular development in *Ldlr*-deficient mouse embryos, and most widely used murine atherosclerosis models rely on combined *Ldlr* and *ApoE* deficiency, which complicates interpretation of LDLR-specific developmental effects.

Given these constraints, and the unique developmental tractability of the zebrafish model, we focused our efforts on bridging zebrafish and human endothelial systems to assess conserved stress- and flow-responsive programs. We continue to actively seek opportunities to analyze human endothelial cells from bone fide LDLR loss-of-function patients to further extend these findings.

6. Multiple sets of data show that phenotypes appear and then normalize in the zebrafish over time. For instance, there is a stark increase at 2 dpf of apoptosis, apolipoprotein B particles, PCNA cilia length and other phenotypes, but these resolve by 3 dpf. This is puzzling and not sufficiently rationalized. The authors suggest there is a 'normalization of CVP EC turnover'. Are the stressed cells dead at this point? Or are the authors certain that this is not genetic compensation by other pathways that might compensate for ldlr loss?

We agree with the reviewer that the developmental phenotypes we describe occur within a relatively narrow temporal window and that their partial normalization requires careful interpretation. Our data indicate that multiple abnormalities converge at 2 dpf, after which some, but not all, phenotypes normalize, suggesting differences in the rates of flux through distinct cellular and molecular processes rather than a uniform resolution mechanism.

While endothelial *hsp70* expression normalizes from 3 dpf onward, this does not reflect an overall recovery of vascular phenotypes. Notably, vascular apoptosis and ER stress elevation persists at 3 dpf, prior to LDL-cholesterol elevation in *ldlr*-/- zebrafish at 5 dpf.

Our inference of normalized CVP endothelial cell turnover at 2 dpf is based on the ratio of proliferative (PCNA+) to apoptotic (TUNEL+) cells rather than on absolute resolution of stress. Due to technical limitations with co-staining *hsp70*+ endothelial cells alongside other vascular markers, we cannot definitively determine whether *hsp70*+ cells preferentially undergo apoptosis or proliferation. However, supporting evidence from our scRNA-seq pseudotime trajectory analysis suggests that peak, flow-induced *hsp70* expression is associated with reduced expression of genes involved in mitotic nuclear division, consistent with a transient stress-adapted endothelial state rather than a hyperproliferative one.

Furthermore, new data acquired during manuscript revision support a spatial coupling of disturbed CVP flow, increased endothelial apoptosis, *hsp70*+ ECs and abnormal vascular remodeling, suggesting that these processes are mechanistically linked rather than independently resolving.

Based on these findings, our working model is that *hsp70*+ endothelial cells are dose-dependently induced by cell stress and blood flow as part of an intracellular, cytoplasmic chaperone response. This response appears to be partly adaptive, promoting endothelial survival, yet simultaneously maladaptive in the developmental context by disrupting CVP patterning and ciliogenesis.

We have summarized this *in vivo* model schematically in **Figure 7**. Briefly:
– At 1 dpf, *ldlr*-/- embryos exhibit elevated ER stress signaling and vascular apoptosis.
– At 2 dpf, disturbed CVP flow coincides with acute induction of *hsp70*+ ECs, sustained endothelial apoptosis, loss of endothelial ciliogenesis, abnormal remodeling, and expansion of surrounding immune populations.
– At 3 dpf, some vascular features partially normalize, while endothelial apoptosis, ER stress signaling, and immune cell expansion persist.

We also acknowledge other mechanisms may contribute to the phenotypic changes and include these in the Discussion text, outlining areas for future exploration.

7. There is no difference in the head vessel apoptosis, yet increased apoptosis line in the trunk. Is there a difference between head and trunk lipoprotein metabolism? Along the same lines, why are the DLAV and CVP more affected, as opposed to other vessels? Could this be a flow dependent effect?

We thank the reviewer for raising this question regarding heterogeneity in vascular apoptosis. In our imaging datasets, cerebral vascular patterning did not show clear abnormalities between genotypes, and our analyses support that cerebrovascular apoptosis was not elevated in *ldlr*-/- zebrafish. For this reason, we focused our mechanistic investigation on the trunk vasculature, where phenotypes were more pronounced.

Differences in hemodynamic exposure may contribute to regional phenotypic differences between the head and trunk. Published measurements indicate that RBC velocities in cranial vessels at 2 dpf average around 200-300 um/sec (Chen et al., 2012, PMID: 22904685), which is higher than the CVP and DA velocities we measured at the same developmental stage (**Figure 1D**). Although lipoprotein particles likely flow through the cerebral circulation, higher flow velocities may limit endothelial exposure time and reduce susceptibility to stress-induced remodeling or apoptosis in these vessels.

With respect to the DLAV and CVP, these vessels have been characterized to experience lower levels of shear stress flow at same developmental stages in prior hemodynamic modelling and live imaging studies (Ye et al., 2022, PMID: 35795647). This is consistent with our new RBC tracking data, together suggesting a disturbed flow-dependent contribution to the regional vulnerability of trunk vessels. We acknowledge that flow alone is unlikely to fully explain the observed heterogeneity. Distinct developmental trajectories and metabolic states may further shape vessel-specific responses to *ldlr* loss of function and we now expand on these points in the Discussion.

8. The idea that there is a connection between hair cells and endothelial cilia is not supported by any analysis in any other ciliated cell type in the zebrafish. There likely is no connection other than the cells are ciliated. If the authors wish to keep this statement, they should show that the phenotype is unique to these two cell types and not other ciliated cells.

We agree with the reviewer and have included a more extensive response (including new analyses) to a similar comment from reviewer 3 (point #2). In summary, we examined known hair cell genes in our zebrafish endothelial scRNA-seq dataset and found limited enrichment of canonical hair cell markers in endothelial populations. Conversely, analysis of the most significantly enriched genes in the *hsp70+* endothelial subpopulation identified some overlap with neuromast hair cells, notably including the axonemal gene *pierce1* (si:ch211−248e11.2) and the molecular chaperone *hsp90aa1.2*. Cross-referencing with a published neuromast scRNA-seq dataset (Baek et al., 2022, PMID: 35316618) confirmed that these genes are preferentially expressed in hair cells rather than support cells, suggesting shared sensitivity to cytoskeletal or cellular stress (**Figure 2 for reviewer**).

Based on this, we have de-emphasized this interpretation in the main text and moved all neuromast-related data to Supplementary Materials. We have instead further discussed endothelial flow sensitivity and how other canonical mechanosensors could be affected by loss of ldlr function, including other membrane complexes such as caveolae.

9. The supplied word file for the supplementary data is at unacceptable resolution. The reviewer assumes this was a mistaken upload. This must be corrected.

We thank the reviewer for bringing this to our attention. We have now reformatted and reuploaded all supplementary figures at higher resolution.

10. Figure 1, panel c. For ciliated pillar, show boxes to show insets (XZ and YZ). Where is the pillar?
We have now added boxed regions in Figure 1C to indicate the locations of the XY, YZ and XZ insets. In these insets we now also indicate the CVP loops.

11. Fig 1 Graphs have different axes, and axes don't start at 0. It is very hard to compare different timepoints due to the changing axes. If the 2 and 3 dpf data on the same graph, it would give perspective on how parameters change over time.
We have now merged the 2 dpf and 3 dpf data for each CVP parameter into the same graph to facilitate direct comparison across developmental time points.

12. Fig 5: graph in D, top right is missing the legend below the graph.
We have now corrected this and added the missing figure legend.

13. Have the authors tested whether any of the phenotypes are cholesterol-dependent? Do statins rescue the early phenotypes?

As part of our response to reviewer 1, we performed additional experiments testing the effects of two clinically used LDL-cholesterol lowering statin therapies, atorvastatin and simvastatin, on early vascular development. Dose-response experiments found that both atorvastatin and simvastatin induced toxicity in both *ldlr*+/+ and *ldlr*-/- embryos at nanomolar dose concentrations. Intravital vascular imaging demonstrated dose-dependent defects in angiogenesis, including CVP regression and vascular blebbing suggestive of endothelial cell death at 50nM for atorvastatin and 100nM for simvastatin. Notably, 100nM atorvastatin resulted in embryonic lethality, consistent with its higher potency. Defects on CVP patterning and intracranial haemorrhage were observed at 5nM and 10nM for atorvastatin and simvastatin, respectively. These findings indicate that statin-induced toxicity confounds the interpretation of vascular developmental phenotypes in this context.

In parallel, we measured LDL/VLDL levels in *ldlr*+/+ and *ldlr*-/- zebrafish at 2 dpf and 5 dpf. At 2 dpf, whole-body LDL/VLDL levels were low and not significantly different between genotypes. By contrast, at 5 dpf LDL/VLDL levels increased developmentally in both groups, with *ldlr*-/- larvae exhibiting a further significant elevation compared to their *ldlr*+/+ counterparts, consistent with prior reports (Liu et al., 2018, PMID: 29187523). The new data are now included the revised manuscript (**Figure S1B**).

14. The one experiment that may not meet field standards is the CRISPR F0 analysis of hsp70 mutation. This experiment is backed up by chemical inhibitor data, but we believe that the zebrafish field standard is that the

data should be backed up by a germline knockout due to possible transient effects due to the CRISPR knockout (i.e. stress effects, which would be relevant to this manuscript topic). Can the authors please address this?

While there are rigorous data (albeit for different pathways) that generally support strong parallels between F0 and F3 CRISPR data, we agree that it would be preferable to use stable CRISPR-generated *hsp70* mutant zebrafish. However, in this specific case of *hsp70*, generation of a viable germline mutant presents substantial technical and biological challenges. We observed approximately 70% of F0 *hsp70* crispants survived up to 2 dpf in both *ldlr*+/+ and *ldlr*-/- backgrounds, but these embryos consistently exhibited overt developmental abnormalities, including oedema and body axis deformities, suggesting that loss of *hsp70* compromises viability and is unlikely to support survival to adulthood for breeding.

Second, for each F0 CRISPR experiment, embryos used for phenotypic analysis were genotyped to confirm the presence of predicted *hsp70* deletions, while control-injected embryos lacked detectable mutations (**Figure S19A**).

Finally, since *hsp70*+ ECs are acutely induced at 2 dpf in *ldlr*-/- embryos, the later temporal inhibition of *hsp70* using the pharmacological inhibitor VER-155008 (between 1-2 dpf) allowed us to separate effects on the vasculature from overt developmental defects.
